# Immunotherapy in soft tissue and bone sarcoma: unraveling the barriers to effectiveness

**DOI:** 10.7150/thno.72800

**Published:** 2022-08-15

**Authors:** Myrofora Panagi, Pampina Pilavaki, Anastasia Constantinidou, Triantafyllos Stylianopoulos

**Affiliations:** 1 Cancer Biophysics Laboratory, Department of Mechanical and Manufacturing Engineering, University of Cyprus, Nicosia, Cyprus.; 2 Medical School, University of Cyprus, Nicosia, Cyprus.; 3 Bank of Cyprus Oncology Centre, Nicosia, Cyprus.; 4 Cyprus Cancer Research Institute, Nicosia, Cyprus.

**Keywords:** tumor microenvironment, immunosuppression, hypoxia, tumor normalization, mechanotherapeutics

## Abstract

Sarcomas are uncommon malignancies of mesenchymal origin that can arise throughout the human lifespan, at any part of the body. Surgery remains the optimal treatment modality whilst response to conventional treatments, such as chemotherapy and radiation, is minimal. Immunotherapy has emerged as a novel approach to treat different cancer types but efficacy in soft tissue sarcoma and bone sarcoma is limited to distinct subtypes. Growing evidence shows that cancer-stroma cell interactions and their microenvironment play a key role in the effectiveness of immunotherapy. However, the pathophysiological and immunological properties of the sarcoma tumor microenvironment in relation to immunotherapy advances, has not been broadly reviewed. Here, we provide an up-to-date overview of the different immunotherapy modalities as potential treatments for sarcoma, identify barriers posed by the sarcoma microenvironment to immunotherapy, highlight their relevance for impeding effectiveness, and suggest mechanisms to overcome these barriers.

## Introduction

Sarcomas are a group of rare and highly heterogeneous malignancies encompassing more than 100 distinct histological subtypes 1, 2. They can be broadly divided into two categories: soft tissue sarcomas which develop from fat, muscle, blood vessels, nerves, and other connective tissues, and bone sarcomas. The overall incidence rate for soft tissue sarcoma ranges between 4 and 5 cases per 100,000/year, with liposarcoma and leiomyosarcoma being the most common subtypes 3. Bone sarcoma is less prevalent with an estimated incidence of 0.8 cases per 100,000/year, with osteosarcomas being the most common, followed by conventional chondrosarcoma and Ewing sarcoma 4.

Despite recent advances in cancer research, developments in sarcoma treatment have been slow. For localized disease, the primary treatment option is surgical resection of the tumor with adjuvant or neoadjuvant radiation therapy in selected cases. Localized soft tissue sarcoma and bone sarcoma patients have a 5-year disease free survival rate >80 % and 70 %, respectively. However, disease relapse occurs in more than half of the patients, often with the development of distant metastasis. Advanced disease cases are extremely challenging to treat. Conventional chemotherapeutics do not lead to durable responses or cure and patients may experience substantial toxicities. The standard therapy for metastatic disease is primarily structured around anthracycline-based chemotherapy 3, 4, while other agents including dacarbazine 5, gemcitabine/docetaxel 6, ifosfamide 7, trabectedin 8, pazobanib 9 and eribulin 10 might also be used. The median overall survival of patients with metastatic disease ranges from 12 to 18 months 11, 12. Therefore, with limited success of conventional chemotherapy for sarcoma in clinical practice, there is a high unmet need to develop novel therapeutic strategies with improved efficacy and safety for these patients.

A flurry of new research is now exploring the role of immunotherapy in sarcoma. Unlike chemotherapy, which directly kills cancer cells, immunotherapy relies on stimulating the natural defenses of the host immune system to attack malignant cells. Modalities of immunotherapy can be grouped in the following clusters: immune checkpoint inhibitors (ICIs), adoptive cell therapy (ACT), cancer vaccines and *in situ* vaccination (iSV) including oncolytic virotherapy. Indeed, there is a large number of clinical trials for the use of immunotherapy in patients with sarcoma, but none of them has led to approval yet. The absence of dramatic immunotherapeutic responses in most cases has been attributed to a variety of factors, including barriers imposed by the tumor microenvironment that among others hinder the delivery of the immunotherapeutic agents and cause immunosuppression. The current review i) summarizes current knowledge on immunotherapy application in soft tissue and bone sarcoma in the clinical and preclinical setting over the last decade, ii) discusses the barriers posed by the sarcoma microenvironment hindering immunotherapy efficacy and iii) provides an overview of potential strategies that are tailored to overcome these barriers.

## Immunotherapeutic strategies in clinical cancer treatment

### Immune checkpoint inhibition

Immune checkpoints have evolved to act as gatekeepers of immune responses by suppressing inflammation-induced tissue damage and autoimmunity. However, cancer cells hijack the immune checkpoint signaling by upregulating inhibitory immunoreceptors (e.g., PD-1, CTLA-4, LAG3, TIM3, TIGIT and BTLA) on immune cell surface, capable of suppressing both antigen and co-stimulatory signaling upon ligand engagement 13 and thus, allowing tumor-cells to escape surveillance from both the adaptive and innate immune system. Monoclonal antibodies directed against immune checkpoint molecules, known as immune checkpoint inhibitors (ICIs), comprise the most advanced paradigm of immunotherapy in the clinical setting, managing to prolong overall survival of patients with melanoma 14-16, triple negative breast cancer 17, non-small cell lung cancer 18, renal cell cancer, Hodgkin's lymphoma, urothelial cancer and mismatch repair deficient /microsatellite instability high (MSI-H) tumors 19-23. The currently approved ICIs by FDA inhibit the cytotoxic T lymphocyte-associated molecule-4 (CTLA-4, e.g., ipilimumab, tremelimumab), the programmed cell death protein 1 (PD-1, e.g., nivolumab, pembrolizumab) and the programmed cell death ligand (PD-L1, e.g., atezolizumab, avelumab, durvalumab).

With regards to sarcoma patients, blockade of the PD-1/PD-L1 axis has been promising for specific histological subtypes. A completed phase 2 clinical trial, SARC028 (NCT02301039, Table 1) investigating the use of pembrolizumab for the treatment of advanced sarcoma, demonstrated an objective response in seven out of the forty patients (18%) with soft-tissue sarcoma and partial response in two of the forty patients (5%) with bone sarcoma. Response was determined by investigators using RECIST version 1.1. In the soft tissue sarcoma patients' cohort, the benefit was limited to four out of ten patients with undifferentiated pleomorphic sarcoma, two out of ten patients with dedifferentiated liposarcomas and one out of ten patients with synovial sarcoma. No clinical benefit was observed in leiomyosarcoma patients. In the bone sarcoma cohort, one out of twenty two patients with osteosarcoma and one out of five patients with chondrosarcoma had an objective response, while none of the thirteen patients with Ewing sarcoma responded to immune checkpoint inhibition. Notably, a positive correlation between PD-L1 expression and therapeutic outcome was established in three patients with undifferentiated pleiomorphic sarcoma. Of the three patients, one had a complete response and the other had a partial response. Consistent with previous studies 24, 25, undifferentiated pleomorphic sarcoma patients evaluated for response by RECIST.1.1 criteria had higher numbers of activated T cells, increased PD-L1 expression on immunosuppressive tumor associated macrophages (TAMs) and more regulatory T cells (Tregs), compared to non-responders prior to PD-L1 inhibition 26. These findings suggest that undifferentiated pleomorphic sarcoma may fit the model of an inflamed tumor responding to PD-L1 inhibition. Moreover, Alliance A091401 (NCT02500797, Table 1), is another phase 2 clinical trial investigating the use of nivolumab versus nivolumab plus ipilimumab in patients with sarcoma (soft tissue or bone sarcoma). Confirmed responses defined as complete or partial response by RECIST1.1 were reported in two (5%, one with alveolar soft part sarcoma and one with non-uterine leiomyosarcoma) out of the thirty eight patients in the monotherapy group and in six (16%) out of the thirty eight patients in the combination group, particularly in those diagnosed with undifferentiated pleomorphic sarcoma, leiomyosarcoma, myxofibrosarcoma, malignant fibrous histiocytoma and angiosarcoma. Interestingly, the proportion of confirmed objective responses in the combination treatment group is comparable to that obtained with front-line standard chemotherapy, thus highlighting the potential of nivolumab plus ipilimumab as a first-line therapy alternative.

Although no treatment-related deaths were documented, adverse effects associated to immunotherapy including anaemia, decreased lymphocyte count, dehydration and other were reported in both treatment arms. Alliance was the first study to investigate the combination of checkpoint inhibitors and demonstrated promising efficacy results in certain sarcoma subtypes 27. The rationale behind this combination was based on ipilimumab's effect to increase T cell activation and thus, allow nivolumab to augment anti-tumor T cell responses. Consistent with the SARC028 study, expression of genes implicated in antigen presentation and T cell infiltration was higher in undifferentiated pleomorphic sarcoma and leiomyosarcoma compared to synovial sarcoma and liposarcoma. Overall, discrepancy to ICI efficacy seems to rely on the pre-existing immunogenicity of the tumor microenvironment (TME). However, the small number of biopsies collected during, before and throughout treatment and the subsequent characterization of TME composition and infiltration of lymphocytes among responders, limit the comprehensive analysis of biomarkers. Determination of baseline antitumor immunity of responders before treatment is required to understand the infrequent responses and mechanisms of resistance in tumor subtypes, in which rational combination therapies could be considered. Accordingly, current clinical findings postulate that a TME deprived of infiltrating lymphocytes is less likely to benefit from such a treatment modality 28. Other factors that impact ICI therapy include checkpoint expression status, tumor microsatellite instability (MSI) and tumor mutational burden 29-32. A summary of clinical trials employing ICIs for sarcoma are listed in Table 1.

### Adoptive cell therapy

ACT, also known as cellular immunotherapy, comprises the intravenous transfer of either tumor-resident or genetically modified blood-derived immune cells into patients to augment antitumor immune responses. The most widespread form of ACT is T cell based and can be classified into (i) ACT with **tumor-infiltrating lymphocytes** (TIL) and (ii) ACT with genetically **engineered T cell receptor** (TCR) or synthetic **chimeric antigen receptor** (CAR) targeting tumor specific antigens 33, 34. A consistent success of TIL therapy has been demonstrated only in melanoma patients, whereas production and reactivity of TILs from other solid tumor types has led to variable antitumor responses, presumably due to the highly heterogenic mutational and neoantigen load 34-36. Administration of TIL therapy combined with adjuvant chemotherapy was found to significantly prolong the survival of osteosarcoma patients with a poor response to neoadjuvant chemotherapy, compared to the patients receiving adjuvant chemotherapy. In addition, univariate and multivariate analyses indicated that a greater number of TILs transfused as an independent prognostic factor for both the median disease-free survival and overall survival 37.

With respect to TCR-modified cell therapy, an affinity-enhanced TCR recognizing the cancer testis antigen, NY-ESO-1, reported encouraging results for the treatment of metastatic synovial sarcoma (Table 1), confirming antitumor responses in half of the patients 38. Further investigation showed that the response was associated with modest increase in intratumoral leukocyte infiltration and minimal infiltration of CD163^+^ TAMs, compared to the pre-infusion state 39.

Conversely to TCR-modified T cells, CAR T cells can induce conventional activation signals from TCRs in an MHC-independent manner. Notably, CAR T therapy is applied in hematological neoplasms producing remarkable and durable responses, while its application in solid tumors has been rather unsatisfactory 40. Evaluation of safety and efficacy of tumor-directed T cells in sarcoma patients is in the early stages of clinical development. To date, T cells expressing a HER2-specific chimeric antigen receptor with a CD28.ζ signaling domain (HER2-CAR T cells) have been evaluated in a phase 1/2 clinical study in patients with HER2 positive sarcomas confirming that these cells can persist for six weeks without evident toxicities (Table 1). No follow up of this study or additional clinical documentation regarding CAR T cell therapy has been reported since then 41. Potential barriers include the insufficient T cell penetration into solid masses due to physical obstacles, the lack of targetable antigens solely expressed on tumor cells and the limited CAR T cell persistence after infusion and homing of potent immunosuppressive cells that tender T cells dysfunction in the TME 42. An alternative and less complex approach of ACT is the CAR-NK cell therapy 43, 44. Clinical success of CAR-NK cell therapy has been initially demonstrated in hematological cancers 45, 46 and currently is being evaluated in solid malignancies including sarcoma (NCT02100891).

### Cancer vaccines

Cancer vaccines involve the exogenous administration of selected tumor associated antigens (TAA) combined with adjuvants. The ultimate goal is to provoke an adaptive T cell response capable of eradicating residual tumor and establishing a lasting antitumor memory in the absence of adverse effects and non-specific reactions. Traditionally, antigens can be delivered in the form of DNA, RNA and peptide itself, or via autologous dendritic cells (DCs). Administration of autologous DCs matured with autologous tumor lysate and keyhole limpet hemocyanin was found to improve clinical outcome in patients diagnosed with Ewing/rhabdomyosarcoma, whereas other histological subtypes failed to respond. Of note, T cell responses to autologous tumor lysate were identified in more than half of immunotherapy recipients, in agreement with a higher survival, while an enhanced immune reconstitution was reported after addition of interleukin 7 (IL7) 47. In line with these results, vaccination of metastatic Ewing sarcoma patients with Vigil vaccine (GMCSF/bi-shRNAfurin DNA-transfected autologous tumor immunotherapy) resulted in improved overall survival compared to the unvaccinated group 48. On the contrary, autologous tumor lysate pulsed dendritic cell vaccination of patients with bone and soft tissue sarcoma showed minimal clinical effectiveness, although the reported increased levels in IFNγ and IL12 post-vaccination 49, 50. Representative clinical trials using cancer vaccines for soft tissue and bone sarcomas are listed in Table 1.

### *In situ* vaccines

iSVs comprise a novel arm of cancer immunotherapy. As opposed to conventional vaccines, iSVs are antigen-agnostic agents having the ability to mount endogenous antitumor responses, directly or indirectly, by “generating” a vaccine within the TME following sourcing of antigens from dead or dying tumor cells. The mechanisms of action of iSVs mainly rely on the activation of innate immune pattern recognition receptors (e.g., TLRs) and stimulator of interferon genes (STING) protein 51. The antitumor efficacy of iSV agents as monotherapy or combination therapy is now evaluated in different solid tumors, including sarcomas. Some intrinsic examples of these studies are the combination of a Poly-ICLC agonist for RIG-I/MDA5 and TLR3 with anti-PD-1 or anti-PD-L1 treatment (NCT02423863) and the combination of TLR4 agonist G100 with anti-PD-1 plus metronomic cyclophosphamide in patients with advanced sarcomas (NCT02406781).

Another class of recently introduced iSV agents are the **oncolytic viruses**. Genetically or chemically modified oncolytic viruses expressing immunomodulators specifically infect and replicate within cancer cells inducing immunogenic cell death and consequent release of TAA and neoantigens. This process results in local priming of the immune system (recruitment of DCs and NK cells to the tumor site), leading to an effective rejection of both virus-injected and distant tumors 52. To date, talimogene laherparepvec (T-vec), a genetically modified GM-CSF-expressing herpes simplex virus, is the first and only oncolytic immunotherapy approved by the FDA for the treatment of advanced melanoma 53. A phase 2 clinical trial investigating the efficacy of T-vec with pembrolizumab for sarcoma treatment demonstrated a benefit only in specific subtypes. Specifically, partial responses were seen in patients with cutaneous angiosarcoma of head and neck, undifferentiated pleomorphic sarcoma, myxofibrosarcoma, epithelioid sarcoma and in one case with unclassified sarcoma. Importantly, the immune microenvironment of responders correlated with an upregulation of PD-L1 expression and higher TIL content (NCT03069378, Table 1) 54.

## Lessons from preclinical studies

Despite the rapid advances made in the field, immunotherapy for sarcoma treatment is still in its infancy. The rarity, heterogeneity and complexity of these mesenchymal malignancies create challenges around diagnosis and treatment decisions. Thus, significant attention should be paid during trial design and interpretation of results. An approach to improve clinical trial design and eliminate uncertainties about the treatment effect in such rare diseases is the consideration of comprehensive preclinical evidence that strongly supports the effectiveness of a particular therapeutic intervention for a specific sarcoma subtype.

### Landscape of immunotherapy in soft tissue sarcoma

**Figure 1:**
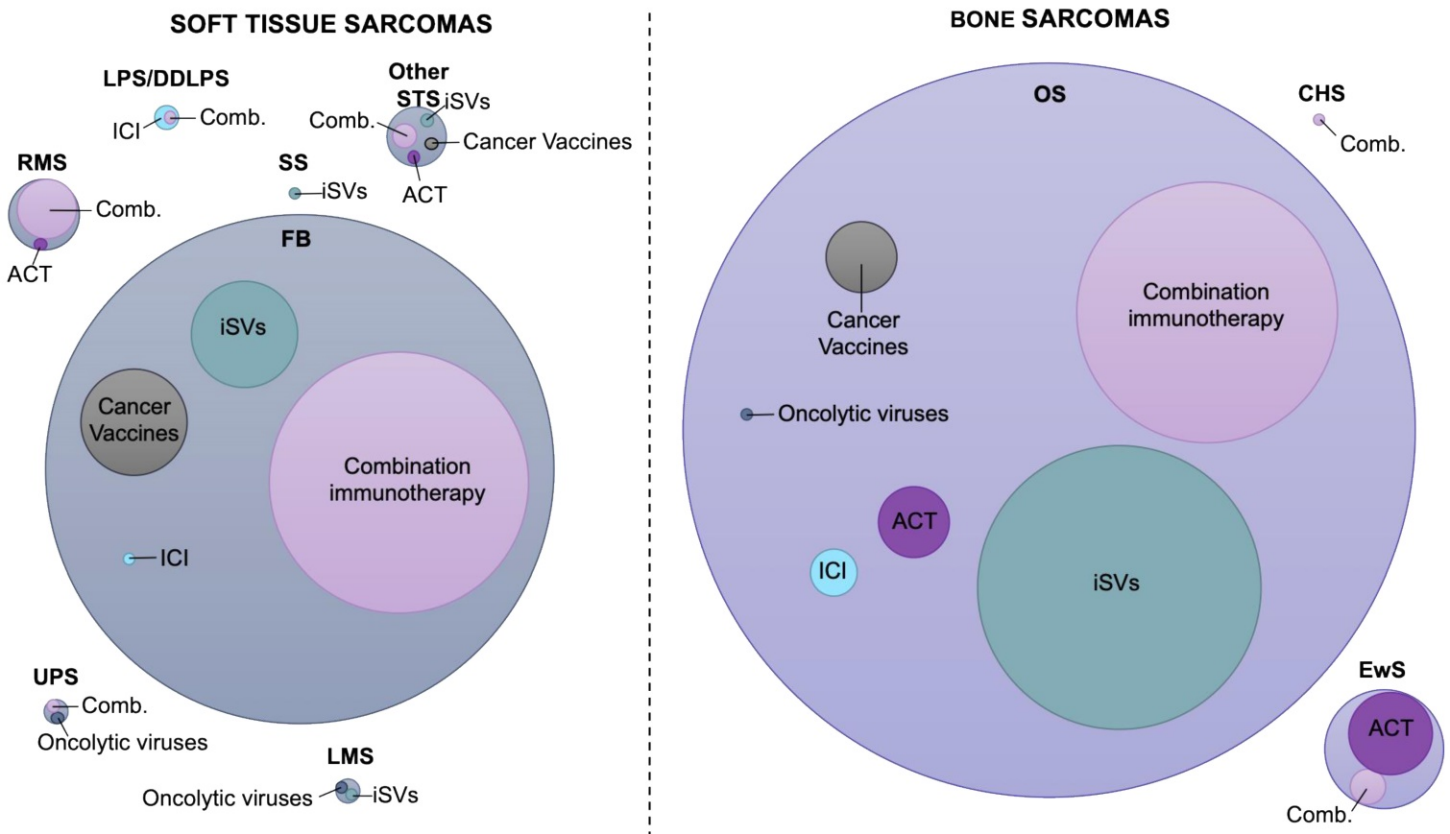
Landscape of immunotherapy application in sarcoma treatment. Visualization of the number of studies performed from 2011 to 2021 for each sarcoma subtype, assessing the efficacy of different immunotherapy modalities in the preclinical setting. The circle diameter indicates the relative proportion of preclinical studies identified. Combination circle includes combinations between two or more immunotherapy modalities or combinations with chemotherapeutics or radiation therapy. The data included in this figure are listed in Tables 2 and 3 and Tables S1 and S2. FB, fibrosarcoma; DDLPS, dedifferentiated liposarcoma; LPS, liposarcoma; RMS, rhabdomyosarcoma; LMS, leiomyosarcoma; SS, synovial sarcoma; UPS, undifferentiated pleomorphic sarcoma; Other STS, undefined type of STS; EwS, Ewing sarcoma; BS, bone sarcoma; CHS, chondrosarcoma; ICI, immune checkpoint inhibitors; ACT, adoptive cell therapy; iSVs, *in situ* vaccines

**Fibrosarcoma** is the most frequently employed tumor model in the preclinical setting with regards to soft tissue sarcoma immunotherapy (Figure 1). Multiple immunotherapy approaches have been investigated with ICI and cancer vaccines being the most extensively studied, often in combination with adjuvants (Table 2, Table S1). An intrinsic example is the dual targeting of co-inhibitory CTLA-4 and costimulatory OX40 signaling pathways. Ligation of the TNF receptor family OX40 (CD134) with the agonist anti-OX40 led to enhanced antitumor immunity by augmenting effector T cell differentiation and suppressing the activity of Tregs 55. With reference to tumor specific antibody-cytokine fusion proteins, L19 antibody (targeting fibronectin)-mIL12 construct was found to induce a strong antitumor effect against fibrosarcoma tumors only after combination with PD-1 inhibition. Neither PD-1 or CTLA-4 monotherapy or anti-CTLA-4-L19-mIL12 combination therapy had any impact on tumor control 56. Another promising therapeutic intervention implicates DC-based vaccination and targeting of HSP90 via the 17-DMAG inhibitor. Binding of 17-DMAG to HSP90 results in the degradation of the HSP90 client protein EphA2, a receptor tyrosine kinase which is highly upregulated in a variety of cancers and correlates with poor prognosis and metastasis 57. Proteasomal degradation of EphA2 promoted MHC class I presentation of the derivative peptide epitopes and their subsequent recognition by specific CD8^+^ T cells leading to sarcoma regression. Nevertheless, when coordinated with vaccination, 17-DMAG co-administration yielded superior antitumor efficacy capable of rendering animals free of disease, as opposed to treatment with either single modality 58. In a different study, fibrosarcoma tumors refractory to doxorubicin chemotherapy partly due to weak expression of nuclear HMGB1, exhibited higher response rates in the presence of the synthetic TLR4 agonist, Dendrophilin. The synergistic antitumor effects were attributed to the enhanced DC-dependent T cell priming mediated by restoration of the immunogenicity of dying tumor cells and increased intratumoral accumulation of IFNγ^+^ lymphocytes 59.

Mimicking the clinical scenario, the efficacy of ICI in **rhabdomyosarcoma** is yet to be determined. Nevertheless, alternative strategies have been developed including ACT using NK 60 or genetically engineered T cells 61, DC-based vaccination 62, 63 or virotherapy 64, all administered with various iSVs. A more recent study assessed and confirmed the antitumor efficacy of CAR-modified cytokine-induced killer cells as an alternative type of effector cells 65. Cytokine-induced killer cells are a heterogeneous population of effector NKT cells which can be easily expanded from peripheral blood mononuclear cells and subjected to genetic engineering to express CARs. Accordingly, administration of CAR cytokine-induced killer cells directed against the TAA ERBB2 led to a complete inhibition of initial tumor load and microscopic clearance of tumors mediated by enhanced accumulation of NK and NKT cell subpopulations in disseminated rhabdomyosarcoma. On the contrary, non-targeted cytokine-induced killer cell therapy exhibited a partial tumor inhibition 65. Therefore, the dual role of cytokine-induced killer cells as targeted killers and modulators of innate immunity in parallel with the diverse T and NK cell receptor specificities, make them an attractive platform with considerable potential to improve the clinical outcome of sarcoma patients.

Leiomyosarcomas, liposarcomas and undifferentiated pleomorphic sarcomas are among the most common soft tissue sarcomas and yet, the less studied in the preclinical setting, at least in the context of immunotherapy 1. An early attempt to harness the host's immune system to cure aggressive metastatic **leiomyosarcoma** involved the targeting of CD47 protein. Mice receiving anti-CD47 treatment experienced significant tumor size reduction and regression of metastatic disease mainly via stimulating macrophage dependent phagocytosis 66. A later study evaluated the efficacy of oncolytic immunotherapy using a serotype chimeric oncolytic adenovirus coding for the human GM-CSF, Ad5/3-D24-GMCSF. Ad5/3-D24-GMCSF treatment not only circumvent off-target toxicity associated with the systemic use of GM-CSF, but also exhibited a potent antitumor activity which was further validated in soft tissue sarcoma patients 67.

Initial preclinical work on **undifferentiated pleomorphic sarcoma** exploited the antitumor activity of patient derived cytokine-induced killer cells demonstrating that intravenous infusions of cytokine-induced killer cells could cause a significant delay of tumor growth and facilitate killing of putative sarcoma cancer stem cells 68. Later, new evidence emerged showing that inhibition of retinoic acid signaling synergizes with anti-PD-1 treatment increasing the frequency of immunostimulatory antigen presenting cells (APCs) and engendering tumor regression. Consistent with its pro-tumor activities, the pertinent study showed that T cell-derived IL13 can induce retinoic acid production by sarcoma cells which in turn inhibits tumor monocyte differentiation into DCs and promotes generation of immunosuppressive macrophages 69. Retinoic acid plays a crucial role in shaping the tumor immune microenvironment acting both as an anti- and pro-tumor agent 70. While many studies have reported that retinoic acid supports immune tolerance via suppressing the differentiation of monocyte derived DCs 71, promoting the differentiation of Tregs 72 and Arg1 producing anti-inflammatory macrophages 73, 74, others have reached the opposite conclusion 75, 76. As such, a more thorough investigation is required to support the antitumor effects of retinoic acid signaling inhibition across soft tissue sarcoma histological subtypes.

Preclinical studies on animal models of **liposarcoma** have just commenced. To date, there are only a few available studies both exploring the antitumor efficacy of PD-1 inhibition as a monotherapy 77 and its combinatorial effect with the oncolytic vaccinia virus (GLV-1h68) 78, in a model of dedifferentiated liposarcoma and liposarcoma (of unspecified histological subtype), respectively. Notably, anti-PD-1 treatment significantly slowed tumor growth by promoting accumulation and activity of CD8^+^ T cells and NK cells 77. Considering dual treatment, pretreatment with GLV-1h68, delivered using isolated limb perfusion (viral ILP), potentiated antitumor responses of PD-1 blockade, which had a minimal efficacy as a single modality. Moreover, when performed prior to compartmentectomy and radiotherapy, combined treatment prevented both local and distant relapse 78.

**Synovial sarcoma** is another example of most common and refractory to treatment soft tissue sarcomas. Surgical resection, accompanied by radiation and/or chemotherapy, has shown to be effective only during early stages of the disease while no successful therapies have been established for advanced synovial sarcoma, so far. In addition, the application of ICI in the synovial sarcoma treatment as part of clinical trials has not been successful (NCT02301039, NCT02304458). Nevertheless, emerging preclinical work reported that targeting of FZD10 combined with radioimmunotherapy exhibited a potent antitumor activity against xenograft tumors. Specifically, it was shown that labeling of anti-FZD10 antibody with α-emitting radionuclides is superior to β-emitters labeling with no apparent systemic toxicities. FZD10 is highly expressed in synovial sarcoma and serves as a putative receptor of Wnt signaling and thus, a promising target for iSV 79.

### Landscape of immunotherapy in bone sarcoma

ICI is currently the most prominent immunotherapy modality for **osteosarcoma** treatment in clinical practice. Although the clinical benefit from ICI alone is rather sporadic, preclinical studies indicate that combinations with other modalities of immunotherapy can significantly potentiate their antitumor responses (Table 3, Table S2). An intrinsic example is the combination of PD-1 blockade with the CXCR4 antagonist, AMD3100, which facilitates SDF-1/CXCR4 signaling inhibition. CXCR4 activation transmits signals that promote survival and accumulation of myeloid-derived suppressor cells (MDSCs) in osteosarcoma microenvironment and inhibiting cytotoxic T lymphocyte (CTL) trafficking and function and thus, blunting the response to anti-PD-1 therapy. ICI alone does not affect tumor growth and CXCR4 inhibition only modestly reduces its size. However, anti-PD-1 and AMD3100 co-administration synergistically expands infiltrating CTLs, enhances tumor growth control and prolongs survival 80. Similarly to CXCR4 antagonist, a variety of iSV agents have reported synergistic effects in the context of NK and T cell based ACT 81-85, DC vaccination 86, 87 and chemotherapy 88 or radiotherapy 89. Regarding cancer vaccines, CTLA-4 blockade was found to cooperate with cryotreated tumor lysate-pulsed DC vaccine in a primary tumor control to prevent the outgrowth of lung metastasis by reducing levels of Tregs and increasing infiltration of cytotoxic CD8^+^ lymphocytes inside the metastatic tumor 90. A more recent study has exploited the combination of either PD-1 or PD-L1 blockade with adoptive transfer of T cells armed with anti-GD2-BsAb (GD2-EATs) or anti-HER2-BsAb (HER2-EATs) 91. Both GD2 and HER2 are upregulated in osteosarcoma making them suitable targets. Interestingly, anti-PD-L1 combination treatment enhanced BsAb-armed T cell function and improved tumor control and survival of the mice, when given sequentially and continuously, while anti-PD-1 combination did not. The failure of anti-PD-1 combination might be partially explained by the upregulation in PD-L1 expression observed following BsAb treatment. Thus, providing hope for the treatment of metastatic or refractory osteosarcoma where clinical trials of anti-HER2 trastuzumab or anti-GD2 dinutuximab and ICI were unsuccessful.

The preclinical research activity for **Ewing sarcoma** is limited to ACT either as a monotherapy or upon combination with iSVs or virotherapy. The first study exploring the synergistic effects of ACT with iSV involved third generation GD2-CAR T cells, incorporating CD28 and OX40 costimulatory domains, engineered to recognize GD2 tumor antigen and all-trans retinoic acid (ATRA). The glycosphingolipid disialoganglioside GD2 is a well-known TAA implicated in tumor cell proliferation and currently explored in neuroblastoma, melanoma and sarcoma clinical trials (NCT02502786, NCT02484443) 92-94. ATRA is a clinically approved drug that eliminates immature myeloid cells by promoting their differentiation into a non-suppressive subtype and improves the effect of vaccination 95-97. With GD2-CAR T administration having a minimal antitumor effect, its combination with ATRA significantly reduced tumor volume and prolonged survival. ATRA treatment led to a potent granulocytic reduction in MDSCs compared to untreated control tumors and upregulated peripheral CTL levels following combination with ACT. Similar antitumor immune responses were observed in the osteosarcoma setting 85. Moreover, a novel upcoming combination for the treatment of pediatric Ewing sarcoma is that of activated and expanded NK cells with virotherapy. *In vitro* evidence indicated that co-culture of MeV-infected sarcoma cells with NK cells stimulated the release of GZMA/B, perforin and granulysin resulting in higher oncolysis rates when compared to the respective monotherapies 98.

Unlike osteosarcoma and Ewing sarcoma, preclinical evaluation of immunotherapy in **chondrosarcoma** is restricted to a single study. Consistent with the clinical application of zoledronate (ZOL), a nitrogen-containing bisphosphonate sensitizing tumors to Vγ9Vδ2 T cell-mediated cytotoxicity, *Sun et al.* showed that weekly intravenous ZOL administration improved Vγ9Vδ2 T cell cytotoxic in a TCR-dependent and via perforin-mediated mechanisms, resulting in potent antitumor effects 99, 100.

## Barriers to immunotherapy for soft tissue and bone sarcoma

The intrinsically heterogeneous nature of sarcomas and the complexity of the TME have a decisive role in their behavior and response to treatment. In the TME, tumor cells coexist with heterotypic cell populations including immune cells, endothelial cells, pericytes, cancer associated fibroblasts (CAFs), mesenchymal stem cells (MSCs) and nerve fibers, with which they communicate via direct cellular contact and an array of paracrine signals. Communication between cancer cells and stroma is also modulated by extracellular matrix components and the microbiome.

### Immune microenvironment barriers

**Figure 2:**
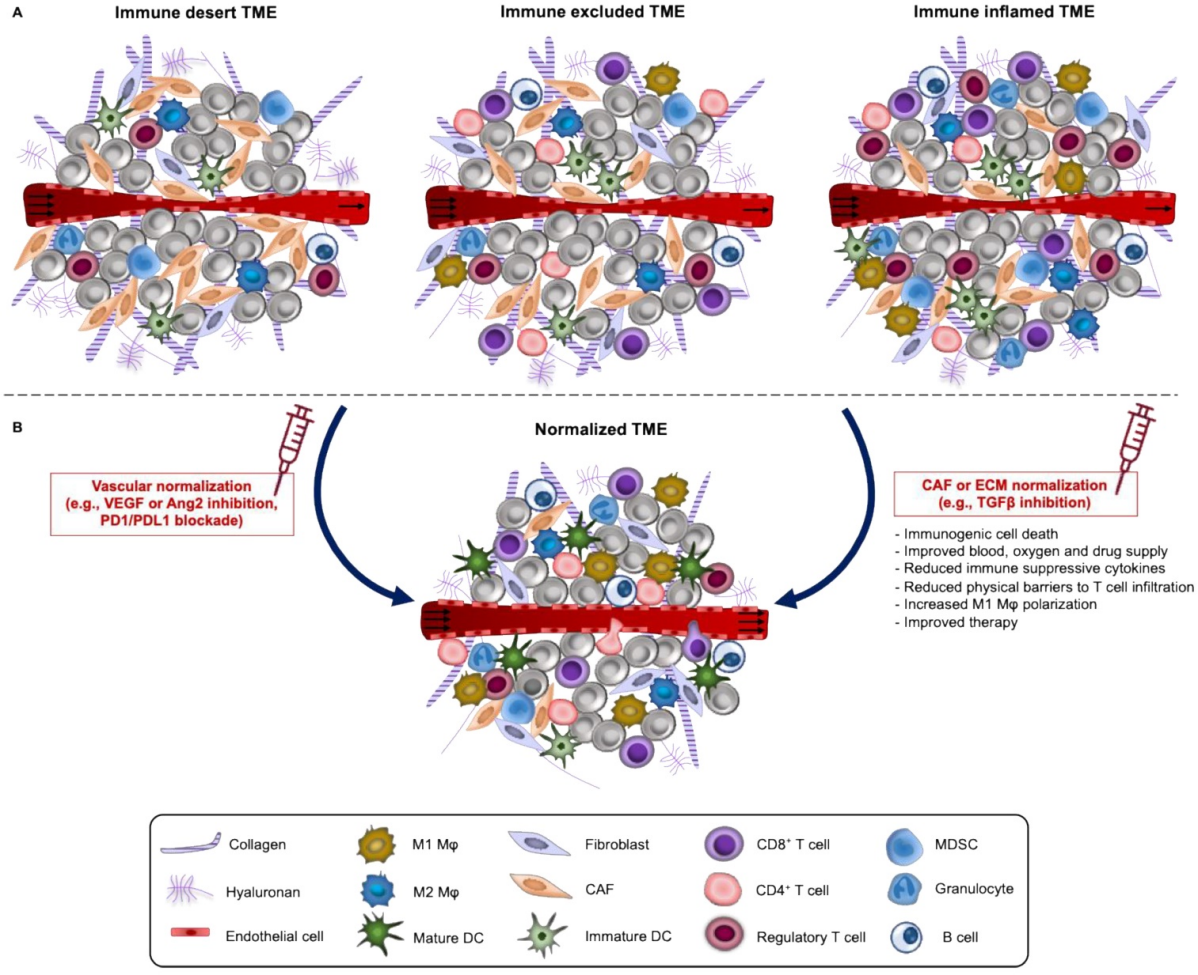
** A. Immune phenotypes of solid tumors and normalization strategies**. Based on the spatial distribution of CD8^+^ T lymphocytes in the TME, solid tumors are classified into highly **inflamed**- “**hot**” and non-inflamed- “**cold**”. “Cold” tumors like Ewing sarcoma can be further subdivided into **immune desert** or **immune excluded**. In “hot” tumors like most soft tissue sarcomas and osteosarcoma, T cells are present but inactive or exhausted. TME of “hot” tumors is defined by PD-1^+^ T cells and PD-L1^+^ tumor cells and macrophages, a high degree of tumor mutational burden and generally correlates with good response to ICI. In the immune desert phenotype, immune cells are absent from the tumor and its periphery while in the immune-excluded phenotype, immune cells accumulate at the periphery and do not efficiently infiltrate tumor bed. **B.** These three immune phenotypes can be reverted by TME normalization. TME normalization can be achieved by normalization of the tumor vasculature through targeting of angiogenic factors (such as VEGF and/or angiopoietin-2) and/or immune checkpoints (PD-L1, PD-1) and by normalization of the tumor ECM including reprogramming of CAFs to reduce fibrosis. These two normalization strategies either alone or in combination improve vessel perfusion, oxygen delivery, infiltration and activation of T cells and drug distribution.

Different immune cell populations coexist within the TME. Based on the spatial distribution of CD8^+^ T lymphocytes in the TME, solid tumors are generally classified into highly **inflamed**- “**hot**” and non-inflamed- “**cold**”. “Cold” tumors can be further subdivided into **immune desert** or **immune excluded** (Figure 2A). In “hot” tumors, T cells are present but inactive or exhausted. Immune desert tumors lack T cell penetration while immune excluded have the T cells accumulated at the invasive margins and absent from the tumor core 101, 102. Apart from TILs, additional features of the TME such as the expression of PD-1 receptor in T cells and PD-L1 in tumor cells and macrophages, degree of tumor mutational burden and the presence of a pre-existing antitumor immune response have been described as characteristics of “hot” tumors, related with good response to ICI. In fact, different studies in carcinomas demonstrate the role of TILs as biomarkers of response to ICI 103-105, ATC 106 and DC vaccination 107. However, such correlations have not been clearly established across sarcoma subtypes, instead more effort should be made to identify specific signatures of the TME among responders and achieve the level of evidence that defines them as “predictive biomarkers”.

For osteosarcoma, different studies have demonstrated that higher numbers of infiltrating CD8^+^ than Foxp3^+^ T cells separate survivors from non-survivors 108. On the contrary, the presence of macrophages in TME is rather more complex and depends on the shifts between the immunostimulatory M1 and the immune-suppressive M2 phenotype. In osteosarcoma, CD163^+^ M2 macrophages promote angiogenesis, and metastasis by mediating cancer cell extravasation and suppressing TIL homing 109-111. Sarcomas driven by reciprocal fusion oncoproteins, like Ewing sarcoma exhibit a “cold” microenvironment with low PD-L1 expression 112. TILs and DCs occur rarely whereas immunosuppressive cells of the myeloid linage like macrophages predominate at the tumor site and correlate with poorer overall survival 113.

With regards to soft tissue sarcoma, TIL abundance correlates with improved prognosis in high-grade undifferentiated pleomorphic sarcoma, gastrointestinal stromal tumor, cutaneous angiosarcoma, leiomyosarcoma and synovial sarcoma 114. Adding to this, the high expression of PD-1 and PD-L1 among lymphocytes reported across various subtypes was found to associate with higher tumor grading and lower survival 24, 25, 115. Macrophage infiltration is a common event in both copy number-driven and translocation-driven soft tissue sarcoma but not bone sarcoma subtypes. Except for embryonic rhabdomyosarcoma, high levels of CD163^+^ M2 macrophages have been associated with unfavorable outcome 116. In addition to macrophages, soft tissue sarcomas are dominated by B lymphocytes. A recent study showed that the high presence of B cells prior to neoadjuvant therapy associates with better survival and response 117, 118. Interestingly, other markers exhibiting a prognostic value as revealed by studies in carcinomas including tumor mutational burden and MSI, are dispensable for sarcomas. Particularly in soft tissue sarcomas, the tumor mutational burden is low and instability of microsatellites does not play a crucial role 119.

Thus, treatment decisions based on a single analyte most likely fail to capture the complete picture of the dynamic immune microenvironment leading to individuals undergoing unnecessary treatments. To this end, first efforts to develop immune-related signatures from responders including data from different clinical studies along with a scoring algorithm predicting response to ICI therapy have been already made while others are currently underway 120-123.

### Hypoxia

In agreement with other solid malignancies, sarcomas are defined by a leaky and fragile vascularture which impedes proper tissue oxygenation and nutrient delivery. To compensate for nutrient and oxygen scarcity, tumor cells trigger the HIF signaling pathway and consequent expression of pro-angiogenic proteins, such as vascular endothelial growth factor (VEGF), angiopoietin-1/-2, platelet-derived growth factor (PDGF) and basic fibroblast growth factor (bFGF). HIF-1α is the transcriptional activator of CTLA-4 in CD8^+^ T cells and PD-L1 (encoded by the CD274 gene) in tumor cells and various types of immune cells, such as MDSCs, macrophages, DCs, and bone marrow-derived macrophages 124. HIF-1α may also regulate the expression of PD-L1 through the activation of carbonic anhydrase 9. Carbonic anhydrase 9 causes tissue acidosis in the tumor stroma and the low pH, in turn, inhibits the cytotoxic function of CD8^+^ T cells and IFN production by Th1 cells. Hypoxia may also upregulate PD-1, CTLA-4 and TGF-β expression on the surface of T cells through the adenosine (Ado-A2aR) pathway which further promotes immune tolerance by diverting the cytokine and cellular profile of the TME away from cytotoxic T cell inflammation, leading to tumor progression and metastasis 125. Suppression of adenosine pathway using the CD73 inhibitor plus the ICI, durvalumab, is under evaluation in a phase 2 trial for the treatment of recurrent, refractory or metastatic sarcoma (NCT04668300). VEGF is considered the master regulator of tumor angiogenesis serving to increase endothelial cell proliferation, survival and migration, while promoting vessel permeability. In addition to their pro-angiogenic functions, VEGF and FGF drive endothelial cell anergy by downregulating the adhesion molecules on vessel walls required for T cell homing 126. HIF1α, VEGFs and VEGFRs are upregulated in at least 25% of sarcomas and their expression is linked with advanced tumor stage and poor prognosis 127, 128. Pazopanib is a VEGFR inhibitor approved for the treatment of chemotherapy-refractory sarcomas. Nevertheless, the responsiveness of sarcoma patients to pazopanib is limited to specific subtypes and does not always lead to improved clinical benefit in combination treatments, although preclinical data seemed promising. For example, pazopanib does not improve progression free survival of patients with advanced angiosarcomas when combined with carotuximab (TRC105), a TGFβ co-receptor and essential for angiogenesis 129.

In addition to the aberrant genomic instability caused by alterations in DNA repair pathways and release of free radicals, hypoxia promotes tumor aggressiveness via transcriptional regulation of downstream targets that sustain tumor aggressiveness and low immune cell infiltration 130. Recent preclinical findings indicate that transcriptomic response to hypoxia is well preserved across soft tissue sarcoma cell lines and agrees with the published gene signatures 130. The influence of hypoxia on many aspects of the TME pathophysiology explains why the first attempts of antiangiogenic treatment το completely shut down tumor vessels failed 131. Instead, lower doses of anti-VEGF therapy have been more successful in normalizing the tumor vasculature, increasing perfusion and enhancing T cell infiltration, suggesting that the judicious use of antiangiogenic agents is a promising strategy for cancer treatment 132. Nowadays, different clinical trials are investigating the combination of anti-angiogenic drugs with ICI in patients with different types of cancers, including alveolar soft part sarcoma, showing promising results 133-137.

The hypoxic stroma might be also exploited to activate prodrugs. For example, the hypoxia-activated prodrug TH-302 was found to significantly reduce hypoxia in a preclinical mouse prostate model while its combination with ICIs cured more than 80% of tumors by restoring T cell infiltration and reducing MDSCs 138. The apparent potential of TH-302 prodrug is curently evaluated in a phase 2 trial in combination with doxorubicin for patients with advanced soft tissue sarcoma (NCT01440088).

### Immunosuppressive extracellular matrix (ECM)

Another barrier of the TME that restricts infiltration of lymphocytes is the strong expression of mesenchymal and collagen barrier molecules in the ECM. In tumors, the ECM can undergo structural rearrangements to support tumor growth, including the production of collagen-driven fibrosis, a hallmark of many desmoplastic tumors 139. **CAFs** are the major source of collagen synthesis and most abundant cell population in the TME. They may coexist as a heterogeneous population characterized by distinct phenotypic markers, gene expression profile and functionality 140. Traditionally CAFs have been associated with aggressive behaviors and immune suppression. For instance, release of IL6 by CAFs promotes the differentiation of Tregs and IL17-producing T helper (Th17) cells 141, 142. Indeed, Th17 T cells possess both anti- and pro-tumor responses 143. On one side they recruit CD8^+^ T cells to the TME, while on the other, they release IL17 inducing the production of angiogenic factors from fibroblasts and cancer cells. Moreover, the direct interaction with T cells via the cell surface ligands displayed by CAFs prevents their trafficking within the TME. Adding to this, MHC-I-antigen presentation by CAFs combined with PD-L2 and FASL expression on the CAF cell surface, can result in killing of antigen-specific cytotoxic CD8^+^ T cells.

Unlike epithelial cancers, the current understanding of the origin and contribution of CAFs in sarcomas is very limited. Studies in Ewing sarcoma 144 and osteosarcoma 145 have demonstrated that extracellular vesicles and associated cargo secreted from tumor cells drive the transformation of normal fibroblasts into CAFs. On the contrary, rhabdomyosarcoma cells do not rely on CAFs to prime the ECM for local tumor expansion, but rather produce their own ECM with minimal involvement of CAFs 146. Other preclinical studies support that the transition of gastric resident fibroblasts to CAFs is mediated by TGFβ signaling, promoting cancer metastasis in gastrointestinal stromal tumor 147. CAFs may also promote immunosuppression via the CXCL12/CXCR4 and IL6/STAT3 signaling pathways. Osteosarcoma patients with high levels CXCL12 in CAFs have better overall survival 148, and CXCL12 targeting can enhance the sensitivity of these tumors to immunotherapy 149.

### Mesenchymal stem cells (MSCs)

While there is not a single cell origin ascribed to all sarcomas, increasing evidence suggests that MSCs are the sarcoma-initiating cells. MSCs are multipotent stem cells that differentiate towards diverse cell types including adipocytes, osteocytes, neural cells, fibroblast, chondrocytes and skeletal myoblasts. Several studies suggest that sarcomas arise from the malignant transformation of primitive MSCs or progenitor cells and many share a similar gene signature with the differentiated state of MSCs, possibly explaining the heterogeneity of these cancers. Although MSCs correspond to a small fraction of cells within the TME, yet they have a critical role in shaping the TME by promoting stemness, epithelial to mesenchymal transition, enhancing aggressiveness and drug resistance. It is very likely that resident tumor MSCs are responsible for relapse and metastasis. In osteosarcoma, the acidic microenvironment activates MSCs by inducing clonogenicity and invasion 150. Such induction, triggers MSCs to undergo aerobic glycolysis (Warburg effect) and subsequent production of lactate, the main driver of tumor acidosis. Besides fueling the tumor, *in vitro* findings demonstrate that MSCs stimulate osteosarcoma cells to express pro-angiogenic factors that promote the formation endothelial capillaries. Of course, MSCs have distinct immunomodulatory properties. Various studies have reported that they may sustain tumor progression by inhibiting T cell proliferation and activation, suppressing the cytotoxic activity of NKs, redirecting macrophages towards the immunosuppressive M2 phenotype and promoting regulatory T cell differentiation. In addition, MSCs inhibit B cell proliferation and antibody production 151.

### Metabolic barriers

Stroma acidification is another essential aspect of sarcoma microenvironment impeding immunotherapy. The limited delivery of serum nutrients and hypoxia push cancer cells to adopt alternative metabolic routes to cope with the high energetic demand for proliferation and survival. The catabolism of glucose via aerobic glycolysis is one primary metabolic adaptation that cancer cells undertake. However, a significant fraction of pyruvate is converted into lactic acid and secreted from the cell. Accumulation of lactic acid and other metabolic waste products in the TME impairs the function of immune cells inhibiting T cell proliferation and IFNγ production and thus, compromising the effector function of CD8^+^ T cells and NK cells. Furthermore, lactic acid inhibits monocyte activation and DC differentiation, promotes M2-polarization via increased arginase and HIF1α stabilization and increases the number of MDSCs. Finally, it may enhance the survival of Tregs, given the ability of Tregs to metabolize oxidized exogenous lactate. Many pediatric tumors including osteosarcoma and Ewing sarcoma have upregulated glycolysis and lactic acid fermentation. In fact, the oncogenic fusion protein EWS/FLI1 present in 80% of Ewing sarcoma cases, is the key regulator of the aberrant glycolytic reprograming of cancer cells 152. Moreover, high glycolytic flux and upregulated expression of lactate dehydrogenase, the enzyme that catalyzes the conversion of lactate to pyruvate and back, was found to associate with doxorubicin resistance in chondrosarcoma cell lines 153. Acidification as a mechanism of chemoresistance was also confirmed in osteosarcoma and rhabdomyosarcoma cell lines, where doxorubicin and other weak base drugs are trapped inside the highly acidic lysosomes due to an aberrant ion pumping (ion trapping mechanism) and consequently cannot target cancer cells 154. Although lactate-responsive pathways may offer opportunities to increase the efficacy of immunotherapy across diverse tumor types, it is important to consider that each of these targets is TME-context and immune cell specific and therefore lead to contradictory effects on immune cell function. Accordingly, neutralization of low pH may have a meaningful impact on improving the efficacy and outcomes of anticancer immunotherapy.

Depending on the cell type and environmental conditions the products of glycolysis can be utilized to produce nucleotides or enter the mitochondrial tricarboxylic acid cycle (TCA) cycle which in turn will provide the metabolic intermediates required for biosynthesis of lipids and amino acids. However, decreased entry of glucose into the TCA cycle combined with the shuttling of intermediates into the biosynthetic pathways, imposes a need for TCA anaplerosis with an alternative carbon source other than glucose. The most common carbon source involved in TCA anaplerosis is the glutamine.

Unlike glucose, which is required for both cancer and immune cell growth, glutamine is differentially utilized by each of these populations. Interestingly, inflammatory anti-tumor immune cells like M1 macrophages have less dependency on glutamine metabolism compared to M2 which rely on glutaminolysis for expansion. Also, studies in undifferentiated pleomorphic sarcoma showed that these cancer cells largely rely on glutamine as a source of energy and biosynthetic anabolism. Targeting of glutamine metabolism has been recently explored in preclinical models of undifferentiated pleomorphic sarcoma and additional soft tissue sarcoma subtypes showing encouraging results 155.

In addition to glutamine, cancer cells consume large quantities of arginine and tryptophan amino acids to support their growth and promote immune tolerance. Arginine is a crucial conditional amino acid for both cancer and immune cells. Arginine metabolism relies on the activity of arginase and inducible nitric oxide synthase (iNOS). Lactic acid abundance in TME favors the catabolism of arginine in myeloid cells via the arginase over iNOS, resulting in increased secretion of tumor-supporting factors by TAMs. On the contrary, TAMs utilizing iNOS exhibit an M1 phenotype and their nitric oxide upregulates the expression of VCAM1 adhesion molecules and subsequent T cell extravasation and homing against tumors. Importantly, CD8^+^ T cells benefit from L-arginine uptake by enhancing survival, memory formation and anti-tumor efficacy. Different primary sarcomas like osteosarcoma, Ewing sarcoma and rhabdomyosarcoma were reported to have lost or lack the ability to synthesize arginine *de novo* (arginine auxotrophic) and highly depend on extracellular arginine in TME 156, 157. Preclinical studies on animal models have demonstrated that combination therapy with L-arginine and anti-PD-L1 antibody boosts immune response against osteosarcoma 158. It should be mentioned that depletion of arginine in sarcoma patients either alone (NCT03455140) or in combination with gemcitabine and decotaxel (NCT03449901) is already exploited in clinic showing positive results 159. Its synergistic effects with immunotherapy, though, are yet to be determined in the clinical setting.

As opposed to glutamine and arginine, tryptophan is an essential amino acid which must be taken from the diet. Tryptophan acts as a substrate for kynurenine pathway. In tumors, however, increased levels of indoleamine 2,3-dioxygenase (IDO) catabolic enzymes (e.g., IDO1) deplete tryptophan from TME and promote the production of immunosuppressive kynurenine metabolites. Increased IDO1 activity prevents activation of effector T cells, inhibits NK function and supports Treg differentiation and MDSCs infiltration. Despite clinical trials using IDO inhibitors have so far led to disappointing results, combinations with anti-PD-1 improved the objective response rates in melanoma patients. Given the fact that IDO1 is highly expressed on Ewing sarcoma cells and has been associated with worse outcome in osteosarcoma, among other cancers 160, 161, combining such IDO inhibitors with immunotherapy may provide significant therapeutic benefit for those patients. In the case of STS, preclinical and clinical application of IDO inhibitors did not confer any significant benefit to anti-PD-L1 treatment, which slightly improved survival compared to untreated control in a model of murine fibrosarcoma 162. These data may explain in part the clinical failure of PD-1 inhibition in selected soft tissue sarcomas and gastrointestinal stromal tumors as a consequence of immunosuppressive TME resulting from macrophage infiltration and IDO1 pathway activation 163.

## Strategies to overcome barriers to sarcoma immunotherapy

We have thus far discussed the current progress in immunotherapy for the treatment of soft tissue and bone sarcoma with emphasis on the barriers impeding its antitumor activities. It appears that the high metabolic rate of cancer cells in conjunction with the poor vascularization and the limited nutrient exchange lead to a fierce competition for resources. In a TME deprived from amino acids and glucose, T cells fail to substantially increase their nutrient or engage the appropriate metabolic pathway, required to mount proper immune responses. In an effort to overcome these challenges, research has in part focused on reprogramming the TME as a promising approach for increasing the efficacy of many therapeutic agents, ranging from standard chemotherapeutics to nanomedicine and immunotherapy modalities. TME can be reprogrammed (i.e., normalized) both at the vascular and stromal level, so that it morphologically and functionally resembles the non-malignant state of the tissue 164-166 (Figure 2B). As such, TME reprogramming aims to improve tumor perfusion and treat hypoxia by normalizing either the abnormal structure of the tumor vessels or tumor stromal components. Of note, a recent phase 1b/2 trial (ImmunoSarc) indicated that inhibition of angiogenesis via sunitinib plus nivolumab is an active regimen with manageable toxicity in the treatment of selected patients with advanced soft tissue sarcoma with almost half of patients being free of progression at 6 months 167. Furthermore, preclinical studies on murine osteosarcoma models demonstrated that combination of sunitinib with PD-L1 blockade reduced the expression of PD-L1 by suppressing STAT3 activation, and thus inhibiting lung metastases, tumor growth which in turn improved survival 168.

### Stromal normalization/reprogramming

Nevertheless, application of antiangiogenic drugs does not impact perfusion of tumors with extremely compressed vessels, such as many sarcoma and pancreatic adenocarcinoma types, consistent with poor clinical response 169, 170. Vessel compression is independent from changes occurring in angiogenesis. It arises, though, from the excessive accumulation of mechanical forces generated by the rapid proliferation of tumor cells and CAFs within the confined space of the host tissue, a condition known as solid stress 171. Solid stress can be stored in ECM and the surrounding host tissue structural components and then transmitted to tumor vasculature 172. Accordingly, tumor stroma normalization/reprogramming strategies focus on targeting these ECM barriers, such as collagen, and the immediate environment allowing vessel decompression, reduction of tissue stiffness and improved intratumoral penetration of drugs and immune cells 171, 173. It is worth mentioning that depletion rather than reprogramming of ECM components besides contributing to stress alleviation it may increase the risk for disease progression 174.

Molecules with tissue reprogramming capabilities are known as “**mechanotherapeutics**” 175. Mechanotherapeutics refer to a subset of TME normalization therapies targeting the mechanical microenvironment (i.e., tumor stiffness and solid stress) in order to improve perfusion and alleviate hypoxia. Successful examples of mechanotherapeutics include common hypertensive drugs (e.g., losartan 176, 177), antihistamines (e.g., tranilast 178, 179), anti-diabetic drugs (e.g., metformin 180), anti-inflammatory drugs (e.g., dexamethasone 181, pirfenidone 182), endothelin receptor antagonists (e.g., bosentan 183), antifibrotic agents (e.g., vitamin D receptor agonists 184, pentoxifylline 185, relaxin 186, 187). In line with these studies, our recent findings in preclinical models of fibrosarcoma and osteosarcoma indicate that the antihistamine drug ketotifen not only inhibits ECM formation but also potentiates anti-PD-L1 immunotherapy by reverting the immunosuppressive TME and increasing overall survival. Significantly, combination therapy with anthracycline drugs (doxorubicin or epirubicin) show therapeutic superiority as opposed to anthracycline-anti-PD-L1 or anthracycline-ketotifen treatment, offering a durable remission and immunological memory. These therapeutic effects correlate well with a reduction in tumor stiffness and increase in vascular perfusion, suggesting that TME priming with such mechanotherapeutics is a prerequisite to creating favorable immunogenic conditions capable of eliminating the entirety of tumor and thus, providing a significant rational for clinical translation 188, 189.

Another mechanotherapeutic approach holding great promise in potentiating immunotherapy is the targeting of TGFβ signaling pathway. TGFβ signaling is abnormally upregulated in most cancers and widely associated with tumor growth and progression. TGFβ is expressed by cancer cells, DCs, macrophages, CAFs and immature myeloid cells, indicating functional divergence. Its role in CAFs has been linked to immunosuppressive responses via attenuation of tumor response to PD-L1 blockade and contributing to T cell exclusion 190 and by driving immune evasion 191, 192. Inhibition of TGFβ in mouse tumor models has been shown to trigger potent T cell responses 191, upregulate the expression VCAM-1 and ICAM-1 adhesion molecules 193 and promote the abscopal effect of radiotherapy 194. Specific downstream targets of TGFβ that contribute to this effect include NOX4 195 and CXCL12/CXCR4 pathway 196. Clinical exploitation of mechanotherapeutics in sarcoma immunotherapy has lagged behind that of other solid malignancies. Nonetheless, emerging preclinical data support that genetic ablation of TGFβ signaling specifically in NKp46^+^ cells could decrease the frequencies of intILC1 and ILC1 populations, which have been associated with immune suppression, in fibrosarcoma TME 197. In line with these results, pharmacological blocking of TGFβ receptor could restore immune suppression induced by regulatory B-T cell axis and decrease tumor burden in murine fibrosarcoma 198. A different study on osteosarcoma reported that vaccination with DC exposed to cryotreated tumor lysates combined with anti-TGFβ antibody increased CTLs and reduced regulatory T lymphocytes in the metastatic lesion mediating inhibition of metastatic growth 87. Accordingly, combining TGFβ blockade with ICI and other immunotherapy modalities is an attractive strategy to induce complete and durable responses in otherwise unresponsive soft tissue and bone sarcoma tumors.

### Potential of Nanomedicine to treat sarcoma and remodel the TME

Nanomedicine has improved therapeutic index and quality of life of sarcoma patients. DaunoXome^®^ and Doxil^®^ - liposomal formulations of daunorubicin and doxorubicin, respectively - were the first nanomedicines to be approved for the treatment of HIV-related Kaposi's sarcoma 199, 200. Later advances in nanotechnology led to the development of nab-paclitaxel (Abraxane^®^), a nanoparticle albumin-bound paclitaxel, which is a new type of taxane antineoplastic drug and has been indicated for the treatment of metastatic breast cancer, non-small cell lung cancer and metastatic pancreatic cancer 201. Evidence shows that nab-paclitaxel has promising effects in the treatment of angiosarcoma, epithelioid sarcoma, leiomyosarcoma, and other subtypes of soft tissue sarcomas 202 and can be safely combined with gemcitabine or receptor tyrosine inhibitors for an improved curative effect 203, 204. On the other hand, nanomedicine-immunotherapy combinations remain poorly investigated in sarcomas. To date, the safety and tolerability of nivolumab plus albumin-bound rapamycin (ABI-009) is evaluated in a phase 1/2 trial for advanced Ewing sarcoma and epithelioid sarcoma among other solid malignancies (NCT03190174). Irinotecan (NCT02013336), vincristine (NCT00038207) and epirubicin (NC6300, NCT03168061) encapsulated nanoparticles, are under clinical evaluation for the treatment of pediatric sarcomas and advanced solid tumors or advanced, metastatic, or unresectable soft tissue sarcoma, respectively. As therapeutic carriers, they have many advantages over conventional drug administration, including improved pharmacokinetic properties, prolonged circulating half-lives and sustained and controlled load release at the tumor site. The preferential accumulation at the tumor site is mediated through the enhanced permeability and retention (EPR) effect, which has been the key rational for the development of nanoparticle-based delivery systems 205-207. However, EPR heterogeneity between patients and tumor types may pose a barrier itself and hinder efficacy of nanomedicines. Furthermore, the enhanced permeability of tumor vessels that can cause an excessive fluid loss to the interstitial space and the impaired lymphatic drainage elevate the interstitial fluid pressure. As a result, interstitial fluid pressure becomes comparable to the microvascular pressure and diffusion becomes the main mechanism of nanoparticle transport across the tumor vessel walls, which does not favor the delivery of large therapeutic agents 208-210.

An emerging strategy to optimize the benefits of the EPR effect is by normalizing the TME, which results in reduction of interstitial fluid pressure without affecting tumor vessel hyperpermeability. With respect to this, we have shown that administration of nanomedicines in a metronomic fashion, defined as the frequent, low dose administration compared to the maximum tolerated dose at long time intervals, can normalize the TME. Although not clinically approved yet, our preclinical findings demonstrate that the metronomic administration of Doxil^®^ in mice bearing fibrosarcoma tumors yields improved anti-tumor effects than the conventional maximum tolerated dose schedule 211. In addition to its cytotoxic function, metronomic Doxil^®^ normalizes the TME, making the tumors softer, increasing perfusion and reducing interstitial fluid pressure and thus, overcoming the patho-physiological barriers to nanoparticle delivery. As a result of these normalization effects, combination of ICI with Doxil^®^ can revert immunosuppression and improve ICI efficacy 211.

Nanoparticle formulations could be further employed to normalize the TME by encapsulation of mechanotherapeutic agents that preferentially target the tumor. In this regard, nanomedicines designed to simultaneously target components of the TME and kill cancer cells can be used to broaden the therapeutic window of anticancer drugs. The development of drug delivery systems incorporating angiotensin receptor blockers as mechanotherapeutics conjugated to polymers that can be selectively degraded in the TME upon exposure to low pH is such an example 212, 213. Micellar formulations encapsulating the antihistamine tranilast have been also recently developed 214.

### Imagining techniques for monitoring TME burden

Several non-invasive imaging modalities can be employed for monitoring intratumoral drug distribution and efficacy, such as ultrasound imaging, magnetic resonance imaging (MRI), computed tomography (CT), Positron Emission Tomography (PET), single photon emission computed tomography (SPECT) and optical imaging 215. CT, MRI and ultrasound techniques allow the cross-sectional 3D visualization of the tissue and are frequently employed in the clinic to provide anatomical information to assist tumor staging and therapy monitoring. Furthermore, many of these imaging modalities can be used to image components of the TME related to the barriers of drug delivery, including tumor perfusion, hypoxia and vascular permeability. Specifically, PET allows for the use of specific radiotracers for measuring hypoxia, proliferation and angiogenesis, whereas contrast-enhanced CT and ultrasound methods have been employed to quantify tumor perfusion and the efficacy of the tumor micro-vasculature to effectively deliver drugs 216-219. In addition, MR and ultrasound can provide information of the stiffness of the tissue (i.e., MR elastography and shear wave elastography), which is directly related to tumor perfusion, and MRI can further measure the permeability of the tumor vessels 183, 214, 220. Given that tumor hypo-perfusion is a major barrier to the effective delivery of drugs and that normalization therapeutic strategies have been developed to restore the TME and thus, improve perfusion and drug efficacy, these imaging modalities can be employed to guide and monitor normalization treatments to optimize delivery of medicines, including immunotherapeutics 183, 214.

### Nano-immunotherapy reinforces the cancer immunity cycle

**Figure 3:**
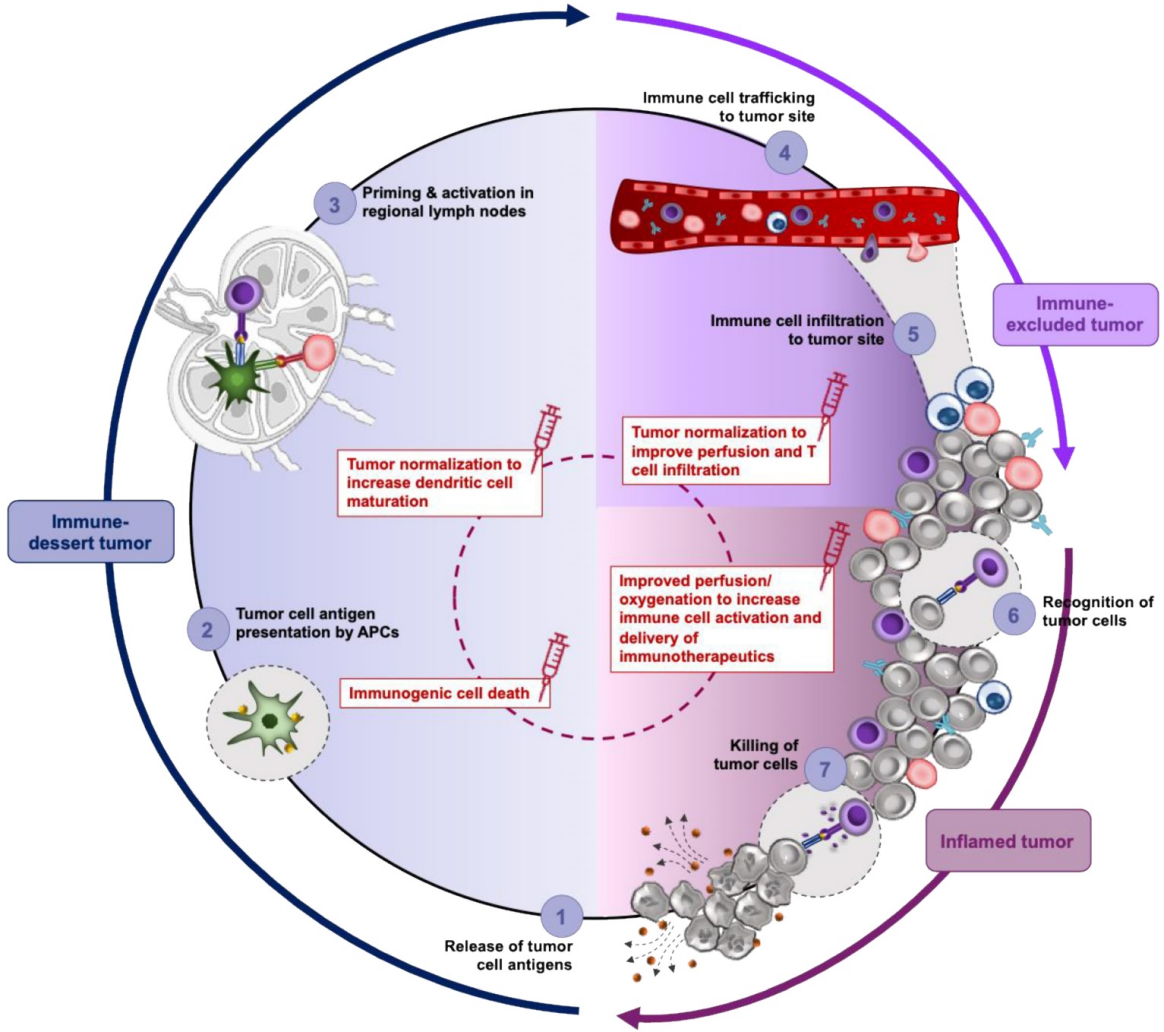
**TME normalization strategies affect immune phenotype and responsiveness to treatment**. In the immune desert phenotype (blue shading) the lack CTL in tumor parenchyma permits cancer cells to grow uninterruptedly favoring immunological ignorance (a lack of antigens and/or their presentation- step 1 and 2), tolerance (a lack of response to antigen presentation) and a lack of T cell priming (step 3). Accordingly, immune desert tumors are the least responsive to ICI. Under such conditions, vascular normalization improves the delivery of nanomedicine and increases immunogenic cell death and thereby release of tumor cell antigens and promotes antigen presentation through DC maturation. In tumors of the immune excluded phenotype (purple shading), T cells fail to penetrate tumor bed and are limited to the periphery. Penetration of T cells is primarily impeded by immature or compressed vessel and density of extravascular matrix and CAF-induced fibrosis. In addition, angiogenic signaling dysregulates the expression of adhesion molecules on the vessel wall, thereby reducing the extent of leukocyte binding and limiting their flux into tumors while hypoxia contributes to the establishment of immunosuppressive T cells like Tregs. Thus, TME normalization in immune excluded phenotypes improves tumor oxygenation, delivery drugs and makes the tumor stroma accessible to T cells (step 4 and 5). The immune inflamed TME (pink shading) is infiltrated by immune cells which have reduced antitumor activity due to various inhibitory factors, which are often induced by hypoxia (step 6 and 7). This phenotype has the most potential for sensitivity to ICI. Normalization strategies targeting VEGF signaling can be employed to restrict the recruitment of immunosuppressive immune cells like M2-like macrophages, Tregs and MDSC, while targeting of hypoxia will suppress immune checkpoint signaling allowing cancer cells to be recognized and killed by CTLs (step 6 and 7). Thus, selecting the type of normalization strategy (center, dashed red circle) based on the immunological properties of tumor can specifically enhance each step of cancer immunity cycle allowing its continuity.

Combining nanomedicine with immunotherapy aims to reinforce key steps of the cancer immunity cycle (Figure 3 and Refs 221-223 for a detailed review of this topic). Firstly, cancer nanomedicines can be utilized to deliver cytotoxic chemotherapy agents capable of inducing the release of tumor antigens and eliciting immunogenic cell death (step 1, Figure 3). The clinical potential of combining Abraxane^®^ with the PD-1 inhibitor atezolizumab has already been demonstrated in a phase 3 clinical trial for the treatment of triple negative breast cancer and in a retrospective study of soft tissue sarcoma, with angiosarcoma patients reporting a significantly prolonged free survival compared to other subtypes 224. Doxil^®^, similarly to Abraxane^®^, induces immunogenic cell death by promoting immune cell infiltration and reverting tumor immunosuppression 225. A second strategy to integrate nanomedicine in the cancer-immunity cycle is by potentiating the antigen uptake, processing and presentation with the use of adjuvants (step 2-3, Figure 3). Targeted delivery of Toll-like receptor agonists to antigen presenting cells in secondary lymphoid organs can boost their anti-tumor immune responses while minimizing severe side effects associated with adjuvant therapy. Adding to this, nanocarriers can be utilized to deliver cytokines (e.g., IL2) to stimulate and expand T cell population 221. Finally, nanomedicine can be utilized as a co-treatment to prime the TME for improved efficacy of immunotherapy modalities. As discussed above, such nanomedicines may incorporate mechanotherapeutic agents and their added value in this regard is related to their ability to normalize the TME, revert the immunosuppressive phenotype of TAMs and promote immune activation. In a prospective randomized phase 3 trial for the treatment of osteosarcoma, the combination of the immune modulator, liposomal muramyl tripeptide phosphatidylethanolamine, with a three-drug chemotherapy regimen (doxorubicin, cisplatin, methotrexane) demonstrated a trend of improved overall survival for the patients who received the liposomal nanoparticle in addition to chemotherapy regimen 226. However, the study was not adequately powered to make firm conclusions.

## Adverse effects of immunotherapy

Immunotherapy advances, despite their promise, are often associated with immune related adverse events (irAEs), as a consequence of the nonspecific immune activation in the human body. irAEs differ from the classical chemotherapy-induced toxicities and may occur in almost any organ such as colon, muscle, lungs, liver and thyroid. Their frequency depends on the dosage, regimens and exposure time but also on patient's intrinsic factors. A few studies have specifically assessed the safety of ICI in patients with recurrent or therapy refractory sarcomas. A phase 1 dose escalation study (NCT01445379), investigating the tolerance and toxicity profile of ipilimumab monotherapy in children and young adults indicated that the occurrence of high grade irAEs associates with better response to CTLA-4 inhibition, although a proportional increase in the frequency of irAEs was observed with an increase in dose level. A different study assessing the safety of nivolumab as monotherapy (ADVL1412, NCT02304458) showed that administration of 3 mg/kg every 14 days is well tolerated in children with Ewing, rhabdomyosarcoma, and osteosarcoma subtypes and the most common irAEs were: increased lipase levels and cardiac and pleural effusion. In the SARC028 (NCT02301039) study, the most frequent grade 3 or worse adverse effect reported in both bone and soft tissue sarcoma groups was anaemia, followed by decreased lymphocyte count and prolonged activated partial thromboplastin time. Also, some patients of the bone sarcoma group had decreased platelet count. Notably, none of these treatment-related serious adverse events were fatal. Alliance (A091401, NCT02500797) 27 is another study investigating tolerability of nivolumab with or without ipilimumab. The dose and schedule for the combination tested (3 mg/kg nivolumab plus 1 mg/kg ipilimumab) had acceptable toxicity, with 14% of patients having grade 3 or 4 treatment-related adverse events. Similar to other studies, anaemia and decreased lymphocyte count were the most frequent adverse effects reported among patients. Additional irAEs reported were dehydration, increased lipase, pain, pleural effusion, respiratory failure, secondary benign neoplasm, and urinary tract obstruction.

In general, the incidence of fatal irAEs for ICI not only for sarcomas but among all tumor types ranges between 0.3% and 1.3%, which is lower compared to conventional treatments. A meta-analysis by Wang et al. in 2018 227 indicated colitis for CTLA-4 inhibitors (70%, 135/193 deaths), pneumonitis (35%, 115/333 deaths) for PD-1 or PD-L1 inhibitors, and colitis (37%, 32/87) for the combination PD-1 and CTLA-4 inhibition as the most frequent causes of death as an irAE. Less life-threatening irAEs including rash, pruritus and vitiligo have been reported in more than one-third of the patients 227. However, this study failed to provide data about the incidences of low-grade and high-grade adverse events and included only a limited number of sarcoma patients and thus, may did not recapitulate the extend of fatal ICI-associated toxic effects. Accordingly, a more comprehensive analysis aiming to compare organ-specific irAEs of ICI monotherapy versus combination among patients of the same cancer is essential for clinicians to balance the benefits and risks of ICI during treatment 228.

## Conclusion

Undoubtedly, the limited number of patients, the high interpatient heterogeneity and the lack of specific markers expressed by most sarcoma cells contribute to the limited advances in the field of immunotherapy. Consequently, it is not surprising that the clinical success of ICI results in sporadic therapeutic responses in sarcoma. Combination treatments have been employed instead to overcome these challenges. As described earlier, several preclinical studies indicate that the future of immunotherapy relies on the combination with conventional chemotherapeutics and radiotherapeutics or the combination of two or more different immunotherapy modalities. Although such combinatorial treatment schemes manage to accentuate the effect in most sarcoma subtypes, the reported influence on complete response and progression free survival remains minimal. Given that ICI normalization of the TME alleviates hypoxia towards increasing efficacy of immune checkpoint inhibition, it may worth exploring the impact of “mechanotherapeutics” as means to increase the quality and magnitude of immunotherapy modalities. In line with this, accumulating evidence indicates that targeting of fibrosis by suppressing CAFs not only influences the *de novo* immune responses but dictates the success of immunotherapies as well. Supported by the stiff ECM, CAFs interact with cancer cells to convey metabolic signals required for cancer cell glycolysis, increased catabolic activity and autophagy. Meanwhile, the rapid consumption of available nutrients by cancer cells and secretion of lactate, create regions of hypoxia and high acidity, which further hinder immune cell function. Importantly, T cells found in hypoxic areas of inflamed sarcoma tumors exhibit a profound mitochondrial dysfunction and lack of cytokine production until after checkpoint blockade, suggesting that antigen recognition and infiltration into tumors alone are insufficient for an antitumor response and that the metabolic TME can directly suppress T cells. However, direct targeting of the hypoxia-sensing pathway, HIF, is not immune specific and thus, it may have conflicting effects on the function of CTLs and anti-tumor therapies. Likewise, metabolic pathways with distinct dependencies on cancer and pro-inflammatory immune cells and macrophages must be exploited to achieve a multi-pronged approach for cancer therapy. While “mechanotherapeutics” alone do not possess any antitumor effects, their application lies on targeting the tumor-induced vasculature and ECM that hamper T cell migration and effector function of resident cells, as such allowing efficient priming of TME for an effective antitumor immune response. Thus, their role although complementary is rather crucial for T cell accumulation, promoting antigen presentation and activation of T cells and suppressing the immune tolerance. It is expected that upcoming studies employing TME normalization strategies will be focused on the stratification and selection of patients based on the immune phenotype (i.e., immune dessert, immune excluded or immune inflamed) (Figure 3) and metabolic adaptations. These immune phenotypes reflect tumors at different phases of the seven-step cancer-immunity cycle which must be completed repeatedly in order for immunotherapies to be effective. TME normalization can enhance each step of the cancer-immunity cycle and promote its perpetuation.

With respect to future directions, convergence of artificial intelligence and precision medicine offers a breakthrough in biomarker research and disease diagnosis. Indeed, in sarcomas there is not “one-size fits all” molecular target and each subtype needs to be studied independently. This underlines the need for elaborating new minimally invasive techniques to monitor patient progress at a given time like high-throughput molecular profiling. An ongoing clinical trial, MULTISARC (NCT03784014), assesses the feasibility of next generation sequencing exome in metastatic soft tissue sarcoma patients to identify actionable mutations. Under clinical investigation is also the development of conserved transcription signatures spanning sarcoma subtypes including the Complexity Index in Sarcomas (CINSARC), hypoxia associate signature and genomic grade index 229. The next step will be to integrate the information obtained from clinical cohorts and preclinical studies in sarcoma cell lines, patient derived samples and tumor models to improve prediction of response to therapy. Assessment of circulating tumor cells, cell-free circulating DNA, tumor derived extracellular vesicles and metabolomic profiling could complement the immunohistochemistry data and assist the genomically guided lines of treatment. Precision medicine relies on the ability to integrate high throughput molecular and computational data to increase the accuracy of diagnosis, prognosis and to identify the most effective therapy.

## Supplementary Material

Supplementary tables.

## Figures and Tables

**Table 1:** Selected completed immunotherapy clinical trials for Soft tissue sarcomas and Bone sarcomas.

Immunotherapy Intervention		Study	Disease	Response rate	Survival
**ICIs**	Pembrolizumab	Phase 2 (NCT02301039) 26	Advanced STS and BS	-18% in STS; 40% in UPS, 20% in LPS, 10% in SS, 0% in LMS -5% in BS; 5% in OS, 20% in CHS, 0% in EwS	mPFS: 19.1 months in STS mPFS: 17.8 months in BS
	Pembrolizumab + cyclophosphamide	Phase 2 (NCT02406781) 163	Advanced STS	-0% in LMS -0% in UPS -14.3% in other -11.1% in GIST	mPFS: 1.4 months in LMS, UPS, other sarcomas and GIST mOS: 9.2 months in LMS, 5.6 months in UPS, 7.1 months in other sarcomas -not reached in GIST
	Nivolumab +/- Ipilimumab	Phase 2 (NCT02500797) 27	Metastatic STS	-Nivolumab group: 8% (ASPS, non-uterine LMS, sarcoma NOS) -Nivolumab + Ipilimumab group: 15% (uterine LMS, non-uterine LMS, UPS/MFH, angiosarcoma, myxofibrosarcoma)	-Nivolumab: mPFS: 1.7 months mOS: 10.7 months -Nivolumab + Ipilimumab: mPFS: 4.1 months mOS: 14.3 months
	T-VEC + pembrolizumab	Phase 2 (NCT03069378) 54	Advanced or metastatic sarcoma (LMS, angiosarcomas, UPS, undifferentiated or unclassified sarcoma, other histologic subtypes)	-PR: 35% (7 patients) -SD: 35% (7 patients) -PD: 30% (6 patients)	mPFS: 17.1 weeks
	Nivolumab + sunitinib	Phase 1b/2 (NCT03277924) 167	Advanced STS	-ORR: 21%	mPFS: 5.6 months for central and 6 months for local assessment mOS: 24 months
**ACT**	HER2-specific CAR T cell	Phase 1/2 (NCT00902044) 41	Refractory or recurrent metastatic HER2 positive sarcomas	-PR: 5.8% (1 patient) -SD: 23.5% (4 patients) -PD: 70.6% (12 patients)	mPFS: 10.1 mOS: 10.3 months
	NY-ESO-1 SPEAR T cells	Phase 1/2 (NCT01343043) 39	ESO-1 positive SS	-CR: 2.4% (1 patient) -PR: 33.4% (14 patients) -SD: 57.1% (24 patients) -PD: 7.1% (3 patients)	
**Cancer Vaccines**	autologous lymphocyte infusion plus KLH pulsed dendritic cell vaccine +/- rhIL7	Phase 2 (NCT00923351) 47	metastatic or recurrent EwS, RMS, DSRCT, SS and undifferentiated sarcoma		-5-year OS: 63% for EwS/RMS and 0% for other sarcomas -5-year PFS: 40% for EwS/RMS and 0% for other sarcomas
	Autologous tumor lysate pulsed DCs	Phase 1/2 49	Metastatic or recurrent BS and STS	-PR: 2.9% (1 patient) -SD: 17.1% (6 patients) -PD: 80% (28 patients)	-3-year OS rate: 42.3% -3-year PFS rate: 2.9%
	CMB305 vaccine (NY-ESO-1 expressing lentiviral vector, and recombinant adjuvanted NY-ESO-1 protein)	Phase 1b 230	NY-ESO-1 positive locally advanced, relapsed, or metastatic sarcomas	-DCR: 61.9%	OS: 26.2 months

STS, soft tissue sarcoma; BS, bone sarcoma; LPS, liposarcoma; RMS, rhabdomyosarcoma; LMS, leiomyosarcoma; SS, synovial sarcoma; UPS, undifferentiated pleomorphic sarcoma; GIST, Gastrointestinal stromal tumor; ASPS, alveolar soft part sarcoma; MFH, malignant fibrous histiocytoma; OS, osterosarcoma; CHS, chondrosarcoma; EwS, Ewing sarcoma; DSRCT, desmoplastic small round cell tumors; ICIs, immune checkpoint inhibitors; ACT, adoptive cell therapy; CR, complete response; PR, partial response; SD, stable disease; PD, progressive disease; ORR, objective response rate; DCR, disease control rate; mPFS, median progression-free survival; mOS, median overall survival

**Table 2:** Preclinical Studies for Soft Tissue Sarcoma Immunotherapy.

Intervention	Monotherapy	ICIs	ACT	Oncolytic viruses	Cancer Vaccines	*In situ* vaccines	Immunotherapy + iSVs + CT/RT
**ICIs**	FB 231 DDLPS 77						FB 188, 189
**ACT**	FB 232 RMS 65 UPS 68 24JK-HER-2 233						
**Oncolytic viruses**	LMS 67	LPS 78	FB 98				
**Cancer Vaccines**	FB 234-242	FB 243, 244					
***In situ* vaccines**	FB 197, 198, 245-251 SS 79 LMS 66 F244 MCA 252	FB 55, 56, 69, 162, 253-255 UPS 69 1956 sarcoma 256	FB 257 RMS 60, 61	RMS 64	FB 58, 258 RMS 62, 63 MCA304 259	FB 260-263	
**CT/RT**						FB 59, 264-266	FB 267, 268

FB, fibrosarcoma; DDLPS, dedifferentiated liposarcoma; LPS, liposarcoma; RMS, rhabdomyosarcoma; LMS, leiomyosarcoma; SS, synovial sarcoma; UPS, undifferentiated pleomorphic sarcoma; CT, chemotherapy; RT, radiotherapy; ICIs, immune checkpoint inhibitors; ACT, adoptive cell therapy; iSVs, *in situ* vaccines

**Table 3:** Preclinical Studies for Bone Sarcoma Immunotherapy.

Intervention	Monotherapy	ICIs	ACT	Oncolytic viruses	Cancer Vaccines	iSVs	Immunotherapy + iSVs + CT/RT
**ICIs**	OS 269-272						OS 188, 189
**ACT**	OS 273-278 EwS 274, 279-284	OS 91					OS 81
**Oncolytic viruses**	OS 285	OS 286	EwS 98				
**Cancer vaccines**	OS 241, 287-291	OS 90, 292					
**iSVs**	OS 293-313	OS 80, 314, 315	OS 82-85 EwS 85, 316 CHS 100		OS 86, 87		
**CT/RT/ surgery**		OS 168, 317, 318				OS 88, 89, 319, 320	

OS, osteosarcoma; EwS, Ewing sarcoma; CHS, chondrosarcoma; CT, chemotherapy; RT, radiotherapy; ICIs, immune checkpoint inhibitors; ACT, adoptive cell therapy; iSVs, *in situ* vaccines

## References

[1] Choi JH, Ro JY (2021). The 2020 WHO classification of tumors of soft tissue: selected changes and new entities. Adv Anat Pathol.

[2] WHO Classification of Tumours Editorial Board (2020). WHO classification of tumours: soft tissue and bone tumours: 5th ed. Lyon: International Agency for Research on Cancer.

[3] Gronchi A, Miah AB, Dei Tos AP (2021). Soft tissue and visceral sarcomas: ESMO-EURACAN-GENTURIS Clinical Practice Guidelines for diagnosis, treatment and follow-up. Ann Oncol.

[4] Strauss SJ, Frezza AM, Abecassis N (2021). Bone sarcomas: ESMO-EURACAN-GENTURIS-ERN PaedCan Clinical Practice Guideline for diagnosis, treatment and follow-up. Ann Oncol.

[5] García-del-Muro X, López-Pousa A, Maurel J (2011). Randomized phase II study comparing gemcitabine plus dacarbazine versus dacarbazine alone in patients with previously treated soft tissue sarcoma: a Spanish Group for Research on Sarcomas study. J Clin Oncol.

[6] Maki RG, Wathen JK, Patel SR (2007). Randomized phase II study of gemcitabine and docetaxel compared with gemcitabine alone in patients with metastatic soft tissue sarcomas: results of sarcoma alliance for research through collaboration study 002 [corrected]. J Clin Oncol.

[7] Martin-Liberal J, Alam S, Constantinidou A (2013). Clinical activity and tolerability of a 14-day infusional Ifosfamide schedule in soft-tissue sarcoma. Sarcoma.

[8] Demetri GD, Von Mehren M, Jones RL (2016). Efficacy and safety of trabectedin or dacarbazine for metastatic liposarcoma or leiomyosarcoma after failure of conventional chemotherapy: results of a phase III randomized multicenter clinical trial. J Clin Oncol.

[9] Van der Graaf, W T, Blay JY, Chawla SP (2012). EORTC Soft Tissue and Bone Sarcoma Group; PALETTE study group. Pazopanib for metastatic soft-tissue sarcoma (PALETTE): a randomised, double-blind, placebo-controlled phase 3 trial. Lancet.

[10] Demetri GD, Schöffski P, Grignani G (2017). Activity of eribulin in patients with advanced liposarcoma demonstrated in a subgroup analysis from a randomized phase III study of eribulin versus dacarbazine. J Clin Oncol.

[11] Miller KD, Fidler-Benaoudia M, Keegan TH (2020). Cancer statistics for adolescents and young adults, 2020. CA Cancer J Clin.

[12] Rutkowski P, Ługowska I (2014). Follow-up in soft tissue sarcomas. Memo.

[13] He X, Xu C (2020). Immune checkpoint signaling and cancer immunotherapy. Cell Res.

[14] Postow MA, Chesney J, Pavlick AC (2015). Nivolumab and ipilimumab versus ipilimumab in untreated melanoma. N Engl J Med.

[15] Eggermont AM, Chiarion-Sileni V, Grob J (2016). Prolonged survival in stage III melanoma with ipilimumab adjuvant therapy. N Engl J Med.

[16] Tawbi HA, Forsyth PA, Algazi A (2018). Combined nivolumab and ipilimumab in melanoma metastatic to the brain. N Engl J Med.

[17] Schmid P, Adams S, Rugo HS (2018). Atezolizumab and nab-paclitaxel in advanced triple-negative breast cancer. N Engl J Med.

[18] Socinski MA, Jotte RM, Cappuzzo F (2018). Atezolizumab for first-line treatment of metastatic nonsquamous NSCLC. N Engl J Med.

[19] Vellanki PJ, Mulkey F, Jaigirdar AA (2021). FDA approval summary: nivolumab with ipilimumab and chemotherapy for metastatic non-small cell lung cancer, a collaborative project orbis review. Clin Cancer Res.

[20] Suzman DL, Agrawal S, Ning Y (2019). FDA approval summary: atezolizumab or pembrolizumab for the treatment of patients with advanced urothelial carcinoma ineligible for cisplatin-containing chemotherapy. Oncologist.

[21] Motzer RJ, Escudier B, McDermott DF (2015). Nivolumab versus everolimus in advanced renal-cell carcinoma. N Engl J Med.

[22] Weber JS, D'Angelo SP, Minor D (2015). Nivolumab versus chemotherapy in patients with advanced melanoma who progressed after anti-CTLA-4 treatment (CheckMate 037): a randomised, controlled, open-label, phase 3 trial. Lancet Oncol.

[23] Larkin J, Minor D, D'Angelo S (2018). Overall survival in patients with advanced melanoma who received nivolumab versus investigator's choice chemotherapy in CheckMate 037: a randomized, controlled, open-label phase III trial. J Clin Oncol.

[24] D'Angelo SP, Shoushtari AN, Agaram NP (2015). Prevalence of tumor-infiltrating lymphocytes and PD-L1 expression in the soft tissue sarcoma microenvironment. Hum Pathol.

[25] Kim JR, Moon YJ, Kwon KS (2013). Tumor infiltrating PD1-positive lymphocytes and the expression of PD-L1 predict poor prognosis of soft tissue sarcomas. PloS One.

[26] Tawbi HA, Burgess M, Bolejack V (2017). Pembrolizumab in advanced soft-tissue sarcoma and bone sarcoma (SARC028): a multicentre, two-cohort, single-arm, open-label, phase 2 trial. Lancet Oncol.

[27] D'Angelo SP, Mahoney MR, Van Tine BA (2018). Nivolumab with or without ipilimumab treatment for metastatic sarcoma (Alliance A091401): two open-label, non-comparative, randomised, phase 2 trials. Lancet Oncol.

[28] Liu Y, Sun Z (2021). Turning cold tumors into hot tumors by improving T-cell infiltration. Theranostics.

[29] Samstein RM, Lee C, Shoushtari AN (2019). Tumor mutational load predicts survival after immunotherapy across multiple cancer types. Nat Genet.

[30] Le DT, Durham JN, Smith KN (2017). Mismatch repair deficiency predicts response of solid tumors to PD-1 blockade. Science.

[31] Havel JJ, Chowell D, Chan TA (2019). The evolving landscape of biomarkers for checkpoint inhibitor immunotherapy. Nat Rev Cancer.

[32] Dudley JC, Lin M, Le DT (2016). Microsatellite instability as a biomarker for PD-1 blockade. Clin Cancer Res.

[33] Met Ö, Jensen KM, Chamberlain CA (2019). Principles of adoptive T cell therapy in cancer. Semin Immunopathol.

[34] Rohaan MW, Wilgenhof S, Haanen JB (2019). Adoptive cellular therapies: the current landscape. Virchows Arch.

[35] Lauss M, Donia M, Harbst K (2017). Mutational and putative neoantigen load predict clinical benefit of adoptive T cell therapy in melanoma. Nat Commun.

[36] Chandran SS, Somerville RP, Yang JC (2017). Treatment of metastatic uveal melanoma with adoptive transfer of tumour-infiltrating lymphocytes: a single-centre, two-stage, single-arm, phase 2 study. Lancet Oncol.

[37] Shi J, Li M, Yang R (2020). Tumor-infiltrating lymphocytes as a feasible adjuvant immunotherapy for osteosarcoma with a poor response to neoadjuvant chemotherapy. Immunotherapy.

[38] D'Angelo SP, Melchiori L, Merchant MS (2018). Antitumor activity associated with prolonged persistence of adoptively transferred NY-ESO-1 c259T cells in synovial sarcoma. Cancer Discov.

[39] Ramachandran I, Lowther DE, Dryer-Minnerly R (2019). Systemic and local immunity following adoptive transfer of NY-ESO-1 SPEAR T cells in synovial sarcoma. J Immunother Cancer.

[40] Umut Ö, Gottschlich A, Endres S (2021). CAR T cell therapy in solid tumors: a short review. Memo.

[41] Ahmed N, Brawley VS, Hegde M (2015). Human epidermal growth factor receptor 2 (HER2)-specific chimeric antigen receptor-modified T cells for the immunotherapy of HER2-positive sarcoma. J Clin Oncol.

[42] Chruściel E, Urban-Wójciuk Z, Arcimowicz Ł (2020). Adoptive cell therapy—harnessing antigen-specific T cells to target solid tumours. Cancers.

[43] Liu S, Galat V, Galat Y (2021). NK cell-based cancer immunotherapy: From basic biology to clinical development. J Hematol Oncol.

[44] Böttcher JP, Bonavita E, Chakravarty P (2018). NK cells stimulate recruitment of cDC1 into the tumor microenvironment promoting cancer immune control. Cell.

[45] Ruggeri L, Capanni M, Urbani E (2002). Effectiveness of donor natural killer cell alloreactivity in mismatched hematopoietic transplants. Science.

[46] Miller JS, Soignier Y, Panoskaltsis-Mortari A (2005). Successful adoptive transfer and *in vivo* expansion of human haploidentical NK cells in patients with cancer. Blood.

[47] Merchant MS, Bernstein D, Amoako M (2016). Adjuvant immunotherapy to improve outcome in high-risk pediatric sarcomas. Clin Cancer Res.

[48] Ghisoli M, Barve M, Mennel R (2016). Three-year follow up of GMCSF/bi-shRNAfurin DNA-transfected Autologous tumor immunotherapy (vigil) in metastatic advanced Ewing's sarcoma. Mol Ther.

[49] Miwa S, Nishida H, Tanzawa Y (2017). Phase 1/2 study of immunotherapy with dendritic cells pulsed with autologous tumor lysate in patients with refractory bone and soft tissue sarcoma. Cancer.

[50] Himoudi N, Wallace R, Parsley KL (2012). Lack of T-cell responses following autologous tumour lysate pulsed dendritic cell vaccination, in patients with relapsed osteosarcoma. Clin Transl Oncol.

[51] Saxena M, van der Burg, Sjoerd H, Melief CJ (2021). Therapeutic cancer vaccines. Nat Rev Cancer.

[52] Wang G, Kang X, Chen KS (2020). An engineered oncolytic virus expressing PD-L1 inhibitors activates tumor neoantigen-specific T cell responses. Nat Commun.

[53] Andtbacka RH, Kaufman HL, Collichio F (2015). Talimogene laherparepvec improves durable response rate in patients with advanced melanoma. J Clin Oncol.

[54] Kelly CM, Antonescu CR, Bowler T (2020). Objective response rate among patients with locally advanced or metastatic sarcoma treated with talimogene laherparepvec in combination with pembrolizumab: a phase 2 clinical trial. JAMA Oncol.

[55] Redmond WL, Linch SN, Kasiewicz MJ (2014). Combined targeting of costimulatory (OX40) and coinhibitory (CTLA-4) pathways elicits potent effector T cells capable of driving robust antitumor immunity. Cancer Immunol Res.

[56] Puca E, Probst P, Stringhini M (2020). The antibody-based delivery of interleukin-12 to solid tumors boosts NK and CD8 T cell activity and synergizes with immune checkpoint inhibitors. Int J Cancer.

[57] Wykosky J, Debinski W (2008). The EphA2 receptor and ephrinA1 ligand in solid tumors: function and therapeutic targeting. Mol Cancer Res.

[58] Rao A, Taylor JL, Chi-Sabins N (2012). Combination therapy with HSP90 inhibitor 17-DMAG reconditions the tumor microenvironment to improve recruitment of therapeutic T cells. Cancer Res.

[59] Yamazaki T, Hannani D, Poirier-Colame V (2014). Defective immunogenic cell death of HMGB1-deficient tumors: compensatory therapy with TLR4 agonists. Cell Death Differ.

[60] Vela M, Bueno D, González-Navarro P (2019). Anti-CXCR4 antibody combined with activated and expanded Natural Killer cells for sarcoma immunotherapy. Front Immunol.

[61] Simon-Keller K, Paschen A, Hombach AA (2013). Survivin blockade sensitizes rhabdomyosarcoma cells for lysis by fetal acetylcholine receptor-redirected T cells. Am J Pathol.

[62] Donahue RN, Duncan BB, Fry TJ (2014). A pan inhibitor of DASH family enzymes induces immunogenic modulation and sensitizes murine and human carcinoma cells to antigen-specific cytotoxic T lymphocyte killing: implications for combination therapy with cancer vaccines. Vaccine.

[63] Duncan BB, Highfill SL, Qin H (2013). A pan-inhibitor of DASH family enzymes induces immune-mediated regression of murine sarcoma and is a potent adjuvant to dendritic cell vaccination and adoptive T-cell therapy. J Immunother.

[64] Dobson CC, Naing T, Beug ST (2017). Oncolytic virus synergizes with Smac mimetic compounds to induce rhabdomyosarcoma cell death in a syngeneic murine model. Oncotarget.

[65] Merker M, Wagner J, Kreyenberg H (2020). ERBB2-CAR-engineered cytokine-induced killer cells exhibit both CAR-mediated and innate immunity against high-risk rhabdomyosarcoma. Front Immunol.

[66] Edris B, Weiskopf K, Volkmer AK (2012). Antibody therapy targeting the CD47 protein is effective in a model of aggressive metastatic leiomyosarcoma. PNAS.

[67] Bramante S, Koski A, Kipar A (2014). Serotype chimeric oncolytic adenovirus coding for GM-CSF for treatment of sarcoma in rodents and humans. Int J Cancer.

[68] Sangiolo D, Mesiano G, Gammaitoni L (2014). Cytokine-induced killer cells eradicate bone and soft-tissue sarcomas. Cancer Res.

[69] Devalaraja S, To TKJ, Folkert IW (2020). Tumor-derived retinoic acid regulates intratumoral monocyte differentiation to promote immune suppression. Cell.

[70] Erkelens MN, Mebius RE (2017). Retinoic acid and immune homeostasis: a balancing act. Trends Immunol.

[71] Jin C, Hong CY, Takei M (2010). All-trans retinoic acid inhibits the differentiation, maturation, and function of human monocyte-derived dendritic cells. Leuk Res.

[72] Hill JA, Hall JA, Sun C (2008). Retinoic acid enhances Foxp3 induction indirectly by relieving inhibition from CD4 CD44hi Cells. Immunity.

[73] Bhatt S, Qin J, Bennett C (2014). All-trans retinoic acid induces arginase-1 and inducible nitric oxide synthase-producing dendritic cells with T cell inhibitory function. J Immunol.

[74] Vellozo NS, Pereira-Marques ST, Cabral-Piccin MP (2017). All-trans retinoic acid promotes an M1-to M2-phenotype shift and inhibits macrophage-mediated immunity to Leishmania major. Front Immunol.

[75] Bhattacharya N, Yuan R, Prestwood TR (2016). Normalizing microbiota-induced retinoic acid deficiency stimulates protective CD8 T cell-mediated immunity in colorectal cancer. Immunity.

[76] Mohty M, Morbelli S, Isnardon D (2003). All-trans retinoic acid skews monocyte differentiation into interleukin-12-secreting dendritic-like cells. Br J Haematol.

[77] Choi B, Lee JS, Kim SJ (2020). Anti-tumor effects of anti-PD-1 antibody, pembrolizumab, in humanized NSG PDX mice xenografted with dedifferentiated liposarcoma. Cancer Lett.

[78] Smith HG, Mansfield D, Roulstone V (2019). PD-1 blockade following isolated limb perfusion with vaccinia virus prevents local and distant relapse of soft-tissue sarcoma. Clin Cancer Res.

[79] Li HK, Sugyo A, Tsuji AB (2018). α-particle therapy for synovial sarcoma in the mouse using an astatine-211-labeled antibody against frizzled homolog 10. Cancer Sci.

[80] Jiang K, Li J, Zhang J (2019). SDF-1/CXCR4 axis facilitates myeloid-derived suppressor cells accumulation in osteosarcoma microenvironment and blunts the response to anti-PD-1 therapy. Int Immunopharmacol.

[81] Fernandez L, Valentin J, Zalacain M (2015). Activated and expanded natural killer cells target osteosarcoma tumor initiating cells in an NKG2D-NKG2DL dependent manner. Cancer Lett.

[82] Kiany S, Gordon N (2016). Aerosol delivery of interleukin-2 in combination with adoptive transfer of natural killer cells for the treatment of lung metastasis: Methodology and effect. In: Somanchi SS, Ed. Natural Killer Cells: Methods and Protocols, Methods in Molecular Biology. New York: Humana Press Springer.

[83] Udagawa T, Narumi K, Goto N (2012). Syngeneic hematopoietic stem cell transplantation enhances the antitumor immunity of intratumoral type I interferon gene transfer for sarcoma. Hum Gene Ther.

[84] Wang Z, Wang Z, Li S (2018). Decitabine enhances Vγ9Vδ2 T cell-mediated cytotoxic effects on osteosarcoma cells via the NKG2DL-NKG2D Axis. Front Immunol.

[85] Long AH, Highfill SL, Cui Y (2016). Reduction of MDSCs with all-trans retinoic acid improves CAR therapy efficacy for sarcomas. Cancer Immunol Res.

[86] Kawano M, Tanaka K, Itonaga I (2015). Dendritic cells combined with anti-GITR antibody produce antitumor effects in osteosarcoma. Oncol Rep.

[87] Kawano M, Itonaga I, Iwasaki T (2012). Anti-TGF-β antibody combined with dendritic cells produce antitumor effects in osteosarcoma. Clin Orthop Relat Res.

[88] Zhang Y, Yuan T, Li Z (2021). Hyaluronate-based self-stabilized nanoparticles for immunosuppression reversion and immunochemotherapy in osteosarcoma treatment. ACS Biomater Sci Eng.

[89] Takahashi Y, Yasui T, Minami K (2017). Radiation enhances the efficacy of antitumor immunotherapy with an immunocomplex of interleukin-2 and its monoclonal antibody. Anticancer Res.

[90] Kawano M, Itonaga I, Iwasaki T (2013). Enhancement of antitumor immunity by combining anti-cytotoxic T lymphocyte antigen-4 antibodies and cryotreated tumor lysate-pulsed dendritic cells in murine osteosarcoma. Oncol Rep.

[91] Park JA, Cheung NV (2020). GD2 or HER2 targeting T cell engaging bispecific antibodies to treat osteosarcoma. J Hematol Oncol.

[92] Kushner BH, Cheung IY, Modak S (2018). Humanized 3F8 anti-GD2 monoclonal antibody dosing with granulocyte-macrophage colony-stimulating factor in patients with resistant neuroblastoma: a phase 1 clinical trial. JAMA Oncol.

[93] Butch ER, Mead PE, Diaz VA (2019). Positron emission tomography detects *in vivo* expression of disialoganglioside GD2 in mouse models of primary and metastatic osteosarcoma. Cancer Res.

[94] Navid F, M Santana V, C Barfield R (2010). Anti-GD2 antibody therapy for GD2-expressing tumors. Curr Cancer Drug Targets.

[95] Iclozan C, Antonia S, Chiappori A (2013). Therapeutic regulation of myeloid-derived suppressor cells and immune response to cancer vaccine in patients with extensive stage small cell lung cancer. Cancer Immunol Immunother.

[96] Mirza N, Fishman M, Fricke I (2006). All-trans-retinoic acid improves differentiation of myeloid cells and immune response in cancer patients. Cancer Res.

[97] Kusmartsev S, Cheng F, Yu B (2003). All-trans-retinoic acid eliminates immature myeloid cells from tumor-bearing mice and improves the effect of vaccination. Cancer Res.

[98] Klose C, Berchtold S, Schmidt M (2019). Biological treatment of pediatric sarcomas by combined virotherapy and NK cell therapy. BMC Cancer.

[99] Di Carlo E, Bocca P, Emionite L (2013). Mechanisms of the antitumor activity of human Vγ9Vδ2 T cells in combination with zoledronic acid in a preclinical model of neuroblastoma. Mol Ther.

[100] Sun L, Li Y, Jiang Z (2016). Vγ9Vδ2 T cells and zoledronate mediate antitumor activity in an orthotopic mouse model of human chondrosarcoma. Tumor Biol.

[101] Gajewski TF, Corrales L, Williams J (2017). Cancer immunotherapy targets based on understanding the T cell-inflamed versus non-T cell-inflamed tumor microenvironment. In: Kalinski, P, Ed. Tumor immune microenvironment in cancer progression and cancer therapy. Advances in Experimental Medicine and Biology.Springer, Cham; 2017.

[102] Fridman WH, Zitvogel L, Sautès-Fridman C (2017). The immune contexture in cancer prognosis and treatment. Nat Rev Clin Oncol.

[103] Tumeh PC, Harview CL, Yearley JH (2014). PD-1 blockade induces responses by inhibiting adaptive immune resistance. Nature.

[104] Vasaturo A, Halilovic A, Bol KF (2016). T-cell landscape in a primary melanoma predicts the survival of patients with metastatic disease after their treatment with dendritic cell vaccines. Cancer Res.

[105] Ji R, Chasalow SD, Wang L (2012). An immune-active tumor microenvironment favors clinical response to ipilimumab. Cancer Immunol Immunother.

[106] Ager A, Watson HA, Wehenkel SC (2016). Homing to solid cancers: a vascular checkpoint in adoptive cell therapy using CAR T-cells. Biochem Soc Trans.

[107] Bulgarelli J, Tazzari M, Granato AM (2019). Dendritic cell vaccination in metastatic melanoma turns “non-T cell inflamed” into “T-cell inflamed” tumors. Front Immunol.

[108] Fritzsching B, Fellenberg J, Moskovszky L (2015). CD8 /FOXP3 -ratio in osteosarcoma microenvironment separates survivors from non-survivors: a multicenter validated retrospective study. Oncoimmunology.

[109] Han Q, Shi H, Liu F (2016). CD163 M2-type tumor-associated macrophage support the suppression of tumor-infiltrating T cells in osteosarcoma. Int Immunopharmacol.

[110] Gomez-Brouchet A, Illac C, Gilhodes J (2017). CD163-positive tumor-associated macrophages and CD8-positive cytotoxic lymphocytes are powerful diagnostic markers for the therapeutic stratification of osteosarcoma patients: an immunohistochemical analysis of the biopsies from the French OS2006 phase 3 trial. Oncoimmunology.

[111] Dumars C, Ngyuen J, Gaultier A (2016). Dysregulation of macrophage polarization is associated with the metastatic process in osteosarcoma. Oncotarget.

[112] Spurny C, Kailayangiri S, Jamitzky S (2018). Programmed cell death ligand 1 (PD-L1) expression is not a predominant feature in Ewing sarcomas. Pediatr Blood Cancer.

[113] Vakkila J, Jaffe R, Michelow M (2006). Pediatric cancers are infiltrated predominantly by macrophages and contain a paucity of dendritic cells: a major nosologic difference with adult tumors. Clin Cancer Res.

[114] Sorbye SW, Kilvaer T, Valkov A (2011). Prognostic impact of lymphocytes in soft tissue sarcomas. PLoS One.

[115] Orth MF, Buecklein VL, Kampmann E (2020). A comparative view on the expression patterns of PD-L1 and PD-1 in soft tissue sarcomas. Cancer Immunol Immunother.

[116] Kather JN, Hörner C, Weis C (2019). CD163 immune cell infiltrates and presence of CD54 microvessels are prognostic markers for patients with embryonal rhabdomyosarcoma. Sci Rep.

[117] Petitprez F, de Reyniès A, Keung EZ (2020). B cells are associated with survival and immunotherapy response in sarcoma. Nature.

[118] Goff PH, Riolobos L, LaFleur BJ (2022). Neoadjuvant therapy induces a potent immune response to sarcoma, dominated by myeloid and B cells. Clin Cancer Res.

[119] Pillozzi S, Bernini A, Palchetti I (2021). Soft Tissue Sarcoma: An Insight on Biomarkers at Molecular, Metabolic and Cellular Level. Cancers.

[120] Movva S, Wen W, Chen W (2015). Multi-platform profiling of over 2000 sarcomas: identification of biomarkers and novel therapeutic targets. Oncotarget.

[121] Keung EZ, Lazar AJ, Torres KE (2018). Phase II study of neoadjuvant checkpoint blockade in patients with surgically resectable undifferentiated pleomorphic sarcoma and dedifferentiated liposarcoma. BMC Cancer.

[122] Ayers M, Lunceford J, Nebozhyn M (2017). IFN-γ-related mRNA profile predicts clinical response to PD-1 blockade. J Clin Invest.

[123] Danaher P, Warren S, Lu R (2018). Pan-cancer adaptive immune resistance as defined by the Tumor Inflammation Signature (TIS): results from The Cancer Genome Atlas (TCGA). J Immunother Cancer.

[124] Lequeux A, Noman MZ, Xiao M (2019). Impact of hypoxic tumor microenvironment and tumor cell plasticity on the expression of immune checkpoints. Cancer Lett.

[125] Hu M, Li Y, Lu Y (2021). The regulation of immune checkpoints by the hypoxic tumor microenvironment. PeerJ.

[126] Bellone M, Calcinotto A (2013). Ways to enhance lymphocyte trafficking into tumors and fitness of tumor infiltrating lymphocytes. Front Oncol.

[127] Damerell V, Pepper MS, Prince S (2021). Molecular mechanisms underpinning sarcomas and implications for current and future therapy. Signal Transduct Target Ther.

[128] Molina ER, Chim LK, Barrios S (2020). Modeling the tumor microenvironment and pathogenic signaling in bone sarcoma. Tissue Eng. Part B Rev.

[129] Jones RL, Ravi V, Brohl AS (2022). Efficacy and safety of TRC105 plus pazopanib vs pazopanib alone for treatment of patients with advanced angiosarcoma: A randomized clinical trial. JAMA Oncol.

[130] Yang L, Forker L, Irlam JJ (2018). Validation of a hypoxia related gene signature in multiple soft tissue sarcoma cohorts. Oncotarget.

[131] Jain RK (2005). Normalization of tumor vasculature: an emerging concept in antiangiogenic therapy. Science.

[132] Jain RK (2014). Antiangiogenesis strategies revisited: from starving tumors to alleviating hypoxia. Cancer cell.

[133] Hodi FS, Lawrence D, Lezcano C (2014). Bevacizumab plus ipilimumab in patients with metastatic melanoma. Cancer Immunol Res.

[134] Manegold C, Dingemans AC, Gray JE (2017). The potential of combined immunotherapy and antiangiogenesis for the synergistic treatment of advanced NSCLC. J Thorac Oncol.

[135] Wallin JJ, Bendell JC, Funke R (2016). Atezolizumab in combination with bevacizumab enhances antigen-specific T-cell migration in metastatic renal cell carcinoma. Nat Commun.

[136] Wu X, Giobbie-Hurder A, Liao X (2016). VEGF neutralization plus CTLA-4 blockade alters soluble and cellular factors associated with enhancing lymphocyte infiltration and humoral recognition in melanoma. Cancer Immunol Res.

[137] Wilky BA, Trucco MM, Subhawong TK (2019). Axitinib plus pembrolizumab in patients with advanced sarcomas including alveolar soft-part sarcoma: a single-centre, single-arm, phase 2 trial. Lancet Oncol.

[138] Jayaprakash P, Ai M, Liu A (2018). Targeted hypoxia reduction restores T cell infiltration and sensitizes prostate cancer to immunotherapy. J Clin Invest.

[139] Egeblad M, Rasch MG, Weaver VM (2010). Dynamic interplay between the collagen scaffold and tumor evolution. Curr Opin Cell Biol.

[140] Helms E, Onate MK, Sherman MH (2020). Fibroblast heterogeneity in the pancreatic tumor microenvironment. Cancer Discov.

[141] Kato T, Noma K, Ohara T (2018). Cancer-associated fibroblasts affect intratumoral CD8 and FoxP3 T cells via IL6 in the tumor microenvironment. Clin Cancer Res.

[142] Veldhoen M, Hocking RJ, Atkins CJ (2006). TGFβ in the context of an inflammatory cytokine milieu supports *de novo* differentiation of IL-17-producing T cells. Immunity.

[143] Bailey SR, Nelson MH, Himes RA (2014). Th17 cells in cancer: the ultimate identity crisis. Front Immunol.

[144] Pachva MC, Horton Lai AJ, Rouleau M (2021). Extracellular Vesicles in Reprogramming of the Ewing Sarcoma Tumor Microenvironment. Front Cell Dev Biol.

[145] Song Y, Xu Y, Deng C (2021). Gene expression classifier reveals prognostic osteosarcoma microenvironment molecular subtypes. Front Immunol.

[146] D'Agostino S, Tombolan L, Saggioro M (2020). Rhabdomyosarcoma cells produce their own extracellular matrix with minimal involvement of cancer-associated fibroblasts: A preliminary study. Front Oncol.

[147] Yoon H, Tang C, Banerjee S (2021). TGF-β1-mediated transition of resident fibroblasts to cancer-associated fibroblasts promotes cancer metastasis in gastrointestinal stromal tumor. Oncogenesis.

[148] Li B, Wang Z, Wu H (2018). Epigenetic regulation of CXCL12 plays a critical role in mediating tumor progression and the immune response in osteosarcoma. Cancer Res.

[149] Feig C, Jones JO, Kraman M (2013). Targeting CXCL12 from FAP-expressing carcinoma-associated fibroblasts synergizes with anti-PD-L1 immunotherapy in pancreatic cancer. PNAS.

[150] Avnet S, Di Pompo G, Chano T (2017). Cancer-associated mesenchymal stroma fosters the stemness of osteosarcoma cells in response to intratumoral acidosis via NF-κB activation. Int J Cancer.

[151] Taddei ML, Pietrovito L, Leo A (2020). Lactate in sarcoma microenvironment: Much more than just a waste product. Cells.

[152] Tanner JM, Bensard C, Wei P (2017). EWS/FLI is a master regulator of metabolic reprogramming in Ewing sarcoma. Mol Cancer Res.

[153] Hua G, Liu Y, Li X (2014). Targeting glucose metabolism in chondrosarcoma cells enhances the sensitivity to doxorubicin through the inhibition of lactate dehydrogenase-A. Oncol Rep.

[154] Salerno M, Avnet S, Bonuccelli G (2014). Impairment of lysosomal activity as a therapeutic modality targeting cancer stem cells of embryonal rhabdomyosarcoma cell line RD. PloS One.

[155] Lee P, Malik D, Perkons N (2020). Targeting glutamine metabolism slows soft tissue sarcoma growth. Nat Commun.

[156] Bean GR, Kremer JC, Prudner BC (2016). A metabolic synthetic lethal strategy with arginine deprivation and chloroquine leads to cell death in ASS1-deficient sarcomas. Cell Death Dis.

[157] Albaugh VL, Pinzon-Guzman C, Barbul A (2017). Arginine—Dual roles as an onconutrient and immunonutrient. J Surg Oncol.

[158] He X, Lin H, Yuan L (2017). Combination therapy with L-arginine and α-PD-L1 antibody boosts immune response against osteosarcoma in immunocompetent mice. Cancer Biol Ther.

[159] Xiao L, Yeung H, Haber M (2021). Immunometabolism: A 'Hot'Switch for 'Cold'Pediatric Solid Tumors. Trends Cancer.

[160] Urakawa H, Nishida Y, Nakashima H (2009). Prognostic value of indoleamine 2, 3-dioxygenase expression in high grade osteosarcoma. Clin Exp Metastasis.

[161] Max D, Kuehnoel CD, Burdach S (2014). Indoleamine-2, 3-dioxygenase in an immunotherapy model for Ewing sarcoma. Anticancer Res.

[162] Nafia I, Toulmonde M, Bortolotto D (2020). IDO targeting in sarcoma: biological and clinical implications. Front Immunol.

[163] Toulmonde M, Penel N, Adam J (2018). Use of PD-1 targeting, macrophage infiltration, and IDO pathway activation in sarcomas: a phase 2 clinical trial. JAMA Oncol.

[164] Gkretsi V, Stylianou A, Papageorgis P (2015). Remodeling components of the tumor microenvironment to enhance cancer therapy. Front Oncol.

[165] Stylianopoulos T, Munn LL, Jain RK (2018). Reengineering the physical microenvironment of tumors to improve drug delivery and efficacy: from mathematical modeling to bench to bedside. Trends Cancer.

[166] Stylianopoulos T, Munn LL, Jain RK (2018). Reengineering the tumor vasculature: improving drug delivery and efficacy. Trends Cancer.

[167] Martin-Broto J, Hindi N, Grignani G (2020). Nivolumab and sunitinib combination in advanced soft tissue sarcomas: a multicenter, single-arm, phase Ib/II trial. J Immunother Cancer.

[168] Duan XL, Guo JP, Li F (2020). Sunitinib inhibits PD-L1 expression in osteosarcoma by targeting STAT3 and remodels the immune system in tumor-bearing mice. Future Oncol.

[169] Stylianopoulos T, Jain RK (2013). Combining two strategies to improve perfusion and drug delivery in solid tumors. PNAS.

[170] Jayson GC, Kerbel R, Ellis LM (2016). Antiangiogenic therapy in oncology: current status and future directions. Lancet.

[171] Jain RK, Martin JD, Stylianopoulos T (2014). The role of mechanical forces in tumor growth and therapy. Annu Rev Biomed Eng.

[172] Stylianopoulos T, Martin JD, Chauhan VP (2012). Causes, consequences, and remedies for growth-induced solid stress in murine and human tumors. PNAS.

[173] Netti PA, Berk DA, Swartz MA (2000). Role of extracellular matrix assembly in interstitial transport in solid tumors. Cancer Res.

[174] Whatcott CJ, Hanl H, Von Hoff DD (2015). Orchestrating the tumor microenvironment to improve survival for patients with pancreatic cancer normalization, not destruction. Cancer J.

[175] Sheridan C (2019). Pancreatic cancer provides testbed for first mechanotherapeutics. Nat Biotechnol.

[176] Diop-Frimpong B, Chauhan VP, Krane S (2011). Losartan inhibits collagen I synthesis and improves the distribution and efficacy of nanotherapeutics in tumors. PNAS.

[177] Zhao Y, Cao J, Melamed A (2019). Losartan treatment enhances chemotherapy efficacy and reduces ascites in ovarian cancer models by normalizing the tumor stroma. PNAS.

[178] Panagi M, Voutouri C, Mpekris F (2020). TGF-β inhibition combined with cytotoxic nanomedicine normalizes triple negative breast cancer microenvironment towards anti-tumor immunity. Theranostics.

[179] Mpekris F, Panagi M, Voutouri C (2021). Normalizing the microenvironment overcomes vessel compression and resistance to nano-immunotherapy in breast cancer lung metastasis. Adv Sci.

[180] Incio J, Suboj P, Chin SM (2015). Metformin reduces desmoplasia in pancreatic cancer by reprogramming stellate cells and tumor-associated macrophages. PloS One.

[181] Martin JD, Panagi M, Wang C (2019). Dexamethasone increases cisplatin-loaded nanocarrier delivery and efficacy in metastatic breast cancer by normalizing the tumor microenvironment. ACS Nano.

[182] Polydorou C, Mpekris F, Papageorgis P (2017). Pirfenidone normalizes the tumor microenvironment to improve chemotherapy. Oncotarget.

[183] Voutouri C, Panagi M, Mpekris F (2021). Endothelin Inhibition Potentiates Cancer Immunotherapy Revealing Mechanical Biomarkers Predictive of Response. Adv Ther.

[184] Sherman MH, Ruth TY, Engle DD (2014). Vitamin D receptor-mediated stromal reprogramming suppresses pancreatitis and enhances pancreatic cancer therapy. Cell.

[185] Kim JH, Shin BC, Park WS (2017). Antifibrotic effects of pentoxifylline improve the efficacy of gemcitabine in human pancreatic tumor xenografts. Cancer Sci.

[186] Masterson R, Hewitson TD, Kelynack K (2004). Relaxin down-regulates renal fibroblast function and promotes matrix remodelling *in vitro*. Nephrol Dial Transplant.

[187] Unemori EN, Amento EP (1990). Relaxin modulates synthesis and secretion of procollagenase and collagen by human dermal fibroblasts. J Biol Chem.

[188] Panagi M, Fotios M, Voutouri C Targeting mast cells restores T cell infiltration and sensitizes sarcomas to PD-L1 inhibition. (under revision).

[189] Mpekris F, Panagi M, Michael C Translational nanomedicine regimen potentiates immune checkpoint inhibition in metastatic sarcoma by normalizing the microenvironment. (under revision).

[190] Mariathasan S, Turley SJ, Nickles D (2018). TGFβ attenuates tumour response to PD-L1 blockade by contributing to exclusion of T cells. Nature.

[191] Tauriello DV, Palomo-Ponce S, Stork D (2018). TGFβ drives immune evasion in genetically reconstituted colon cancer metastasis. Nature.

[192] Chakravarthy A, Khan L, Bensler NP (2018). TGF-β-associated extracellular matrix genes link cancer-associated fibroblasts to immune evasion and immunotherapy failure. Nat Commun.

[193] Munn LL, Jain RK (2019). Vascular regulation of antitumor immunity. Science.

[194] Vanpouille-Box C, Diamond JM, Pilones KA (2015). TGFβ is a master regulator of radiation therapy-induced antitumor immunity. Cancer Res.

[195] Ford K, Hanley CJ, Mellone M (2020). NOX4 inhibition potentiates immunotherapy by overcoming cancer-associated fibroblast-mediated CD8 T-cell exclusion from tumors. Cancer Res.

[196] Zboralski D, Hoehlig K, Eulberg D (2017). Increasing tumor-infiltrating T cells through inhibition of CXCL12 with NOX-A12 synergizes with PD-1 blockade. Cancer Immunol Res.

[197] Gao Y, Souza-Fonseca-Guimaraes F, Bald T (2017). Tumor immunoevasion by the conversion of effector NK cells into type 1 innate lymphoid cells. Nat Immunol.

[198] Premkumar K, Shankar BS (2021). TGF-βR inhibitor SB431542 restores immune suppression induced by regulatory B-T cell axis and decreases tumour burden in murine fibrosarcoma. Cancer Immunol Immunother.

[199] Gill PS, Wernz J, Scadden DT (1996). Randomized phase III trial of liposomal daunorubicin versus doxorubicin, bleomycin, and vincristine in AIDS-related Kaposi's sarcoma. J Clin Oncol.

[200] Northfelt DW, Dezube BJ, Thommes JA (1998). Pegylated-liposomal doxorubicin versus doxorubicin, bleomycin, and vincristine in the treatment of AIDS-related Kaposi's sarcoma: results of a randomized phase III clinical trial. J Clin Oncol.

[201] Kundranda MN, Niu J (2015). Albumin-bound paclitaxel in solid tumors: clinical development and future directions. Drug Des Devel Ther.

[202] Tian Z, Yao W (2022). Albumin-Bound Paclitaxel: Worthy of Further Study in Sarcomas. Front Oncol.

[203] Metts JL, Alazraki AL, Clark D (2018). Gemcitabine/nab-paclitaxel for pediatric relapsed/refractory sarcomas. Pediatr Blood Cancer.

[204] Moreno L, Casanova M, Chisholm JC (2018). Phase I results of a phase I/II study of weekly nab-paclitaxel in paediatric patients with recurrent/refractory solid tumours: a collaboration with innovative therapies for children with cancer. Eur J Cancer.

[205] Stylianopoulos T (2013). EPR-effect: utilizing size-dependent nanoparticle delivery to solid tumors. Ther Deliv.

[206] Maeda H, Wu J, Sawa T (2000). Tumor vascular permeability and the EPR effect in macromolecular therapeutics: a review. J Control Release.

[207] Shi Y, Van der Meel R, Chen X (2020). The EPR effect and beyond: Strategies to improve tumor targeting and cancer nanomedicine treatment efficacy. Theranostics.

[208] Chauhan VP, Stylianopoulos T, Martin JD (2012). Normalization of tumour blood vessels improves the delivery of nanomedicines in a size-dependent manner. Nat Nanotechnol.

[209] Youn YS, Bae YH (2018). Perspectives on the past, present, and future of cancer nanomedicine. Adv Drug Deliv Rev.

[210] Stylianopoulos T, Jain RK (2015). Design considerations for nanotherapeutics in oncology. Nanomedicine.

[211] Mpekris F, Voutouri C, Panagi M (2022). Normalizing tumor microenvironment with nanomedicine and metronomic therapy to improve immunotherapy. J Control Release.

[212] Xia T, He Q, Shi K (2018). Losartan loaded liposomes improve the antitumor efficacy of liposomal paclitaxel modified with pH sensitive peptides by inhibition of collagen in breast cancer. Pharm Dev Technol.

[213] Chauhan VP, Chen IX, Tong R (2019). Reprogramming the microenvironment with tumor-selective angiotensin blockers enhances cancer immunotherapy. PNAS.

[214] Panagi M, Mpekris F, Chen P Superior effects of polymeric micelles in reprogramming tumor microenvironment and potentiating nano-immunotherapy. (In revision).

[215] Kunjachan S, Ehling J, Storm G (2015). Noninvasive imaging of nanomedicines and nanotheranostics: principles, progress, and prospects. Chem Rev.

[216] Tatum JL (2006). Hypoxia: importance in tumor biology, noninvasive measurement by imaging, and value of its measurement in the management of cancer therapy. Int J Radiat Biol.

[217] Mees G, Dierckx R, Vangestel C (2009). Molecular imaging of hypoxia with radiolabelled agents. Eur J Nucl Med Mol Imaging.

[218] Cosgrove D, Lassau N (2010). Imaging of perfusion using ultrasound. Eur J Nucl Med Mol Imaging.

[219] Kiessling F, Fokong S, Koczera P (2012). Ultrasound microbubbles for molecular diagnosis, therapy, and theranostics. J Nucl Med.

[220] Svensson SF, Fuster-Garcia E, Latysheva A (2022). Decreased tissue stiffness in glioblastoma by MR elastography is associated with increased cerebral blood flow. Eur J Radiol.

[221] Sun Q, Bai X, Sofias AM (2020). Cancer nanomedicine meets immunotherapy: opportunities and challenges. Acta Pharmacol Sin.

[222] Shi Y, Lammers T (2019). Combining nanomedicine and immunotherapy. Acc Chem Res.

[223] Martin JD, Cabral H, Stylianopoulos T (2020). Improving cancer immunotherapy using nanomedicines: progress, opportunities and challenges. Nat Rev Clin Oncol.

[224] Tian Z, Dong S, Yang Y (2022). Nanoparticle albumin-bound paclitaxel and PD-1 inhibitor (sintilimab) combination therapy for soft tissue sarcoma: a retrospective study. BMC Cancer.

[225] Shi Y (2020). Clinical translation of nanomedicine and biomaterials for cancer immunotherapy: progress and perspectives. Adv Ther.

[226] Chou AJ, Kleinerman ES, Krailo MD (2009). Addition of muramyl tripeptide to chemotherapy for patients with newly diagnosed metastatic osteosarcoma: a report from the Children's Oncology Group. Cancer.

[227] Wang DY, Salem J, Cohen JV (2018). Fatal toxic effects associated with immune checkpoint inhibitors: a systematic review and meta-analysis. JAMA Oncol.

[228] Martins F, Sofiya L, Sykiotis GP (2019). Adverse effects of immune-checkpoint inhibitors: epidemiology, management and surveillance. Nat Rev Clin Oncol.

[229] Merry E, Thway K, Jones RL (2021). Predictive and prognostic transcriptomic biomarkers in soft tissue sarcomas. NPJ Precis Oncol.

[230] Somaiah N, Chawla SP, Block MS (2020). A phase 1B study evaluating the safety, tolerability, and immunogenicity of CMB305, a lentiviral-based prime-boost vaccine regimen, in patients with locally advanced, relapsed, or metastatic cancer expressing NY-ESO-1. Oncoimmunology.

[231] Noguchi T, Ward JP, Gubin MM (2017). Temporally distinct PD-L1 expression by tumor and host cells contributes to immune escape. Cancer Immunol Res.

[232] Hosoi H, Ikeda H, Imai N (2014). Stimulation through very late antigen-4 and-5 improves the multifunctionality and memory formation of CD8 T cells. Eur J Immunol.

[233] Mardiana S, John LB, Henderson MA (2017). A multifunctional role for adjuvant anti-4-1BB therapy in augmenting antitumor response by chimeric antigen receptor T cells. Cancer Res.

[234] Arab S, Motamedi M, Hadjati J (2019). Effects of dendritic cell vaccine activated with components of Lieshmania major on tumor specific response. Iran J Immunol.

[235] Huijbers EJ, van Beijnum JR, Lê CT (2018). An improved conjugate vaccine technology; induction of antibody responses to the tumor vasculature. Vaccine.

[236] Koido S, Ito M, Sagawa Y (2014). Vaccination with vascular progenitor cells derived from induced pluripotent stem cells elicits antitumor immunity targeting vascular and tumor cells. Cancer Immunol Immunother.

[237] Li X, Wang Y, Zhao Y (2014). Immunotherapy of tumor with vaccine based on basic fibroblast growth factor-activated fibroblasts. J Cancer Res Clin Oncol.

[238] Chen L, Taylor JL, Sabins NC (2013). Extranodal induction of therapeutic immunity in the tumor microenvironment after intratumoral delivery of Tbet gene-modified dendritic cells. Cancer Gene Ther.

[239] Huang C, Ramakrishnan R, Trkulja M (2012). Therapeutic effect of intratumoral administration of DCs with conditional expression of combination of different cytokines. Cancer Immunol Immunother.

[240] Azadmehr A, Pourfathollah AA, Amirghofran Z (2013). Immunotherapy with tumor cell lysate-pulsed CD8α dendritic cells modulates intra-tumor and spleen lymphocyte subpopulations. Neoplasma.

[241] Wang Y, Liu S, Yuan M (2015). Prophylactic antitumor effect of mixed heat shock proteins/peptides in mouse sarcoma. Chin Med J.

[242] Takamura-Ishii M, Miura T, Nakaya T (2017). Induction of antitumor response to fibrosarcoma by Newcastle disease virus-infected tumor vaccine. Med Oncol.

[243] Gubin MM, Zhang X, Schuster H (2014). Checkpoint blockade cancer immunotherapy targets tumour-specific mutant antigens. Nature.

[244] Ebrahimi-Nik H, Corwin WL, Shcheglova T (2018). CD11c MHCII lo GM-CSF-bone marrow-derived dendritic cells act as antigen donor cells and as antigen presenting cells in neoepitope-elicited tumor immunity against a mouse fibrosarcoma. Cancer Immunol Immunother.

[245] Gasparri AM, Sacchi A, Basso V (2019). Boosting interleukin-12 antitumor activity and synergism with immunotherapy by targeted delivery with isoDGR-tagged nanogold. Small.

[246] Balza E, Zanellato S, Poggi A (2017). The therapeutic T-cell response induced by tumor delivery of TNF and melphalan is dependent on early triggering of natural killer and dendritic cells. Eur J Immunol.

[247] Razi Soofiyani S, Kazemi T, Lotfipour F (2016). Gene therapy with IL-12 induced enhanced anti-tumor activity in fibrosarcoma mouse model. Artif Cells Nanomed Biotechnol.

[248] Whelan MC, Casey G, Larkin JO (2014). Oral tolerance to cancer can be abrogated by T regulatory cell inhibition. PLoS One.

[249] Hess C, Neri D (2015). The antibody-mediated targeted delivery of interleukin-13 to syngeneic murine tumors mediates a potent anticancer activity. Cancer Immunol Immunother.

[250] Vlková V, Štěpánek I, Hrušková V (2014). Epigenetic regulations in the IFNγ signalling pathway: IFNγ-mediated MHC class I upregulation on tumour cells is associated with DNA demethylation of antigen-presenting machinery genes. Oncotarget.

[251] Aoki R, Iijima H, Kato M (2014). Protein-bound polysaccharide-K reduces the proportion of regulatory T cells *in vitro* and *in vivo*. Oncol Rep.

[252] Peinado C, Kang X, Hardamon C (2013). The nuclear factor-κB pathway down-regulates expression of the NKG 2D ligand H60a *in vitro*: implications for use of nuclear factor-κB inhibitors in cancer therapy. Immunology.

[253] Molgora M, Esaulova E, Vermi W (2020). TREM2 modulation remodels the tumor myeloid landscape enhancing anti-PD-1 immunotherapy. Cell.

[254] Travelli C, Consonni FM, Sangaletti S (2019). Nicotinamide phosphoribosyltransferase acts as a metabolic gate for mobilization of myeloid-derived suppressor cells. Cancer Res.

[255] Hurst KE, Lawrence KA, Essman MT (2019). Endoplasmic reticulum stress contributes to mitochondrial exhaustion of CD8 T cells. Cancer Immunol Res.

[256] Tu MM, Lee FY, Jones RT (2019). Targeting DDR2 enhances tumor response to anti-PD-1 immunotherapy. Sci Adv.

[257] Kjaergaard J, Hatfield S, Jones G (2018). A2A adenosine receptor gene deletion or synthetic A2A antagonist liberate tumor-reactive CD8 T cells from tumor-induced immunosuppression. J Immunol.

[258] Khalili A, Muhammad Hassan Z, Shahabi S (2013). Long acting propranolol and HSP-70 rich tumor lysate reduce tumor growth and enhance immune response against fibrosarcoma in Balb/c mice. Iran J Immunol.

[259] Nakashima H, Terabe M, Berzofsky JA (2011). A novel combination immunotherapy for cancer by IL-13Rα2-targeted DNA vaccine and immunotoxin in murine tumor models. J Immunol.

[260] Behzadi E, Hosseini HM, Halabian R (2017). Macrophage cell-derived exosomes/staphylococcal enterotoxin B against fibrosarcoma tumor. Microb Pathog.

[261] Redmond WL, Triplett T, Floyd K (2012). Dual anti-OX40/IL-2 therapy augments tumor immunotherapy via IL-2R-mediated regulation of OX40 expression. PloS One.

[262] Sturgill ER, Rolig AS, Linch SN (2021). Galectin-3 inhibition with belapectin combined with anti-OX40 therapy reprograms the tumor microenvironment to favor anti-tumor immunity. Oncoimmunology.

[263] Farazi M, Nguyen J, Goldufsky J (2014). Caloric restriction maintains OX40 agonist-mediated tumor immunity and CD4 T cell priming during aging. Cancer Immunol Immunother.

[264] van Hooren L, Georganaki M, Huang H (2016). Sunitinib enhances the antitumor responses of agonistic CD40-antibody by reducing MDSCs and synergistically improving endothelial activation and T-cell recruitment. Oncotarget.

[265] Walker JM, Rolig AS, Charych DH (2020). NKTR-214 immunotherapy synergizes with radiotherapy to stimulate systemic CD8 T cell responses capable of curing multi-focal cancer. J Immunother Cancer.

[266] Romero I, Garrido C, Algarra I (2018). MHC intratumoral heterogeneity may predict cancer progression and response to immunotherapy. Front Immunol.

[267] Corbellari R, Nadal L, Villa A (2020). The immunocytokine L19-TNF eradicates sarcomas in combination with chemotherapy agents or with immune check-point inhibitors. Anticancer Drugs.

[268] Soto-Pantoja DR, Terabe M, Ghosh A (2014). CD47 in the tumor microenvironment limits cooperation between antitumor T-cell immunity and radiotherapy. Cancer Res.

[269] Dhupkar P, Gordon N, Stewart J (2018). Anti-PD-1 therapy redirects macrophages from an M2 to an M1 phenotype inducing regression of OS lung metastases. Cancer Med.

[270] Zheng B, Ren T, Huang Y (2018). PD-1 axis expression in musculoskeletal tumors and antitumor effect of nivolumab in osteosarcoma model of humanized mouse. J Hematol Oncol.

[271] Shimizu T, Fuchimoto Y, Fukuda K (2017). The effect of immune checkpoint inhibitors on lung metastases of osteosarcoma. J Pediatr Surg.

[272] Helm A, Tinganelli W, Simoniello P (2021). Reduction of lung metastases in a mouse osteosarcoma model treated with carbon ions and immune checkpoint inhibitors. Int J Radiat Oncol Biol Phys.

[273] Guiho R, Biteau K, Grisendi G (2018). *In vitro* and *in vivo* discrepancy in inducing apoptosis by mesenchymal stromal cells delivering membrane-bound tumor necrosis factor-related apoptosis inducing ligand in osteosarcoma pre-clinical models. Cytotherapy.

[274] Majzner RG, Theruvath JL, Nellan A (2019). CAR T cells targeting B7-H3, a pan-cancer antigen, demonstrate potent preclinical activity against pediatric solid tumors and brain tumors. Clin Cancer Res.

[275] Wang Y, Yu W, Zhu J (2019). Anti-CD166/4-1BB chimeric antigen receptor T cell therapy for the treatment of osteosarcoma. J Exp Clin Cancer Res.

[276] Fernández L, Metais J, Escudero A (2017). Memory T cells expressing an NKG2D-CAR efficiently target osteosarcoma cells. Clin Cancer Res.

[277] Huang X, Park H, Greene J (2015). IGF1R-and ROR1-specific CAR T cells as a potential therapy for high risk sarcomas. PLoS One.

[278] Rainusso N, Brawley VS, Ghazi A (2012). Immunotherapy targeting HER2 with genetically modified T cells eliminates tumor-initiating cells in osteosarcoma. Cancer Gene Ther.

[279] Englisch A, Altvater B, Kailayangiri S (2020). VEGFR2 as a target for CAR T cell therapy of Ewing sarcoma. Pediatr Blood Cancer.

[280] Liebsch L, Kailayangiri S, Beck L (2013). Ewing sarcoma dissemination and response to T-cell therapy in mice assessed by whole-body magnetic resonance imaging. Br J Cancer.

[281] Blaeschke F, Thiel U, Kirschner A (2016). Human HLA-A* 02: 01/CHM1 allo-restricted T cell receptor transgenic CD8 T Cells specifically inhibit Ewing sarcoma growth *in vitro* and *in vivo*. Oncotarget.

[282] Imamura M, Shook D, Kamiya T (2014). Autonomous growth and increased cytotoxicity of natural killer cells expressing membrane-bound interleukin-15. Blood.

[283] Ojo EO, Sharma AA, Liu R (2019). Membrane bound IL-21 based NK cell feeder cells drive robust expansion and metabolic activation of NK cells. Sci Rep.

[284] Evans CH, Liu F, Porter RM (2012). EWS-FLI-1-targeted cytotoxic T-cell killing of multiple tumor types belonging to the Ewing sarcoma family of tumors. Clin Cancer Res.

[285] Le Boeuf F, Selman M, Son HH (2017). Oncolytic maraba virus MG1 as a treatment for sarcoma. Int J Cancer.

[286] Mochizuki Y, Tazawa H, Demiya K (2021). Telomerase-specific oncolytic immunotherapy for promoting efficacy of PD-1 blockade in osteosarcoma. Cancer Immunol Immunother.

[287] Huijbers EJ, Van Der Werf, Inge M, Faber LD (2019). Targeting tumor vascular CD99 inhibits tumor growth. Front Immunol.

[288] Fang X, Jiang C, Xia Q (2015). Effectiveness evaluation of dendritic cell immunotherapy for osteosarcoma on survival rate and *in vitro* immune response. Genet Mol Res.

[289] Yu Z, Qian J, Wu J (2012). Allogeneic mRNA-based electrotransfection of autologous dendritic cells and specific antitumor effects against osteosarcoma in rats. Med Oncol.

[290] Zhao H, Zhao X, Du P (2016). Construction of random tumor transcriptome expression library for creating and selecting novel tumor antigens. Tumor Biol.

[291] Ghosh S, Sarkar M, Ghosh T (2016). Absence of CD4 T cell help generates corrupt CD8 effector T cells in sarcoma-bearing Swiss mice treated with NLGP vaccine. Immunol Lett.

[292] Zhou Y, Slone N, Chrisikos TT (2020). Vaccine efficacy against primary and metastatic cancer with *in vitro*-generated CD103 conventional dendritic cells. J Immunother Cancer.

[293] Xu J, Pan X, Zhang S (2015). CD47 blockade inhibits tumor progression human osteosarcoma in xenograft models. Oncotarget.

[294] Brennecke P, Arlt MJ, Campanile C (2014). CXCR4 antibody treatment suppresses metastatic spread to the lung of intratibial human osteosarcoma xenografts in mice. Clin Exp Metastasis.

[295] Yu G, Li A, Li X (2017). Bispecific antibody suppresses osteosarcoma aggressiveness through regulation of NF-κB signaling pathway. Tumor Biol.

[296] Karkare S, Allen KJ, Jiao R (2019). Detection and targeting insulin growth factor receptor type 2 (IGF2R) in osteosarcoma PDX in mouse models and in canine osteosarcoma tumors. Sci Rep.

[297] Geller DS, Morris J, Revskaya E (2016). Targeted therapy of osteosarcoma with radiolabeled monoclonal antibody to an insulin-like growth factor-2 receptor (IGF2R). Nucl Med Biol.

[298] Mohanty S, Yerneni K, Theruvath JL (2019). Nanoparticle enhanced MRI can monitor macrophage response to CD47 mAb immunotherapy in osteosarcoma. Cell Death Dis.

[299] Li B, Wang Z, Wu H (2018). Epigenetic regulation of CXCL12 plays a critical role in mediating tumor progression and the immune response in osteosarcoma. Cancer Res.

[300] Yahiro K, Matsumoto Y, Yamada H (2020). Activation of TLR4 signaling inhibits progression of osteosarcoma by stimulating CD8-positive cytotoxic lymphocytes. Cancer Immunol Immunother.

[301] Gao X, Han D, Fan W (2016). Down-regulation of RBP-J mediated by microRNA-133a suppresses dendritic cells and functions as a potential tumor suppressor in osteosarcoma. Exp Cell Res.

[302] Zhou B, Liu M, Qiu X (2013). A novel recombinant immunocasp-6 fusion gene specifically and efficiently suppresses HER2-overexpressing osteosarcoma. Oncol Rep.

[303] Liu S, Zheng L, Aweya JJ (2017). Litopenaeus vannamei hemocyanin exhibits antitumor activity in S180 mouse model *in vivo*. PloS One.

[304] Wang C, Lu C, Hsueh Y (2014). Activation of antitumor immune responses by Ganoderma formosanum polysaccharides in tumor-bearing mice. Appl Microbiol Biotechnol.

[305] Zong S, Li J, Ye Z (2020). Lachnum polysaccharide suppresses S180 sarcoma by boosting anti-tumor immune responses and skewing tumor-associated macrophages toward M1 phenotype. Int J Biol Macromol.

[306] Zang X, Zhang X, Hu H (2019). Targeted delivery of zoledronate to tumor-associated macrophages for cancer immunotherapy. Mol Pharm.

[307] Li Q, Hao Z, Hong Y (2018). Reprogramming tumor associated macrophage phenotype by a polysaccharide from Ilex asprella for sarcoma immunotherapy. Int J Mol Sci.

[308] Wang T, Liu X, Ji Z (2015). Antitumor and immunomodulatory effects of recombinant fusion protein rMBP-NAP through TLR-2 dependent mechanism in tumor bearing mice. Int Immunopharmacol.

[309] Li X, Meng Y, Plotnikoff NP (2015). Methionine enkephalin (MENK) inhibits tumor growth through regulating CD4 Foxp3 regulatory T cells (Tregs) in mice. Cancer Biol Ther.

[310] Li W, Chen W, Herberman RB (2014). Immunotherapy of cancer via mediation of cytotoxic T lymphocytes by methionine enkephalin (MENK). Cancer Lett.

[311] Huang Z, Yang Y, Jiang Y (2013). Anti-tumor immune responses of tumor-associated macrophages via toll-like receptor 4 triggered by cationic polymers. Biomaterials.

[312] Barik S, Banerjee S, Mallick A (2013). Normalization of tumor microenvironment by neem leaf glycoprotein potentiates effector T cell functions and therapeutically intervenes in the growth of mouse sarcoma. PLoS One.

[313] Ghosh S, Sarkar M, Ghosh T (2017). Neem leaf glycoprotein generates superior tumor specific central memory CD8 T cells than cyclophosphamide that averts post-surgery solid sarcoma recurrence. Vaccine.

[314] He X, Lin H, Yuan L (2017). Combination therapy with L-arginine and α-PD-L1 antibody boosts immune response against osteosarcoma in immunocompetent mice. Cancer Biol Ther.

[315] Wu W, Jing D, Meng Z (2020). FGD1 promotes tumor progression and regulates tumor immune response in osteosarcoma via inhibiting PTEN activity. Theranostics.

[316] Charan M, Dravid P, Cam M (2020). GD2-directed CAR-T cells in combination with HGF-targeted neutralizing antibody (AMG102) prevent primary tumor growth and metastasis in Ewing sarcoma. Int J Cancer.

[317] Shimizu T, Fuchimoto Y, Okita H (2018). A curative treatment strategy using tumor debulking surgery combined with immune checkpoint inhibitors for advanced pediatric solid tumors: An *in vivo* study using a murine model of osteosarcoma. J Pediatr Surg.

[318] Wang J, Hu C, Wang J (2019). Checkpoint blockade in combination with doxorubicin augments tumor cell apoptosis in osteosarcoma. J Immunother.

[319] Kansara M, Thomson K, Pang P (2019). Infiltrating myeloid cells drive osteosarcoma progression via GRM4 regulation of IL23. Cancer Discov.

[320] Wang C, Zhou X, Li W (2017). Macrophage migration inhibitory factor promotes osteosarcoma growth and lung metastasis through activating the RAS/MAPK pathway. Cancer Lett.

