# Supplementary material for: Immunotherapy in soft tissue and bone sarcoma: unraveling the barriers to effectiveness
Source: Theranostics. 2022 Aug 15;12(14):6106–29. doi: 10.7150/thno.72800 (PMC9475460; doi:10.7150/thno.72800)
Supplement: Supplementary file 1 — Supplementary tables. [file thnov12p6106s1.pdf]

No data	Worsen outcome (vs control)	No change (vs control)	Similar improved outcome (monotherapy ≈ combination therapy)	Improved outcome of monotherapy (vs control)	Improved outcome of combination therapy (vs monotherapy)

Table S1: Soft tissue sarcoma

	Types of Immunotherapy					Other	Treatment output				Refs
	**ICIs**	**ACT**	**Oncolytic viruses**	**Cancer Vaccines**	**In situ vaccines**	**CT/RT**	**Primary tumor growth**	**Metastasis**	**Overall Survival**	**Immunophenotype**	
**FB**	genetic editing of PD-L1 expression						-extinguishing PD-L1 expression genetically from sarcoma cells (T3ΔPDL1) facilitated significant tumor rejection	No data	No data	No data	[1]
**FB**	anti-PD-1 +/- anti-CTLA-4			mLama4 (28-mer)/ mAlg8 (21-mer) synthetic long peptides (SLPs) plus poly(I:C)			-tumor regression was observed only in mice vaccinated with mLama4 and mAlg8 SLP plus poly(I:C)	No data	-only mice vaccinated with mLama4 + mAlg8 SLP + poly(I:C) combination displayed a significant extension in survival	-tumor-specific mutant antigens (TSMA) and Alg8 and Lama4 are responsible for CD8+ T cell response induced following anti-PD-1 therapy Alg8 and Lama4<br>-TIL specific for mLama4 or mAlg8 from mice treated with anti-PD-1 and/or anti-CTLA-4 displayed lower cell surface expression of LAG-3 and TIM-3, and higher Gzmb expression compared to control mAb-treated mice	[2]
**FB**	anti-CTLA-4			Neo1-pulsed splenocytes or BMDM-Neo1 or Neo1 DCs-Neo1			-DC-Neo1 immunized mice were the only group which showed tumor protection which was further enhanced in the presence of CTLA-4 blockade<br>-CTLA-4 blockade caused a moderate anti-tumor effect when combined with Neo1-pulsed splenocytes or BMDM-Neo1 immunization	No data	No data	-GM-CSF-CD11c+ MHC class II low DC subset was found to have the most powerful adjuvanticity of all the tested APCs and these APCs act as both an ADC (antigen donor cells) as well as an APC in vivo	[3]
**FB**	anti-PD-1				TREM2 mAb		-anti-TREM2 and anti-PD-1 caused an incomplete control of tumor growth, while	No data	No data	-combination therapy and anti-TREM2 alone reduced	[4]

							combination of anti-TREM2 and suboptimal anti-PD-1 conferred complete tumor control in all mice			myeloid cells, while anti-PD-1 and<br>treated mice evidenced no change<br>-anti-TREM2 treatment augments IFNγ and TNFα production by intra-tumoral CD8+ and CD4+ T cells, respectively (ex vivo)<br>-anti-TREM2 treatment, with or without anti-PD-1, induced de novo appearance of Nos2- Macs-t	
**FB**	anti-CTLA-4				agonist anti-OX40		-anti-OX40 therapy in the presence of CTLA-4 blockade augmented the tumor growth inhibition, while anti-OX40 or anti-CTLA-4 had limited effects	No data	-combined immunotherapy significantly enhanced survival	-only combined anti-OX40/anti-CTLA-4 therapy boosted intratumoral CD4 and CD8 T-cell expansion and differentiation but did not alter the ratio of Teff to Treg or the Treg suppressor function	[5]
**FB**	anti-PD-1				RAR signaling antagonist (BMS493)		- BMS493 synergized with systemic anti-PD-1 therapy inducing a more potent tumor growth inhibition compared to monotherapy groups	No data	-BMS493-anti-PD-1 combination extends overall survival	-T cell-derived IL13 can induce RA production by tumor cells<br>-RA inhibited DC and promoted immune suppressive macrophage differentiation from tumor monocytes<br>-RA suppresses DC differentiation by downregulated transcription factor Irf4<br>-inhibition of retinoic acid production by BMS493 decreased frequency of TAMs, increased activation markers on APCs, and increased frequency of CD4+ T cells	[6]
**FB**	anti-PD-L1				IDO inhibitor (GDC-0919)		-DC-0919 did not significantly affect tumor growth neither enhanced the	No data	-IDO inhibition did not confer any significant	-anti-PD-L1 promoted the upregulation of TNFα, IL6, IFNγ, IL2, and TGFβ	[7]

							tumor inhibition induced anti-PD-L1 monotherapy		benefit to anti-PD-L1 treatment which slightly improved survival compared to control mice	induction and favored T cell infiltration expressing IFNγ, while its combination with GDC-0919 did not change the observed features<br>-anti-PD-L1 limited, MDSCs and TAMs by promoting M1 cell state<br>-GDC-0919 alone or in combination with ICI had no impact on MSDCs	
**FB**	anti-PD-1				NAMPT (nicotinamide phosphoribosyltransferase) inhibitors (FK866 or MV87)		-combination of an anti-PD-1 and MV87 displayed a significantly higher antitumor activity as compared with the single treatments	-NAMPT inhibitors inhibited spontaneous lung metastasis formation and this effect was enhanced upon combination with anti-PD-1<br>-no significant differences were found between ICI and ICI+NAMPT inhibition	No data	-NAMPT inhibitor prevented MDSC mobilization by blocking the suppression of CXCR4 gene expression, but did not affect TAMs<br>-NAMPT inhibition increased CD3+ T cells but Tregs remained unaffected	[8]
**FB**	anti-PD-1				PERK inhibitor		-combination of PERK inhibitor and anti-PD-1 significantly promoted tumor regression	No data	-all mice treated with combination therapy–treated mice survived, while mice treated with either PERK inhibitor and anti-PD-1 exhibited a moderate increase in survival	-PERK inhibition overcomes mitochondrial exhaustion in T cells and increases CD8+ TILs	[9]
**FB**	anti-PD-L1/ anti-CTLA-4				L19-mIL12		-L19-mIL12 alone inhibited tumor growth, while its	No data	No data	No data	[10]

							combination with either of ICI enhanced the anti-tumor effect<br>-ICI alone did not have any effect				
**FB**	anti-PD-1				L19-mTNF	Dacarbazine / trabectedin/ melphalan	-L19- mTNF caused a moderate reduction in tumor size, while its combination with either chemotherapy drug enhanced tumor regression<br>-L19- mTNF enhanced the anti-tumor effect of ICI and melphalan drug<br>-ICI alone had a moderate effect	No data	-survival was prolonged upon combination of L19-mTNF with either dacarbazine or trabectedin or melphalan or PD-1 blockade	No data	[11]
**FB**	anti-PD-L1				ketotifen	doxorubicin	-ICI combined with doxorubicin or ketotifen reduced tumor volume<br>-ICI+ doxorubicin+ ketotifen had superior effect on growth inhibition	No data	No data	-ICI-ketotifen combination with or without doxorubicin increased TILs and ratio of CD8+ T cells to Tregs	[12]
**FB**	anti-PD-L1					Epirubicin (NC6300)	- ICI combined with NC6300 resulted in tumor regression compared to anti-PD-L1 monotherapy	No data	No data	-combination treatment increased TILs and intratumoral CD8 levels and proliferation	[13]
**FB**		T cells primed with anti-CD3 +/- CH-296					-tumor growth in mice receiving transgenic mouse-derived T cells primed with anti-CD3 in combination with CH-296 was significantly inhibited compared with the other groups (anti-CD3 or CH-294 stimulation)	No data	No data	-Tregs in tumor decreased in mice receiving tumor-specific CD8+ T cells primed with CH-296<br>-CD8+ to Tregs ratio was enhanced upon ACT stimulated with both anti-CD3 and CH-296	[14]
**FB**		NK cells/ PBMCs	oncolytic measles vaccine (MeV)				-Co-culture of MeV and NK cells or PBMCs resulted in higher oncolysis rates than MeV monotherapy in sarcoma cells	No data	No data	No data	[15]
**FB**		TDLN T cells			A2AR-antagonist (KW6002, istradefylline)		No data	-combination of KW6002 enhanced the therapeutic effect of wild-type TDLN T ACT	No data	-KW6002 increased the release of IFNγ from TDLN T cells	[16]

								against well established pulmonary metastases, while KW6002 by itself had modest therapeutic effect			
**FB**		CD8+ T cells			CD47 blockade (morpholino)	RT	-RT enhanced the antitumor effect of CD47 blockade and/or ACT treatment<br>-CD47 blockade alone or combined with RT did not affect tumor growth	No data	No data	-blockade of CD47 enhanced CD8+ T-cell tumoricidal activity and recruitment into irradiated tumors and increased GZMB expression independent of increased CD8+ T-cell infiltration<br>-No change in Foxp3 expression	[17]
**FB**				DCs exposed to Leishmania major's lysate (L.m-DCs) or LPS (LPS-DCs)			-LPS-DCs and L.m-DCs diminished the growth rate of tumors more significantly than immature DCs	No data	-LPS-DCs and L.m-DCs significantly extended overall survival	-DCs matured by either L.m or LPS had high levels of IL12 compared to immature DCs in vitro<br>-LPS-DCs and L.m-DCs significantly increased CD8+ T cell accumulation in tumors	[18]
**FB**				TRXtr-EDB vaccine			-TRXtr-EDB outperformed the non-truncated form of TRX (TRX-EDB) at inhibiting tumor growth	No data	No data	-TRXtr-EDB vaccination decreased vascular density and increased intratumoral CD45+ infiltration	[19]
**FB**				DC/iVP (induced vascular progenitor cells)/ DC/iPS (induced pluripotent stem)/ DC/CMS-4 (lysate from CMS-4 FS tumor)			-DC/iVP immunized mice exhibited a greater reduction in tumor size compared to DC/iPS or DC/CMS-4 immunization	No data	-DC/iVP-immunized mice showed the longest survival	-both DC/iVP and iVP immunization led to a marked decrease in tumor vasculature<br>-CD8+ T cells from DC/iVP-vaccinated mice showed significant cytotoxic activity against murine endothelial cells and FS cells, whereas CD8+ T cells from DC/iPS-vaccinated mice did not	[20]

**FB**				bFGF-activated fibroblasts			-tumor growth rate was significantly decreased in the immunized group	No data	-apparent increase in lifespan of immunized group compared to the control	No data	[21]
**FB**				mDC.Tbet			-mDC.Tbet, but not control mDC.Null, slowed tumor growth	No data	No data	-mDC.Tbet promoted early recruitment/activation of Type-1 CD8+ T cells and NK cells within the therapeutic TME in association with enhanced production of chemokines, independently of the proinflammatory cytokines IL12 family members or IFNγ	[22]
**FB**				DCs-IL12/ DCs-IFN-α based on Rheoswitch Therapeutic System (RTS)			-DC-IL12 and activator ligand (AL) resulted in a potent antitumor effect	No data	No data	-increased intratumoral levels of CD4+ and CD8+ T cells upon DC-IL12 and AL treatment compared to control mice<br>-no alteration in the MDSC levels in spleen	[23]
**FB**				TL-CD8α+DCs			-TL-CD8α+ DCs and CD8α+DCs immunization decreased tumor growth rate in a similar manner	No data	No data	-TL-CD8α+ DCs significantly enhanced both CD4+ and CD8+ lymphocytes whereas decreased Tregs in TME and spleen compared to CD8α+DCs or PBS groups<br>-significant increase in CD8+ or CD4+ T cell/ Tregs ratio in tumor	[24]
**FB**				mHSP/Ps vaccine			-mice immunized with mHSP/Ps showed tumor regression	No data	-mice immunized with mHSP/Ps showed long-term survival and long-term memory since rehallenging with FS tumor cells did not led	No data	[25]

									to tumor development		
**FB**				rNDV-TV vaccine			-tumor exclusion decreased in mice receiving immunized CD4+ and CD8+ cells, compared with those receiving immunized CD3+ cells	No data	-transfusion of scid mice with immunized CD3+ or splenocytes with rNDV after FS cell inoculation extended survival	-CD4+ and CD8+, NK and NKT cells contributed to the antitumor response	[26]
**FB**				HSP-70 rich lysate vaccine	propranolol		-tumors in mice receiving HSP70-rich lysate grew more slowly than those in the control groups<br>-Co-treatment (HSP70-enriched lysate + propranolol) caused a more effective growth control	No data	No data	-co-treatment with propranolol induced a significant increase in IFNγ production in splenocytes relative to the HSP70-enriched lysate group<br>-T cell cytotoxicity was increased compared to the untreated group	[27]
**FB**				DC/EphA2 vaccination	HSP90 Inhibitor (17-DMAG)		-HSP90 inhibition reduced tumor growth rate<br>-combination vaccination + 17-DMAG immunotherapy yields superior antitumor efficacy compared with treatment with either single modality	No data	-DC/EphA2 vaccination or the adoptive transfer of EphA2-specific CD8+ T cells plus 17-DMAG cotreatment yielded a superior tumor therapeutic regimen that was capable of rendering animals free of disease	-HSP90 inhibition alone increased tumor-infiltrating CD4+ and CD8+ T effector cells, and reduced Tregs and MDSCs, while its combination with DC/EphA2 vaccine further enhanced this immune response and yield increased in recognition of tumor cells by Type-1 anti-EphA2 CD8+ T cells	[28]
**FB**					anti-CD25		-mice that were permanently Treg depleted (by anti-CD25) were completely cured of their subcutaneous tumor		-mice that were permanently Treg depleted remained disease free	-increase in the number of Teff cells that were Tregs permanently depleted	[29]

**FB**					Iso1/Au/IL12 nanodrug		-increased reduction in tumor volume upon Iso1/Au/IL12 treatment compared to either Au/IL12 or IL12	No data	-Iso1/Au/IL12 treatment prolonged survival compared to IL12	-iso1/Au/IL12 Increased the Levels of TNF-R2, IFNγ and MIP-2 in the TME<br>-Increased infiltration of CD11b+ cells, granulocytes, monocytes, and NK cells<br>-anti-tumor effect was NK dependent	[30]
**FB**					L19mTNF in combination with melphalan (TP)		No data	No data	No data	-increased splenic CD4+ and CD8+ T cells associated with higher levels of IFNγ production compared to untreated control<br>-significant enhancement of NKs and mature DCs in TDLNs and a reduction of infiltrating CD206+ M2 macrophages	[31]
**FB**					IL12 gene therapy		-IL12 gene therapy significantly reduced tumor volume compared to the untreated mice	No data	No data	-IL12 gene therapy upregulated intratumoral levels of IFNγ and reduced tumor cell proliferation	[32]
**FB**					F8-IL13 fusion protein		-F8-IL13 fusion protein caused a strong inhibition of tumor growth rate compared to either anti-IL13 or F8 treatment	No data	-F8-IL13 therapy produced a memory response as none of the cured F8-IL13 treated mice developed FS tumors after cancer cell rechallenge	-F8-IL13 accumulated around tumor neo-vascular structures	[33]
**FB**					Genetic ablation of TGFβ signaling specifically in NKp46+ cells		No data	No data	-ablation of TGFβ signaling prolonged survival	-TGFβ-signaling-dependent conversion of NK cells<br>-TGFβ inhibition decreased the frequencies of intILC1 and ILC1 populations in TME	[34]

**FB**					TGFβR inhibitor (SB431542)		-TGFβR inhibition reduced tumor growth	No data	No data	-tumor-conditioned medium (TCM) induced Treg up-regulation by Breg cells expressing TGFβ1/2 which in turn suppress T cell responses to antigens<br>-SB431542 treatment significantly increased levels of splenic CD4+ and CD8+ cells and reduced Tregs with no change in CD19+ cells	[35]
**FB**					IFNγ		No data	No data	No data	-IFNγ treatment upregulated the expression of antigen-presenting machinery genes by DNA demethylation of selected antigen-presenting machinery genes located in the MHC genomic locus	[36]
**FB**					Protein-bound polysaccharide-K (PSK)		-PSK significantly reduced tumor growth	No data	No data	-reduced the proportion of Tregs in the spleen and the plasma TGFβ concentration, and significantly increased the CD8+/Treg ratio in the spleen and IFNγ production by spleen cells	[37]
**FB**					Exosomes (EXO):<br>cell lysate treated EXO (EXOLys)/ HSP70 enriched FS cell lysate treated EXO (EXO_{HSP70})/ Naloxone treated EXO (EXONLX)<br><br>+/- staphylococcal		-EXO_{HSP} was more effective than EXO and EXO_{Lys} to decrease the number of tumor cells and this antitumor response was enhanced in the presence of SEB (EXO_{HSP70}/SEB)	No data	No data	No data	[38]

					enterotoxin B (SEB)							
**FB**					IL2/anti-IL2 mAb complexes (IL2c)<br><br>+/- anti-OX40 agonist		-anti-OX40/IL2 therapy boosted tumor regression to either treatment alone		-anti-OX40/IL2 therapy enhanced animal survival to either treatment alone	-IL2 treatment significantly enhanced CD25 and OX40 expression on CD8+ T cells in TME<br>-anti-OX40/IL2c therapy did not affect the Treg accumulation	[39]
**FB**					anti-OX40 agonist<br><br>+/- galectin-3 inhibitor (Gal-3, belapectin)		-significant reduction in tumor growth only in response to combined anti-OX40/belapectin treatment compared to monotherapies	No data	-belapectin combination enhanced the survival benefit produced following anti-OX40 treatment<br>-combination therapy generated durable long-term memory as all tumor-free mice were protected from subsequent tumor re-challenge	-no significant changes in the percent of CD8+, CD4+ T effectors or CD4+ T regs across treatment groups<br>-combined treatment reduced the proliferation of CD4+ Teff and Treg cells and increased IFNγ secretion in the CD8+ T cells<br>-anti-OX40/belapectin therapy decreases both number and immunosuppressive function of M-MDSCs	[40]
**FB**					anti-OX40 agonist<br><br>+/- caloric restriction (CR)/ resveratrol (RES)		-effect of tumor growth inhibition was stronger in CR mice treated with OX40 agonist<br>-combination with resveratrol does not boost anti-OX40-mediated tumor immunity in aged tumor-bearing mice	No data	-agonist OX40-mediated tumor free survival was greater in young and elderly calorically restricted mice compared to ad libitum controls<br>-long-term dietary supplementation with resveratrol did	-CR maintains tumor-antigen-specific CD4 T cell priming to levels similar to young mice, but not CD8+ T cell priming in the context of anti-OX40 treatment	[41]

									not significantly increase anti-OX40-mediated tumor free survival in aged mice		
**FB**					anti-CD40 agonist	sunitinib	-anti-CD40 therapy did not affect tumor growth while its combination with sunitinib induced a significant tumor regression	No data	-only anti-CD40+sunitinib combination prolonged overall survival	-anti-CD40 combined with sunitinib caused a greater increase in TDLNs activated CD11c+ DCs and intratumoral levels of MDSCs compared to anti-CD40 alone<br>-only upon combination therapy CD8+ tumor infiltration and VCAM-1 adhesion protein were increased	[42]
**FB**					CD122- IL2 pathway agonist (NKTR-214)	RT	-RT alone caused growth delay but did not affect systemic abscopal response<br>-NKTR-214 monotherapy cured bilateral disease and combination offered a significantly greater tumor control and	No data	-significant improvement in overall survival when animals received combination therapy as compared with controls or RT or NKTR-214 treatments	No data	[43]
**FB**					synthetic TLR4 agonist (Dendrophilin, DEN)	doxorubicin	-DEN enhanced the antitumor effect of doxorubicin<br>-DEN alone did not have any antitumor effect	No data	-DEN enhanced the survival benefit of doxorubicin	-DEN combined with doxorubicin promoted the accumulation of myelomonocytic and TH1/Tc1 infiltrates in tumor beds	[44]
**FB**					PSK (protein-bound polysaccharide K)	docetaxel	No data	- PSK and PSK plus docetaxel treatment suppressed metastasis formation of tumors with high MHC-I expression, while	No data	-immunotherapy and chemoimmunotherapy each produced a significant Increase in intratumoral CD4+ and CD8+ T-cell subsets and a decrease in Treg cells	[45]

								docetaxel alone had a moderate effect			
**DDLPS**	anti-PD-1						-PD-1 blockade slowed tumor growth compared to untreated control	No data	No data	-anti-PD-1 treatment induced the enhancement of CD8+ T (IFNγ+ and PD-1) cells, NK active abundance, IFNγ signature, cytolytic score, and CSF1 expression	[46]
**LPS**	anti-PD-1		viral ILP regimen (GLV-1h68, melphalan, TNFα)				-tumor volumes were significantly smaller in rats treated with viral ILP combined with PD-1 blockade and all animals were cured of local recurrent disease	-compartment-ectomy of primary tumors prevented local relapsing in combination therapy cohort, as compared to the viral ILP alone	-combination of viral ILP with PD-1 blockade significantly improved survival compared with viral ILP alone in the neoadjuvant model	-combination of viral ILP with PD-1 blockade significantly increased infiltration of CD8+ T cells in tumor parenchyma compared with the invasive margin, increased intratumoral CD3+ and expression of GZMB on T cells<br>-combination therapy increased the number of intratumoral DCs but also augmented their function	[47]
**RMS**		ERBB2-CAR cytokine-induced killer (CIK) cells					-ERBB2-CAR CIK cells led to a complete inhibition of initial tumor load, whereas WT CIKs had a moderate antitumor effect	-tumors were macroscopically cleared in all mice treated with ERBB2-CAR CIK cells, while small macroscopic lesions were observed in some of the mice treated with WT CIK cells	-all mice treated with ERBB2-CAR CIK cells displayed 100% survival	-ERBB2-CAR CIK cells enhanced the accumulation of NK and NKT cell subpopulations in disseminated RMS tumors, which was not observed for WT CIK cells	[48]
**RMS**		NKAE cell therapy			anti-CXCR4 (MDX1338)		-treatment with MDX1338 mAb alone led to the development of tumors slightly smaller than those of the control mice<br>-NKAE cell therapy alone was sufficient to prevent the intraperitoneal implantation of RH30 tumors entirely	-MDX1338 treatment alone significantly decreased the formation of micrometastases, while combination of MDX1338 and NKAE treatment	No data	No data	[49]

								completely suppressed metastasis			
**RMS**		fAChR-redirected T cells			survivin blockade (SHP)		-fAChR-specific, but not control, T cells delayed the growth of xenotransplants combination with SHP enhanced antitumor effect of ACT	No data	No data	-survivin blockade increased tumor susceptibility toward a T-cell attack, whereas induction of ICOS-L did not	[50]
**RMS**			VSVΔ51-GFP/ IFNγ/ TWEAK		LCL161 (SMAC mimetic)		-combination of LCL161 with VSVΔ51-GFP or IFNγ or TWEAK reduced RMS cell viability in vitro -LCL161 combined with VSVΔ51-GFP inhibited tumour growth in syngeneic mice while LCL161 or VSVΔ51-GFP alone did not	No data	-LCL161 combined with VSVΔ51-GFP extended overall survival of mice whereas LCL161 or VSVΔ51-GFP alone did not have any effect	-VSVΔ51-GFP or IFNγ or TWEAK synergized with LCL161 in vitro to promote TNFα signaling	[51]
**RMS**				DC-based tumor-cell vaccine	DASH inhibitor (ARI-4175)		-ARI-4175 monotherapy had potent antitumor activity but when combined with DC vaccine led to complete tumor regression	No data	-ARI-4175 monotherapy extends survival which was further enhanced upon combination with DC vaccine	-ARI-4175 increased splenic CD4+ T cells, NK cells, and DCs, but had no effect on B cells	[52]
**RMS**				DC vaccine/ ACT with tumor-primed T cells	DASH inhibitor (ARI-4175)		-ARI-4175 induced tumor regression and resulted in immunologic memory that protected against rechallenge -addition of DC vaccine or ACT to ARI-4175 improved the therapeutic efficacy -DC vaccine is sufficient to protect against tumor challenge, but it is insufficient as a therapeutic vaccine	No data	-combination of ARI-4175 with DC vaccine or ACT significantly enhanced survival as compared to ARI-4175 monotherapy	-anti-tumor effect of ARI-4715 correlated with a dose-dependent increase in recruitment of myeloid cells (macrophages) and DCs to secondary lymphoid tissues	[53]
**LMS**			Ad5/3-D24-GMCSF				-Ad5/3-D24 or Ad5/3-D24-GMCSF was more effective than the mock injection or	No data	No data	No data	[54]

							Ad5WT in reducing tumor growth				
**LMS**					anti-CD47		-reduction in tumor size	-decrease in the size and number of metastases	No data	No data	[55]
**SS**					α-particle emitting anti-Frizzled homolog 10 (FZD10) antibody (^{211}At-OTSA101) or antibody conjugated with the β-emitter yettrium-90 (^{90}Y-OTSA101)		-^{211}At-OTSA101 suppressed tumor growth immediately after injection, whereas this effect required several days in the case of ^{90}Y-OTSA101	No data	-both radiolabeled antibodies at the 50-μCi dosage level significantly prolonged survival	No data	[56]
**UPS**	anti-PD-1				RAR signaling antagonist (BMS493)		- BMS493 synergized with systemic anti-PD-1 therapy inducing a more potent tumor growth inhibition compared to monotherapy groups	No data	No data	-BMS493 treatment increases immunostimulatory TAM populations and CD4+ T cells	[6]
**UPS**		autologous CIK cells					-significant reduction of tumor growth in CIK treated mice compared with untreated controls	No data	No data	-significant reduction in residual putative chemoresistant cancer stem–like cells (sCSCs) at the end study after CIK treatment	[57]
**1956 sarcoma**	anti–PD-1				DDR2 inhibition (dasatinib)		-combination of anti-PD-1 and dasatinib led to a significant tumor load reduction<br>-therapeutic blockade of PD-1 or DDR2 alone had little effect	No data	No data	No data	[58]
**24JK-HER-2 sarcoma**		PTPN2-deficient CD8+ HER-2 CAR T cells					-effective at specifically killing 24JK-HER-2 cells but not 24JK cells in vitro	No data	No data	-PTPN2-deficient CD8+ HER-2 CAR T cells exhibited increased antigen-specific cytotoxic capacity in vitro after challenge with tumor cells (IFNγ, TNF, GZMB)	[59]

**F244 MCA sarcoma**					NF-κB inhibitor		-in vitro treatment of tumor cells with NF-κB inhibitor enhanced their killing by NK cells<br>-blocking NF-κB in the tumour cells led to immune rejection in vivo	No data	No data	-NF-κB inhibition upregulated the expression of NKG2D ligand H60a on tumor cells in vitro	[60]
**MCA304 sarcoma**				IL13Rα2-targeted DNA vaccine	IL13-PE		-IL13-PE combined with IL13Rα2 DNA vaccination significantly reduced tumor burden and prolonging survival	No data	-IL13-PE combined with IL13Rα2 DNA vaccination significantly prolonged survival	-combination therapy induced CTL activity against established tumors and exhibited increased IFNγ release in spleens<br>-greater density of CD4 and CD8+ cells and inflammatory cytokine production in tumor samples from the mice receiving combination therapy compared with control tumors<br>-decrease in immunosuppressive Tregs and MDSCs within the TME after IL13-PE + IL13Rα2 DNA vaccine combination	[61]

FB, fibrosarcoma; DDLP, dedifferentiated liposarcoma; LP, liposarcoma; RMS, rhabdomyosarcoma; LMS, leiomyosarcoma; SS, synovial sarcoma; UPS, undifferentiated pleomorphic sarcoma; CT, chemotherapy; RT, radiotherapy; ICIs, immune checkpoint inhibitors; ACT, adoptive cell therapy

Purple font in letters indicates the combination therapy

No data	Worsen outcome (vs control)	No change (vs control)	Similar improved outcome (monotherapy ≈ combination therapy)	Improved outcome of monotherapy (vs control)	Improved outcome of combination therapy (vs monotherapy)

Table S2: Bone Sarcoma

	Types of Immunotherapy					Other	Treatment output				Refs
	ICIs	ACT	Oncolytic viruses	Cancer Vaccines	In situ vaccines	CT/RT/ surgery	Primary tumor growth	Metastasis	Overall Survival	Immunophenotype	
**OS**	anti-PD-1						No data	-anti-PD-1 significantly reduced lung metastasis	No data	-anti-PD-1 induced NK and M1 macrophage infiltration into OS lung metastases	[62]
**OS**	anti-PD-1						-No change in the primary tumor volume after anti-PD-1	-fewer lung metastatic nodes in anti-PD-1 group	No data	No data	[63]
**OS**	anti-PD-1, anti-PD-L1, anti-OX-86 cocktail						No data	-immuno-cocktail suppressed the metastatic tumor proliferation	-immuno-cocktail prolonged overall survival compared to untreated group	No data	[64]
**OS**	anti-PD-1/ anti-PD-L1	T cells armed with anti-GD2-BsAb (GD2-EATs) or anti-HER2-BsAb (HER2-EATs)					-anti-PD-L1 combination improved tumor control compared to EATs alone, while anti-PD-1 combination did not	No data	-sequentially continuous therapy (SCT) with GD2-EATs-anti-PD-L1 combination improved OS while anti-PD-1 combination did not	-higher levels of IL2, TNFα and IFNγ after EATs injection<br>-PD-L1 expression was upregulated following BsAb treatment<br>-anti-PD-L1 combination resulted in more TILs compared to GD2-EATs or HER2-EATs alone, whereas anti-PD-1 combination did not	[65]
**OS**	anti-PD-1		OBP-502 (telomerase specific)				-OBP-502 combined with anti-PD-1 suppressed tumor volume while anti-PD-1 alone did not	No data	No data	-OBP-502 alone or in combination with anti-PD-1 increased CD8+ T cell infiltration in TME while anti-PD-1 alone did not	[66]
**OS**	anti-CTLA-4			tumor lysate-pulsed			-DC(Ly) combined with CTLA-4 blockade caused the strongest reduction in	-DC(Ly) combined with CTLA-4 blockade	No data	-DC(Ly) and anti-CTLA-4 treated mice had reduced numbers of Tregs and	[67]

				DCs [DC(Ly)]			size of metastatic lesions, while DC(Ly) or anti-CTLA-4 had a moderate antitumor effect	prolonged lifetime more effectively than either treatment alone		increased CD8+ T cells inside the metastatic tumor, while their combination further enhanced these effects<br>-serum IFNγ levels were increased, and IL10 decreased in the combination treatment group compared to those that received the DC(Ly) or anti-CTLA-4 antibody alone	
**OS**	anti-CTLA-4/<br>anti-PD-1			CD103+ cDC1s activated with poly toll-like receptor 3 agonist polyinosinic:polycytidylic acid (poly I:C)			-CD103+ cDC1 vaccination or CTLA-4 or DC vaccination combined with PD-1 blockade restrained tumor growth in some mice<br>-DC vaccination combined with anti-CTLA-4 led to a complete regression of tumors	-DC vaccination reduced in lung metastasis formation<br><br>(No data for combination therapy)	-CD103+ cDC1s vaccination prolonged survival<br>-combination with anti-CTLA-4 induced the most potent immunological memory responses against OS cell rechallenge	- vaccination enhanced IFNγ+ CD8+ T cell and Th1 infiltration<br>but also, increased Tregs compared to the poly I:C-stimulated MoDCs	[68]
**OS**	anti-PD-1				shFGD1		-shFGD1 and anti-PD-1 had similar effect in reducing tumor growth while combination caused the greatest reduction	No data	-shFGD1 combined with anti-PD-1 caused the longest survival time compared to monotherapies	-shFGD1 and anti-PD-1 monotherapies increased TILs and decreased myeloid cell infiltration, but combination enhanced these effects	[69]
**OS**	anti-PD-1				CXCR4 inhibitor (AMD3100)		-anti-PD-1 alone did not suppress growth<br>-CXCR4 inhibition slightly inhibited tumor growth and their combination has the strongest reduction in tumor size	No data	-CXCR4 inhibition prolonged mouse survival which was enhanced by CXCR4-PD-1 blockade combination	-CXCR4 inhibition alone and combination treatment increased CD8+ T cell infiltration<br>-Combination caused a further upregulation of Ki67, GZMB, IFNγ, and TNFα expression in CD8+ TILs, compared to monotherapies	[70]

										-CXCR4 inhibition alone and combination reduced MDSCs in a similar manner	
**OS**	anti-PD-L1				L-arginine		No data	No data	-anti-PD-L1 and L-arginine combination increased survival more effectively than anti-PD-L1 alone<br>-L-arginine had no effect on survival	-anti-PD-L1 and L-arginine combination elevated the number and activity of CD8+ T-cells in orthotopic tumors and pulmonary metastasis<br>-anti-PD-L1 alone increased activation of CD8+ T-cells in tumors and pulmonary metastasis and suppressed MDSC infiltration to orthotopic tumors	[71]
**OS**	anti-Tim-3/anti-PD-L1/anti-OX-86 cocktail					debulking surgery	No data	-lung metastases were abolished in the long-term survivors treated with combination therapy	-immunotherapy alone did not prolong survival, while combination with debulking surgery significantly extended it	No data	[72]
**OS**	anti-CTLA-4/ anti-PD-1					Carbon ion (CIRT) or photon (XRT) radiotherapy	-significant reduction in tumor growth after combining radiotherapy with ICI compared to ICI monotherapy<br>-reduction of abscopal tumors after ICI or XRT-ICI and CIRT-ICI combination	-CIRT-ICI and XRT-ICI reduced lung metastasis compared to ICI, XRT or CIRT monotherapies	No data	-increased CD8 infiltration in abscopal tumors after XRT-ICI and CIRT-ICI combination	[73]
**OS**	anti-PD-1					doxorubicin	-doxorubicin combined with an anti-PD-L1 reduce tumor growth more effectively than doxorubicin or anti-PD-1 monotherapy	No data	No data	-doxorubicin upregulated PD-L1 expression in osteosarcoma<br>CD4+ T cells, CD8+ T cells, and CTLs in the dox-treated group were higher, whereas the proportion of Tregs was lower compared to dox or anti-PD-1 monotherapy	[74]

**OS**	anti-PD-L1					sunitinib	-sunitinib and anti-PD-L1 combination inhibited tumor growth compared to monotherapies	-sunitinib and anti-PD-L1 suppressed lung metastasis compared to the control but combination had the strongest effect	-sunitinib and anti-PD-L1 combination prolonged mouse survival compared to monotherapies	-levels of CD8+T cells were higher and T regs lower in the sunitinib or anti-PD-L1 or combination treatment groups compared to the control group	[75]
**OS**	anti-PD-L1				ketotifen	doxorubicin	-ICI combined with doxorubicin or ketotifen reduced tumor volume<br>-ICI+ doxorubicin+ ketotifen had superior effect on growth inhibition	No data	No data	-ICI combined with doxorubicin or ketotifen or both increased intratumoral levels of CD8+ T cells	[12]
**OS**	anti-PD-L1				dexamethasone	Doxorubicin or Epirubicin (NC6300)	-Both anthracycline combinations with ICI resulted in tumor regression compared to anti-PD-L1 monotherapy<br>-ICI+NC6300+ dexamethasone caused a greater reduction in tumor growth than ICI+NC6300 combination	No data	No data	No data	[13]
**OS**		AD-MSCs as mbTRAIL cellular vectors					-MSC-TRAIL induced apoptosis of OS cells by a direct cell-to-cell contact, but in vivo MSC-TRAIL treatment increased tumor development	-MSC-GFP/OS cell co-injection increased pulmonary metastasis	-MSC-GFP/OS cell co-injection decreased mouse survival	No data	[76]
**OS**		B7-H3 CAR T cells					-B7-H3 CAR T cells eradicated osteosarcoma tumors in vivo	No data	-B7-H3 CAR T cell therapy led to a significant survival advantage compared with control CAR T-cell–treated mice	No data	[77]

**OS**		CD166.BBζ CAR-T cells					-CD166.BBζ CAR-T cells efficiently suppressed tumor growth with no off-target toxicity	No data	No data	No data	[78]
**OS**		NKG2D-CAR+ CD45RA- T cells					-NKG2D CAR-redirected CD45RA− cells lysed osteosarcoma cells in vitro and in vivo	No data	-mice treated with NKG2D CAR-redirected CD45RA− cells exhibited prolonged survival after rechallenge with OS cells	No data	[79]
**OS**		IGF1R and ROR1 CAR T cells					-IGF1R and ROR1 CAR T cells derived suppressed sarcoma growth in vivo	No data	-both CAR T cells extended overall survival compared to the untreated group	-produced high amounts of IL13 Th2 cytokine but not IL4 and IL10 in vitro	[80]
**OS**		HER2-specific T cells					-decrease in the sarcosphere forming efficiency from tumors treated with HER2-specific T cells<br>No effect on tumor size in vivo	No data	No data	No data	[81]
**OS**		GD2-CAR.OX40.28. ζ CAR T cells			all-trans retinoic acid (ATRA)		-GD2-CAR T cells showed minimal antitumor effect against OS in vivo<br>-GD2-CAR T cells combined with ATRA reduced tumor volume	No data	-GD2-CAR T cell therapy alone did not improve survival<br>-GD2-CAR T cells combined with ATRA prolonged survival	-ATRA treatment led to a significant granulocytic reduction in MDSCs compared to untreated tumors<br>-GD2-CAR T cells combined with ATRA increased peripheral CD8+ T cell population	[82]
**OS**		γδ T cells			decitabine (DAC, DNA demethylation drug)		-DAC or γδ T cells alone slightly decreased tumor growth, whereas γδ T cells + DAC treatment caused a substantial decrease	No data	No data	-upregulation of NKG2DLs MICB and ULBP1 in DAC-pretreated OS cells compared to the non-DAC treatment group	[83]
**OS**		NK cell therapy			aerosol IL2		No data	-IL2 combined with NK cell	No data	-increased infiltration, retention, and proliferation of	[84]

								therapy significantly decreased lung metastasis compared to either monotherapy		IL12 infused NK cells in the lungs	
**OS**		autologous hematopoietic stem cell transplantation (HSCT)			Intratumoral IFNβ gene transfer		-IFNβ gene transfer in combination with syngeneic HSCT suppresses tumor growth more effectively compared to IFNβ gene transfer alone	-combination therapy inhibited formation of spontaneous lung and liver metastases	No data	-combination therapy increased the infiltration of many immune cells in metastatic tumors	[85]
**OS**		NK cell therapy			IL2 injections	spironolactone (SPIR)	-groups treated with NKAE cells +IL2 and/or SPIR had less bone damage, smaller tumor volumes compared to mice treated with SPIR alone<br>-NKAE cells +IL2+SPIR combination had the strongest antitumor effect	-mice treated with NKcells showed no lung metastasis while SPIR and control groups do	-NKAE cells +IL2+SPIR treated mice survived longer compared to NKAE cells +IL2 treatment	-tumor cell co-culture with NKAE cells reduced CXCR4 and c-kit positive subsets	[86]
**OS**			rhabdovirus MG1				-MG1 treatment slowed tumor progression	No data	-MG1 treatment significantly increased the number of cured mice and induced the generation of memory immune response that provided protection against a subsequent tumor challenge	No data	[87]
**OS**				TRXtr-mCD99			-vaccination resulted in suppressed tumor growth	No data	No data	-production of anti-mCD99 antibodies upon vaccination and reduction in tumor	[88]

				vaccination			compared to the control vaccinated mice (TRXtr)			microvessel density and functionality	
**OS**				allograft DC-osteosarcoma fusion (DOF) tumor vaccine			-tumor bodies after DOF treatment showed atrophy or even disappeared	No data	-DOF treatment led to longer survival	-DOFs increased CD8+ cell percentage while that of CD4+ cells decreased compared to untreated DCs<br>-Killing potential of DOF-T cells was significantly enhanced	[89]
**OS**				Allogeneic OS mRNA–DC electrotransfection			-rats immunized with allogeneic mRNA–DCs rejected tumor challenge	No data	-preimmunization with allogeneic DC–tumor mRNA electrotransfected vaccine extended survival the most compared to immunization with DCs, UMR108-derived mRNA, an unelectrotransfected mixture of the two Survivors developed long immunological memory and rejected subsequent rechallenge	-T cells stimulated with allogeneic mRNA–DC electrotransfection product exhibited antitumor CTL responses	[90]
**OS**				anti-A5 sera or anti-PCMT1 sera vaccination			-sarcoma was significantly inhibited by vaccination with A5	No data	No data	-antibodies induced by A5 or PCMT1 mediated their antitumor roles by eliciting strong antibody-dependent cellular cytotoxicity and complement-dependent cytotoxicity toward tumor cells	[91]

**OS**				neem leaf glycoprotein (NLGP)-vaccine			No data	No data	No data	-NLGP matured DCs<br>-CD4+ T cell help was not required to generate CD8+ T cells but to maintain CD8+ T cells antitumor activity by physical contact and partial involvement of IL2 in CD4	[92]
**OS**				mHSP/Ps vaccine			-mice immunized with mHSP/Ps showed tumor regression	No data	-mice immunized with mHSP/Ps showed long-term survival<br>-long term memory since rehallenging of mice did not led to tumor development	-NK cells, CD8+, and IFNγ-secreting cells and tumor-specific cytotoxic T-cell activity were increased in the immunized group	[25]
**OS**				DC(Ly) vaccine	anti-TGFβ mAb (1D11)		No data	-DC(Ly) + anti-TGF-β combination more effectively reduced the volume of lung metastasis	No data	-TGFβ inhibition caused a moderate CD8+ T cell infiltration in pulmonary metastasis which was enhanced upon combination with DC(Ly) vaccination, while both treatments caused a similar significant reduction in Tregs<br>-mice treated with combination therapy had the highest IFNγ levels and the lowest IL10 levels	[93]
**OS**				DC(Ly) vaccine	anti-GITR agonist		-combination of DC(Ly) + anti-GITR induced the most potent reduction in tumor growth compared to the DC(Ly) alone or anti-GITR which moderately decrease tumor size	No data	-combination treatment significantly prolonged survival<br>-survival benefit of DC(Ly) alone and anti-GITR alone was small compared to control	-anti-GITR combined with DC (Ly) reduced intratumoral Tregs and increased CD8+ T cells compared to anti-GITR monotherapy<br>-serum levels of IFNγ were higher while IL10 levels lower in the combination treatment group	[94]

**OS**					anti-CD47 mAb		No data	-CD47 blockade inhibited invasive ability OS cells in vitro and spontaneous metastasis in vivo	No data	-CD47 blockade increased macrophage phagocytosis of OS cells	[95]
**OS**					anti-CXCR4 (12G5)		-CXCR4-blocking antibodies diminished osteolysis	-inhibition of lung micro-metastasis	No data	-blocking inhibition of pAKT of OS cell and migration in vitro	[96]
**OS**					BsAbBmi/TRIM		-BsAbBmi/TRIM-treated mice had the smallest tumor growth compared to AbBmi-1 or AbTRIM-14 treatment which had a comparable effect on tumor size	No data	-BsAbBmi/ TRIM-treated mice exhibited extended survival compared to AbBmi-1 or AbTRIM-14 treatment	-BsAbBmi/TRIM-treated tumors had the lowest proliferation, highest apoptotic index and lowest microvascular density BsAbBmi/TRIM blocks expression of MMP9 by NF-κB signaling inhibition	[97]
**OS**					radioimmunotherapy (RIT) with radiolabeled anti-IGF2R mAb (2G11) with 213Bi and 177Lu		-213Bi-2G11 and 177Lu -2G11 slowed down the growth	No data	No data	No data	[98]
**OS**					188Re-labeled IGF2R-specific mAb		-treatment with 188Re-labeled IGF2R-specific mAb suppressed tumor growth	No data	No data	No data	[99]
**OS**					anti-CD47 mAb		-anti-CD47 therapy reduced tumor burden	No data	No data	-anti-CD47 inhibition induced phagocytosis of both tumor	[100]
**OS**					shDNMT1 decitabine (DNA hypomethylation agent)		No data	-hypomethylating treatment with decitabine prevented the formation of spontaneous lung metastases	-increased overall survival	-shDNMT1 or decitabine significantly increased the number of intrapulmonary CD8+ T cells<br>-only decitabine promoted expression of the level of IFNγ and TNFα in CD8+ T cells and proliferation<br>-increased CXCL12, CXCL9 and CXCL10 chemokines	[101]

**OS**					LPS (or MPL)		-LPS induced TLR-4 stimulation decreased tumor volume	-mice induced with LPS-induced TLR4 stimulation had significantly lower rates of lung metastasis	-LPS induced TLR-4 activation had extended overall survival	-LPS induced TLR-4 stimulation increasing the levels of TNFα and IFNγ in serum and spleen<br>-increased infiltration of CD8+ T cells but not F4/80+ TAMs in tumor and lung metastasis	[102]
**OS**					DCs transfected with miR-133a inhibitor		-DCs injection with miR-133a inhibitor dramatically decreased the volume of tumor	No data	-survival rate of mice was significantly improved by DCs transfected with miR-133a inhibitor treatment	-miR-133a suppression in DCs promoted maturation and activation of DCs via RBP-J<br>-miR-133a inhibition contributed to the elevation of CD8+ T cells<br>-miR-133a inhibitor stimulated Notch signaling (Hes1 and Hey1)	[103]
**OS**					pCMV-immunocasp (fusion of a HER2-specific single-chain Ab, a single-chain Pseudomonas exotoxin A and an active caspase-6)		-immunocasp-6 induced apoptosis of the HER2-overexpressing OS cells and suppresses tumor growth in vivo	-immunocasp-6 inhibited lung metastasis of HER2-overexpressing OS tumors	immunocasp-6 increased overall survival HER2-overexpressing OS tumors compared to the untreated group	No data	[104]
**OS**					hemocyanin		-tumor weight and volume were decreased significantly in hemocyanin treated groups	No data	No data	-hemocyanin treatment led to higher NK cell activity, enhanced the ability of ConA to induce the proliferation of T cells, stimulated the secretion of TNFα	[105]
**OS**					PS-F2, a polysaccharide		-administration of PS-F2 effectively suppressed the growth of sarcoma tumors	No data	No data	-CD4+ T cells, CD8+ T cells, and serum from PS-F2-treated tumor-bearing mice all exhibited antitumor activities when adoptively transferred to naïve animals	[106]
**OS**					Lachnum polysaccharide (LEP)		-LEP induced a strong tumor growth inhibition	No data	No data	-LEP decresed microvascular density of tumors and resets TAMs from protumor M2 to antitumor M1	[107]

										phenotype by promoting Th1 polarization and IFNγ secretion<br>-LEP might directly activate M1 macrophages via TLR4 mediated NF-κB signaling pathway<br>-LEP decreased the infiltration of immunosuppressive MDSCs and Tregs	
**OS**					lipid-coated calcium zoledronate nanoparticles (CaZol@pMNPs) containing conjugated mannose		-CaZol@pMNPs restrained tumor growth without	No data	No data	-CaZol@pMNPs effectively depleted TAMs, markedly decreased angiogenesis MMP-9 and IL10 expression	[108]
**OS**					IAPS-2 polysaccharide		-tumor was significantly decreased upon IAPS-2 treatment	No data	-IAPS-2 treatment extended animal survival	-IAPS-2 polysaccharide promoted the secretion of antitumor cytokines (IL12, IFNγ) in TAMs and increased the expression of genes associated with M1 type (NOS2 and MHC II) by the activation of NF-κB and STAT signaling	[109]
**OS**					Helicobacter pylori neutrophil activating protein fused with the maltose binding protein (rMBP-NAP)		-rMBP-NAP administration decreased tumor growth compared to the control group	No data	No data	- rMBP-NAP treatment induced a significant accumulation of splenic CD4+ IFNγ-secreting cells, which is a cytokine profile of Th1 cells	[110]
**OS**					Methionine enkephalin (MENK) neuropeptide		-MENK delayed the development of tumor	No data	No data	-MENK effectively down-regulated level of Tregs by suppressing the expression of Foxp3 induced by TGFβ via weakening the phosphorylation and nuclear translocation of Smad2/3	[111]

**OS**					Methionine enkephalin (MENK) neuropeptide		-ACT of CD8+T cells after treatment with MENK led to a significant shrinkage in tumor growth	No data	- ACT of CD8+T cells after treatment with MENK result in significantly increased survival	-MENK administration increased the CD8+ T cells in spleen and lymph nodes and the expression of CTLA-4, CD28, FasL and GrzB on CD8+ T cells<br>-MENK led to increased expression of opioid receptors on CD8+ T cells which is essential for the activation of CTL<br>-MENK-induced T cell signaling is associated with a significant up-regulation of Ca^{2+} influx into the cytoplasm and the translocation of NFAT2 into nucleus	[112]
**OS**					cationic polymers [e.g., polyethyleneimine (PEI), C-dextran]		-cationic polymer administration reduced tumor volume	No data	-prolonged the survival	-increased the expression of pro-inflammatory genes (NOS2 and MHCII) and reduced expression of M2-specific genes (Arg1 and Ym1) through TLR-4 signaling<br>-promoted the production of the antitumor cytokine IL12 and reduced the expression levels of IL 10, and promoted Th 1 and NK cell infiltration, while suppressing tumor angiogenesis	[113]
**OS**					neem leaf glycoprotein (NLGP)		-adoptive transfer of NLGP-TME exposed T cells, but not PBS-TME exposed cells in mice, significantly inhibited sarcoma tumor growth	No data	No data	-NLGP switched the IL10, TGFβ, IL6 rich type 2 characters to type 1 microenvironment with dominance of IFNγ, IL2 and IL12 secretion<br>-NLGP normalized protumor angiogenic and hypoxic TME<br>-significant upregulation in perforin and GZMB in CD8+ T cells and reduction	[114]

										recruitment of MDSCs and Tregs<br>- NLGP protects CD8+ T cells from anergy within TME	
**OS**					neem leaf glycoprotein (NLGP)/ cyclophosphamide (CTX)		-tumor growth rate was faster in the CTX cohort than the NLGP treated mice	No data	-NLGP treated mice had longer survival compared to CTX or untreated control	-NLGP generated superior tumor specific central memory CD8+ T cells than CTX that averted post-surgery solid sarcoma recurrence<br>-tumor surgery decreased antigen load and restored functionality of memory T cell by activation of Wnt signalling with concomitant inhibition of GSK-3β and stabilisation of β-catenin	[115]
**OS**					cationic polymers [e.g., polyethyleneimine (PEI), C-dextran]		-cationic polymer administration reduced tumor volume	No data	-prolonged the survival	-increased the expression of pro-inflammatory genes (NOS2 and MHCII) and reduced expression of M2-specific genes (Arg1 and Ym1) through TLR-4 signaling<br>-promoted the production of the antitumor cytokine IL12 and reduced the expression levels of IL 10, and promoted Th 1 and NK cell infiltration, while suppressing tumor angiogenesis	[113]
**OS**					neem leaf glycoprotein (NLGP)		-adoptive transfer of NLGP-TME exposed T cells, but not PBS-TME exposed cells in mice, significantly inhibited sarcoma tumor growth	No data	No data	-NLGP switched the IL10, TGFβ, IL6 rich type 2 characters to type 1 microenvironment with dominance of IFNγ, IL2 and IL12 secretion<br>-NLGP normalized protumor angiogenic and hypoxic TME<br>-significant upregulation in perforin and GZMB in CD8+ T cells and reduction recruitment of MDSCs and Tregs	[114]

										- NLGP protects CD8+ T cells from anergy within TME	
**OS**					neem leaf glycoprotein (NLGP)/ cyclophosphamide (CTX)		-tumor growth rate was faster in the CTX cohort than the NLGP treated mice	No data	-NLGP treated mice had longer survival compared to CTX or untreated control	-NLGP generated superior tumor specific central memory CD8+ T cells than CTX that averted post-surgery solid sarcoma recurrence<br>-tumor surgery decreased antigen load and restored functionality of memory T cell by activation of Wnt signalling with concomitant inhibition of GSK-3β and stabilisation of β-catenin	[115]
**OS**					TLR7/8 agonist (R848)	doxorubicin (DOX) and cisplatin (CDDP) in NP formulation (CDDPNPDOX&R848)	-CDDPNPDOX+R848 induced the greatest reduction in tumor growth compared to NPR848	No data	-CDDPNPDOX + R848 induced the greatest increased in survival compared to NPR848<br>-CDDPNPDOX & R848 caused a long-term memory response	-combination therapy induced DCs maturation, promoted TAM polarization from M2 to M, increased infiltration of CD8+ T cells and secretion of IFNγ and IL6 compared with NPR848	[116]
**OS**					IL2/anti-S4B6	RT	-IL2/S4B6 monotherapy had no significant tumor growth inhibition<br>-combined therapy and IR alone cause a similar reduction in tumor volume<br>-only combination therapy had an abscopal effect	No data	-only combination therapy significantly prolonged overall survival	-RT increased CD8+ T cells in irradiated tumors while combination in both irradiated and unirradiated tumors	[117]
**OS**					IL23 neutralizing antibody (16E5)	doxorubicin	-IL23 inhibition moderately reduced tumor growth while this effect	No data	-IL23 inhibition prolonged survival	-GRM4 suppresses cAMP production by DCs and subsequent IL23 expression	[118]

					or GRM4 agonist		was enhanced upon combination with doxorubicin<br>-treatment with GRM4 agonist alone significantly suppressed tumor growth with a potency comparable to doxorubicin			-IL23 inhibition increased T-cell cytotoxicity	
**OS**					shMIF (macrophage migration inhibitory factor)	cisplatin or doxorubicin	-MIF knockdown enhanced OS cell sensitivity to cisplatin and doxorubicin in vitro by suppressing the RAS/MAPK pathway activation<br>MIF silencing inhibited tumor growth in vivo	-MIF knock down inhibited lung metastasis	No data	No data	[119]
**EwS**		B7-H3 CAR T cells					-B7-H3 CAR T cells eradicated Ewing sarcoma tumors in vivo	No data	-B7-H3 CAR T cell therapy led to a significant survival advantage compared with control CAR T-cell–treated mice	No data	[77]
**EwS**		VEGFR2-specific CAR T cells					-VEGFR2-specific CAR T cells specifically lysed VEGFR2-expressing target cells and spheroids	No data	No data	No data	[120]
**EwS**		GD2-specific CAR- T cell therapy					-no difference in primary tumor growth between treated and untreated mice	-mice treated with ACT had a growth delay of lung tumors, with both lower numbers and smaller volumes	-no overall survival advantage was found for mice receiving T-cell therapy	No data	[121]
**EwS**		CHM1-specific TCR-transgenic T cells					No data	-CHM1-specific TCR-transgenic T cells inhibited the formation of lung and liver	No data	-CHM1-specific TCR-transgenic T cells increased CD8+ T cell infiltration in the metastatic sites	[122]

								metastases in contrast to control mice			
**EwS**		mbIL15-NK cell therapy					-mice receiving mbIL15-transduced NK cells had a slower tumor growth than untreated mice and mice treated with mock-transduced NK cells	No data	-mice treated with mbIL15-NK cell therapy had a significantly longer survival	-mbIL15-NK cells expanded over time in all tissues whereas mock-transduced NK cells did not	[123]
**EwS**		mbIL21-NK cells					-mbIL21-NK administration reduced the growth of the primary sarcoma tumor	-mbIL21-NK administration led to a dramatic reduction in tumor metastases to the lung	No data	-mbIL21 signaling promoted sustained NK cell expansion and increased metabolic activity	[124]
**EwS**		EWS-FLI1 (YLNPSVDSV)-specific CTL					No data	No data	-ACT of CTL specific for the modified peptide YLNPSVDSV resulted in enhanced survival of mice with established ES xenografts	-peptide modification increased stabilization of HLA-A2.1 molecules on the cell surface	[125]
**EwS**		NK cells or PBMCs	oncolytic measles vaccine virotherapeutics (MeV)				-co-culture of MeV and NK cells or PBMCs resulted in higher oncolysis rates than MeV monotherapy in sarcoma cells	No data	No data	-co-culture with NK cells + oncolytic virus increased the release of GZMA/B, perforin and granulysin<br>-higher levels of IFNγ and sFasL upon combination compared to NKAEs alone	[15]
**EwS**		GD2-specific CAR- T cell therapy			anti-HGF (AMG102) mAb		-AMG102 treatment only moderately decreased tumor burden in the bone<br>-CAR-T therapy did not affect primary tumor growth<br>-antitumor response was enhanced after combination with CAR-T therapy	-AMG102 treatment only moderately decreased tumor burden in the lungs	-AMG102 treatment increased survival compared to untreated mice<br>-CAR-T therapy had no effect<br>-combination treatment significantly	-higher accumulation of CD3+ and CD8+ T cells in CAR-T + AMG102 treatment group	[126]

									extended survival		
**EwS**		GD2-CAR.OX40.28. ζ CAR T cells			all-trans retinoic acid (ATRA)		-GD2-CAR T cells showed minimal antitumor effect against EWs in vivo<br>-GD2-CAR T cells combined with ATRA reduced tumor volume	No data	-GD2-CAR T cell therapy had a moderate effect on survival which was enhanced upon combination with ATRA	-ATRA treatment led to a significant granulocytic reduction in MDSCs compared to untreated tumors<br>-GD2-CAR T cells combined with ATRA increased peripheral CD8+ T cell population	[82]
**CHS**		Vγ9Vδ2 T cells			zoledronate (ZOL)		-treatment with ZOL alone did not inhibit tumor growth<br>-combination therapy of ZOL and Vγ9Vδ2 T cells led to tumor volume reduction compared to untreated tumors and improved efficiency of systemic immunotherapy	No data	No data	-Vγ9Vδ2 T cell-mediated cytotoxicity was mainly mediated by the perforin pathway	[127]

OS, osteosarcoma; EwS, Ewing sarcoma; CHS, chondrosarcoma; CT, chemotherapy; RT, radiotherapy; ICIs, immune checkpoint inhibitors; ACT, adoptive cell therapy
Purple font in letters indicates the combination therapy

References

1. Noguchi T, Ward JP, Gubin MM, et al. Temporally distinct PD-L1 expression by tumor and host cells contributes to immune escape. Cancer Immunol Res. 2017; 5: 106-117.

2. Gubin MM, Zhang X, Schuster H, et al. Checkpoint blockade cancer immunotherapy targets tumour-specific mutant antigens. Nature. 2014; 515: 577-581.

3. Ebrahimi-Nik H, Corwin WL, Shcheglova T, et al. CD11c MHCII lo GM-CSF-bone marrow-derived dendritic cells act as antigen donor cells and as antigen presenting cells in neoepitope-elicited tumor immunity against a mouse fibrosarcoma. Cancer Immunol Immunother. 2018; 67: 1449-1459.

4. Molgora M, Esaulova E, Vermi W, et al. TREM2 modulation remodels the tumor myeloid landscape enhancing anti-PD-1 immunotherapy. Cell. 2020; 182: 886-900.

5. Redmond WL, Linch SN, Kasiewicz MJ. Combined targeting of costimulatory (OX40) and coinhibitory (CTLA-4) pathways elicits potent effector T cells capable of driving robust antitumor immunity. Cancer Immunol Res. 2014; 2: 142-153.

6. Devalaraja S, To TKJ, Folkert IW, et al. Tumor-derived retinoic acid regulates intratumoral monocyte differentiation to promote immune suppression. Cell. 2020; 180: 1098-1114.

7. Nafia I, Toulmonde M, Bortolotto D, et al. IDO targeting in sarcoma: biological and clinical implications. Front Immunol. 2020; 11: 274.

8. Travelli C, Consonni FM, Sangaletti S, et al. Nicotinamide phosphoribosyltransferase acts as a metabolic gate for mobilization of myeloid-derived suppressor cells. Cancer Res. 2019; 79: 1938-1951.

9. Hurst KE, Lawrence KA, Essman MT, et al. Endoplasmic reticulum stress contributes to mitochondrial exhaustion of CD8 T cells. Cancer Immunol Res. 2019; 7: 476-486.

10. Puca E, Probst P, Stringhini M, et al. The antibody-based delivery of interleukin-12 to solid tumors boosts NK and CD8 T cell activity and synergizes with immune checkpoint inhibitors. Int J Cancer. 2020; 146: 2518-2530.

11. Corbellari R, Nadal L, Villa A, et al. The immunocytokine L19-TNF eradicates sarcomas in combination with chemotherapy agents or with immune check-point inhibitors. Anticancer Drugs. 2020; 31: 799-805.

12. Panagi M, Fotios M, Voutouri C, et al. Targeting mast cells restores T cell infiltration and sensitizes sarcomas to PD-L1 inhibition. (under revision).

13. Mpekris F, Panagi M, Michael C, et al. Translational nanomedicine regimen potentiates immune checkpoint inhibition in metastatic sarcoma by normalizing the microenvironment. (under revision).

14. Hosoi H, Ikeda H, Imai N, et al. Stimulation through very late antigen-4 and-5 improves the multifunctionality and memory formation of CD8 T cells. Eur J Immunol. 2014; 44: 1747-1758.

15. Klose C, Berchtold S, Schmidt M, et al. Biological treatment of pediatric sarcomas by combined virotherapy and NK cell therapy. BMC Cancer. 2019; 19: 1-15.

16. Kjaergaard J, Hatfield S, Jones G, et al. A2A adenosine receptor gene deletion or synthetic A2A antagonist liberate tumor-reactive CD8 T cells from tumor-induced immunosuppression. J Immunol. 2018; 201: 782-791.

17. Soto-Pantoja DR, Terabe M, Ghosh A, et al. CD47 in the tumor microenvironment limits cooperation between antitumor T-cell immunity and radiotherapy. Cancer Res. 2014; 74: 6771-6783.

18. Arab S, Motamedi M, Hadjati J. Effects of dendritic cell vaccine activated with components of Lieshmania major on tumor specific response. Iran J Immunol. 2019; 16: 268-277.

19. Huijbers EJ, van Beijnum JR, Lê CT, et al. An improved conjugate vaccine technology; induction of antibody responses to the tumor vasculature. Vaccine. 2018; 36: 3054-3060.

20. Koido S, Ito M, Sagawa Y, et al. Vaccination with vascular progenitor cells derived from induced pluripotent stem cells elicits antitumor immunity targeting vascular and tumor cells. Cancer Immunol Immunother. 2014; 63: 459-468.

21. Li X, Wang Y, Zhao Y, et al. Immunotherapy of tumor with vaccine based on basic fibroblast growth factor-activated fibroblasts. J Cancer Res Clin Oncol. 2014; 140: 271-280.

22. Chen L, Taylor JL, Sabins NC, et al. Extranodal induction of therapeutic immunity in the tumor microenvironment after intratumoral delivery of Tbet gene-modified dendritic cells. Cancer Gene Ther. 2013; 20: 469-477.

23. Huang C, Ramakrishnan R, Trkulja M, et al. Therapeutic effect of intratumoral administration of DCs with conditional expression of combination of different cytokines. Cancer Immunol Immunother. 2012; 61: 573-579.

24. Azadmehr A, Pourfathollah AA, Amirghofran Z, et al. Immunotherapy with tumor cell lysate-pulsed CD8α dendritic cells modulates intra-tumor and spleen lymphocyte subpopulations. Neoplasma. 2013; 60: 525-532.

25. Wang Y, Liu S, Yuan M, et al. Prophylactic antitumor effect of mixed heat shock proteins/peptides in mouse sarcoma. Chin Med J. 2015; 128: 2234.

26. Takamura-Ishii M, Miura T, Nakaya T, et al. Induction of antitumor response to fibrosarcoma by Newcastle disease virus-infected tumor vaccine. Med Oncol. 2017; 34: 1-8.

27. Khalili A, Muhammad Hassan Z, Shahabi S, et al. Long acting propranolol and HSP-70 rich tumor lysate reduce tumor growth and enhance immune response against fibrosarcoma in Balb/c mice. Iran J Immunol. 2013; 10: 70-82.

28. Rao A, Taylor JL, Chi-Sabins N, et al. Combination therapy with HSP90 inhibitor 17-DMAG reconditions the tumor microenvironment to improve recruitment of therapeutic T cells. Cancer Res. 2012; 72: 3196-3206.

29. Whelan MC, Casey G, Larkin JO, et al. Oral tolerance to cancer can be abrogated by T regulatory cell inhibition. PLoS One. 2014; 9: e97602.

30. Gasparri AM, Sacchi A, Basso V, et al. Boosting interleukin-12 antitumor activity and synergism with immunotherapy by targeted delivery with isoDGR-tagged nanogold. Small. 2019; 15: 1903462.

31. Balza E, Zanellato S, Poggi A, et al. The therapeutic T-cell response induced by tumor delivery of TNF and melphalan is dependent on early triggering of natural killer and dendritic cells. Eur J Immunol. 2017; 47: 743-753.

32. Razi Soofiyani S, Kazemi T, Lotfipour F, et al. Gene therapy with IL-12 induced enhanced anti-tumor activity in fibrosarcoma mouse model. Artif Cells Nanomed Biotechnol. 2016; 44: 1988-1993.

33. Hess C, Neri D. The antibody-mediated targeted delivery of interleukin-13 to syngeneic murine tumors mediates a potent anticancer activity. Cancer Immunol Immunother. 2015; 64: 635-644.

34. Gao Y, Souza-Fonseca-Guimaraes F, Bald T, et al. Tumor immunoevasion by the conversion of effector NK cells into type 1 innate lymphoid cells. Nat Immunol. 2017; 18: 1004-1015.

35. Premkumar K, Shankar BS. TGF-βR inhibitor SB431542 restores immune suppression induced by regulatory B–T cell axis and decreases tumour burden in murine fibrosarcoma. Cancer Immunol Immunother. 2021; 70: 153-168.

36. Vlková V, Štěpánek I, Hrušková V, et al. Epigenetic regulations in the IFNγ signalling pathway: IFNγ-mediated MHC class I upregulation on tumour cells is associated with DNA demethylation of antigen-presenting machinery genes. Oncotarget. 2014; 5: 6923–6935.

37. Aoki R, Iijima H, Kato M, et al. Protein-bound polysaccharide-K reduces the proportion of regulatory T cells in vitro and in vivo. Oncol Rep. 2014; 31: 50-56.

38. Behzadi E, Hosseini HM, Halabian R, et al. Macrophage cell-derived exosomes/staphylococcal enterotoxin B against fibrosarcoma tumor. Microb Pathog. 2017; 111: 132-138.

39. Redmond WL, Triplett T, Floyd K, et al. Dual anti-OX40/IL-2 therapy augments tumor immunotherapy via IL-2R-mediated regulation of OX40 expression. PloS one. 2012; 7: e34467.

40. Sturgill ER, Rolig AS, Linch SN, et al. Galectin-3 inhibition with belapectin combined with anti-OX40 therapy reprograms the tumor microenvironment to favor anti-tumor immunity. Oncoimmunology. 2021; 10: 1892265.

41. Farazi M, Nguyen J, Goldufsky J, et al. Caloric restriction maintains OX40 agonist-mediated tumor immunity and CD4 T cell priming during aging. Cancer Immunol Immunother. 2014; 63: 615-626.

42. van Hooren L, Georganaki M, Huang H, et al. Sunitinib enhances the antitumor responses of agonistic CD40-antibody by reducing MDSCs and synergistically improving endothelial activation and T-cell recruitment. Oncotarget. 2016; 7: 50277- 50289.

43. Walker JM, Rolig AS, Charych DH, et al. NKTR-214 immunotherapy synergizes with radiotherapy to stimulate systemic CD8 T cell responses capable of curing multi-focal cancer. J Immunother Cancer. 2020; 8: e000464.

44. Yamazaki T, Hannani D, Poirier-Colame V, et al. Defective immunogenic cell death of HMGB1-deficient tumors: compensatory therapy with TLR4 agonists. Cell Death Differ. 2014; 21: 69-78.

45. Romero I, Garrido C, Algarra I, et al. MHC intratumoral heterogeneity may predict cancer progression and response to immunotherapy. Front Immunol. 2018; 9: 102.

46. Choi B, Lee JS, Kim SJ, et al. Anti-tumor effects of anti-PD-1 antibody, pembrolizumab, in humanized NSG PDX mice xenografted with dedifferentiated liposarcoma. Cancer Lett. 2020; 478: 56-69.

47. Smith HG, Mansfield D, Roulstone V, et al. PD-1 blockade following isolated limb perfusion with vaccinia virus prevents local and distant relapse of soft-tissue sarcoma. Clin Cancer Res. 2019; 25: 3443-3454.

48. Merker M, Wagner J, Kreyenberg H, et al. ERBB2-CAR-engineered cytokine-induced killer cells exhibit both CAR-mediated and innate immunity against high-risk rhabdomyosarcoma. Front Immunol. 2020; 11: 2483.

49. Vela M, Bueno D, González-Navarro P, et al. Anti-CXCR4 antibody combined with activated and expanded Natural Killer cells for sarcoma immunotherapy. Front Immunol. 2019; 10: 1814.

50. Simon-Keller K, Paschen A, Hombach AA, et al. Survivin blockade sensitizes rhabdomyosarcoma cells for lysis by fetal acetylcholine receptor–redirected T cells. Am J Pathol. 2013; 182: 2121-2131.

51. Dobson CC, Naing T, Beug ST, et al. Oncolytic virus synergizes with Smac mimetic compounds to induce rhabdomyosarcoma cell death in a syngeneic murine model. Oncotarget. 2017; 8: 3495-3508.

52. Donahue RN, Duncan BB, Fry TJ, et al. A pan inhibitor of DASH family enzymes induces immunogenic modulation and sensitizes murine and human carcinoma cells to antigen-specific cytotoxic T lymphocyte killing: implications for combination therapy with cancer vaccines. Vaccine. 2014; 32: 3223-3231.

53. Duncan BB, Highfill SL, Qin H, et al. A pan-inhibitor of DASH family enzymes induces immune-mediated regression of murine sarcoma and is a potent adjuvant to dendritic cell vaccination and adoptive T-cell therapy. J Immunother (Hagerstown, Md.: 1997). 2013; 36.

54. Bramante S, Koski A, Kipar A, et al. Serotype chimeric oncolytic adenovirus coding for GM-CSF for treatment of sarcoma in rodents and humans. Int J Cancer. 2014; 135: 720-730.

55. Edris B, Weiskopf K, Volkmer AK, et al. Antibody therapy targeting the CD47 protein is effective in a model of aggressive metastatic leiomyosarcoma. PNAS. 2012; 109: 6656-6661.

56. Li HK, Sugyo A, Tsuji AB, et al. α-particle therapy for synovial sarcoma in the mouse using an astatine-211-labeled antibody against frizzled homolog 10. Cancer Sci. 2018; 109: 2302-2309.

57. Sangiolo D, Mesiano G, Gammaitoni L, et al. Cytokine-induced killer cells eradicate bone and soft-tissue sarcomas. Cancer Res. 2014; 74: 119-129.

58. Tu MM, Lee FY, Jones RT, et al. Targeting DDR2 enhances tumor response to anti–PD-1 immunotherapy. Sci Adv. 2019; 5: eaav2437.

59. Mardiana S, John LB, Henderson MA, et al. A multifunctional role for adjuvant anti-4-1BB therapy in augmenting antitumor response by chimeric antigen receptor T cells. Cancer Res. 2017; 77: 1296-1309.

60. Peinado C, Kang X, Hardamon C, et al. The nuclear factor-κB pathway down-regulates expression of the NKG 2D ligand H60a in vitro: implications for use of nuclear factor-κB inhibitors in cancer therapy. Immunology. 2013; 139: 265-274.

61. Nakashima H, Terabe M, Berzofsky JA, et al. A Novel Combination Immunotherapy for Cancer by IL-13Rα2–Targeted DNA Vaccine and Immunotoxin in Murine Tumor Models. J Immunol. 2011; 187: 4935-4946.

62. Dhupkar P, Gordon N, Stewart J, et al. Anti-PD-1 therapy redirects macrophages from an M2 to an M1 phenotype inducing regression of OS lung metastases. Cancer Med. 2018; 7: 2654-2664.

63. Zheng B, Ren T, Huang Y, et al. PD-1 axis expression in musculoskeletal tumors and antitumor effect of nivolumab in osteosarcoma model of humanized mouse. J Hematol Oncol. 2018; 11: 1-13.

64. Shimizu T, Fuchimoto Y, Fukuda K, et al. The effect of immune checkpoint inhibitors on lung metastases of osteosarcoma. J Pediatr Surg. 2017; 52: 2047-2050.

65. Park JA, Cheung NV. GD2 or HER2 targeting T cell engaging bispecific antibodies to treat osteosarcoma. J Hematol Oncol. 2020; 13: 1-16.

66. Mochizuki Y, Tazawa H, Demiya K, et al. Telomerase-specific oncolytic immunotherapy for promoting efficacy of PD-1 blockade in osteosarcoma. Cancer Immunol Immunother. 2021; 70: 1405-1417.

67. Kawano M, Itonaga I, Iwasaki T, et al. Enhancement of antitumor immunity by combining anti-cytotoxic T lymphocyte antigen-4 antibodies and cryotreated tumor lysate-pulsed dendritic cells in murine osteosarcoma. Oncol Rep. 2013; 29: 1001-1006.

68. Zhou Y, Slone N, Chrisikos TT, et al. Vaccine efficacy against primary and metastatic cancer with in vitro-generated CD103 conventional dendritic cells. J Immunother Cancer. 2020; 8: e000474.

69. Wu W, Jing D, Meng Z, et al. FGD1 promotes tumor progression and regulates tumor immune response in osteosarcoma via inhibiting PTEN activity. Theranostics. 2020; 10: 2859-2871.

70. Jiang K, Li J, Zhang J, et al. SDF-1/CXCR4 axis facilitates myeloid-derived suppressor cells accumulation in osteosarcoma microenvironment and blunts the response to anti-PD-1 therapy. Int Immunopharmacol. 2019; 75: 105818.

71. He X, Lin H, Yuan L, et al. Combination therapy with L-arginine and α-PD-L1 antibody boosts immune response against osteosarcoma in immunocompetent mice. Cancer Biol Ther. 2017; 18: 94-100.

72. Shimizu T, Fuchimoto Y, Okita H, et al. A curative treatment strategy using tumor debulking surgery combined with immune checkpoint inhibitors for advanced pediatric solid tumors: An in vivo study using a murine model of osteosarcoma. J Pediatr Surg. 2018; 53: 2460-2464.

73. Helm A, Tinganelli W, Simoniello P, et al. Reduction of lung metastases in a mouse osteosarcoma model treated with carbon ions and immune checkpoint inhibitors. Int J Radiat Oncol Biol Phys. 2021; 109: 594-602.

74. Wang J, Hu C, Wang J, et al. Checkpoint blockade in combination with doxorubicin augments tumor cell apoptosis in osteosarcoma. J Immunother. 2019; 42: 321-330.

75. Duan XL, Guo JP, Li F, et al. Sunitinib inhibits PD-L1 expression in osteosarcoma by targeting STAT3 and remodels the immune system in tumor-bearing mice. Future Oncol. 2020; 16: 1815-1824.

76. Guiho R, Biteau K, Grisendi G, et al. In vitro and in vivo discrepancy in inducing apoptosis by mesenchymal stromal cells delivering membrane-bound tumor necrosis factor–related apoptosis inducing ligand in osteosarcoma pre-clinical models. Cytotherapy. 2018; 20: 1037-1045.

77. Majzner RG, Theruvath JL, Nellan A, et al. CAR T cells targeting B7-H3, a pan-cancer antigen, demonstrate potent preclinical activity against pediatric solid tumors and brain tumors. Clin Cancer Res. 2019; 25: 2560-2574.

78. Wang Y, Yu W, Zhu J, et al. Anti-CD166/4-1BB chimeric antigen receptor T cell therapy for the treatment of osteosarcoma. J Exp Clin Cancer Res. 2019; 38: 1-11.

79. Fernández L, Metais J, Escudero A, et al. Memory T cells expressing an NKG2D-CAR efficiently target osteosarcoma cells. Clin Cancer Res. 2017; 23: 5824-5835.

80. Huang X, Park H, Greene J, et al. IGF1R-and ROR1-specific CAR T cells as a potential therapy for high risk sarcomas. PLoS One. 2015; 10: e0133152.

81. Rainusso N, Brawley VS, Ghazi A, et al. Immunotherapy targeting HER2 with genetically modified T cells eliminates tumor-initiating cells in osteosarcoma. Cancer Gene Ther. 2012; 19: 212-217.

82. Long AH, Highfill SL, Cui Y, et al. Reduction of MDSCs with all-trans retinoic acid improves CAR therapy efficacy for sarcomas. Cancer immunol Res. 2016; 4: 869-880.

83. Wang Z, Wang Z, Li S, et al. Decitabine Enhances Vγ9Vδ2 T Cell-Mediated Cytotoxic Effects on Osteosarcoma Cells via the NKG2DL–NKG2D Axis. Front Immunol. 2018; 9: 1239.

84. Kiany S, Gordon N. Aerosol delivery of interleukin-2 in combination with adoptive transfer of natural killer cells for the treatment of lung metastasis: Methodology and effect. In: Anonymous Natural Killer Cells: Springer; 2016: 285-295.

85. Udagawa T, Narumi K, Goto N, et al. Syngeneic hematopoietic stem cell transplantation enhances the antitumor immunity of intratumoral type I interferon gene transfer for sarcoma. Hum Gene Ther. 2012; 23: 173-186.

86. Fernandez L, Valentin J, Zalacain M, et al. Activated and expanded natural killer cells target osteosarcoma tumor initiating cells in an NKG2D–NKG2DL dependent manner. Cancer Lett. 2015; 368: 54-63.

87. Le Boeuf F, Selman M, Son HH, et al. Oncolytic maraba virus MG1 as a treatment for sarcoma. Int J Cancer. 2017; 141: 1257-1264.

88. Huijbers EJ, Van Der Werf, Inge M, Faber LD, et al. Targeting tumor vascular CD99 inhibits tumor growth. Front Immunol. 2019; 10: 651.

89. Fang X, Jiang C, Xia Q. Effectiveness evaluation of dendritic cell immunotherapy for osteosarcoma on survival rate and in vitro immune response. Genet Mol Res. 2015; 14: 11763-11770.

90. Yu Z, Qian J, Wu J, et al. Allogeneic mRNA-based electrotransfection of autologous dendritic cells and specific antitumor effects against osteosarcoma in rats. Med Oncol. 2012; 29: 3440-3448.

91. Zhao H, Zhao X, Du P, et al. Construction of random tumor transcriptome expression library for creating and selecting novel tumor antigens. Tumor Biol. 2016; 37: 12877-12887.

92. Ghosh S, Sarkar M, Ghosh T, et al. Absence of CD4 T cell help generates corrupt CD8 effector T cells in sarcoma-bearing Swiss mice treated with NLGP vaccine. Immunol Lett. 2016; 175: 31-39.

93. Kawano M, Itonaga I, Iwasaki T, et al. Anti-TGF-β antibody combined with dendritic cells produce antitumor effects in osteosarcoma. Clin Orthop Relat Res. 2012; 470: 2288-2294.

94. Kawano M, Tanaka K, Itonaga I, et al. Dendritic cells combined with anti-GITR antibody produce antitumor effects in osteosarcoma. Oncol Rep. 2015; 34: 1995-2001.

95. Xu J, Pan X, Zhang S, et al. CD47 blockade inhibits tumor progression human osteosarcoma in xenograft models. Oncotarget. 2015; 6: 23662- 23670.

96. Brennecke P, Arlt MJ, Campanile C, et al. CXCR4 antibody treatment suppresses metastatic spread to the lung of intratibial human osteosarcoma xenografts in mice. Clin Exp Metastasis. 2014; 31: 339-349.

97. Yu G, Li A, Li X, et al. Bispecific antibody suppresses osteosarcoma aggressiveness through regulation of NF-κB signaling pathway. Tumor Biol. 2017; 39: 1010428317705572.

98. Karkare S, Allen KJ, Jiao R, et al. Detection and targeting insulin growth factor receptor type 2 (IGF2R) in osteosarcoma PDX in mouse models and in canine osteosarcoma tumors. Sci Rep. 2019; 9: 1-10.

99. Geller DS, Morris J, Revskaya E, et al. Targeted therapy of osteosarcoma with radiolabeled monoclonal antibody to an insulin-like growth factor-2 receptor (IGF2R). Nucl Med Biol. 2016; 43: 812-817.

100. Mohanty S, Yerneni K, Theruvath JL, et al. Nanoparticle enhanced MRI can monitor macrophage response to CD47 mAb immunotherapy in osteosarcoma. Cell Death Dis. 2019; 10: 1-14.

101. Li B, Wang Z, Wu H, et al. Epigenetic regulation of CXCL12 plays a critical role in mediating tumor progression and the immune response in osteosarcoma. Cancer Res. 2018; 78: 3938-3953.

102. Yahiro K, Matsumoto Y, Yamada H, et al. Activation of TLR4 signaling inhibits progression of osteosarcoma by stimulating CD8-positive cytotoxic lymphocytes. Cancer Immunol Immunother. 2020; 69: 745-758.

103. Gao X, Han D, Fan W. Down-regulation of RBP-J mediated by microRNA-133a suppresses dendritic cells and functions as a potential tumor suppressor in osteosarcoma. Exp Cell Res. 2016; 349: 264-272.

104. Zhou B, Liu M, Qiu X, et al. A novel recombinant immunocasp-6 fusion gene specifically and efficiently suppresses HER2-overexpressing osteosarcoma. Oncol Rep. 2013; 29: 276-282.

105. Liu S, Zheng L, Aweya JJ, et al. Litopenaeus vannamei hemocyanin exhibits antitumor activity in S180 mouse model in vivo. PloS One. 2017; 12: e0183783.

106. Wang C, Lu C, Hsueh Y, et al. Activation of antitumor immune responses by Ganoderma formosanum polysaccharides in tumor-bearing mice. Appl Microbiol Biotechnol. 2014; 98: 9389-9398.

107. Zong S, Li J, Ye Z, et al. Lachnum polysaccharide suppresses S180 sarcoma by boosting anti-tumor immune responses and skewing tumor-associated macrophages toward M1 phenotype. Int J Biol Macromol. 2020; 144: 1022-1033.

108. Zang X, Zhang X, Hu H, et al. Targeted delivery of zoledronate to tumor-associated macrophages for cancer immunotherapy. Mol Pharm. 2019; 16: 2249-2258.

109. Li Q, Hao Z, Hong Y, et al. Reprogramming tumor associated macrophage phenotype by a polysaccharide from Ilex asprella for sarcoma immunotherapy. Int J Mol Sci. 2018; 19: 3816.

110. Wang T, Liu X, Ji Z, et al. Antitumor and immunomodulatory effects of recombinant fusion protein rMBP-NAP through TLR-2 dependent mechanism in tumor bearing mice. Int Immunopharmacol. 2015; 29: 876-883.

111. Li X, Meng Y, Plotnikoff NP, et al. Methionine enkephalin (MENK) inhibits tumor growth through regulating CD4 Foxp3 regulatory T cells (Tregs) in mice. Cancer Biol Ther. 2015; 16: 450-459.

112. Li W, Chen W, Herberman RB, et al. Immunotherapy of cancer via mediation of cytotoxic T lymphocytes by methionine enkephalin (MENK). Cancer Lett. 2014; 344: 212-222.

113. Huang Z, Yang Y, Jiang Y, et al. Anti-tumor immune responses of tumor-associated macrophages via toll-like receptor 4 triggered by cationic polymers. Biomaterials. 2013; 34: 746-755.

114. Barik S, Banerjee S, Mallick A, et al. Normalization of tumor microenvironment by neem leaf glycoprotein potentiates effector T cell functions and therapeutically intervenes in the growth of mouse sarcoma. PLoS One. 2013; 8: e66501.

115. Ghosh S, Sarkar M, Ghosh T, et al. Neem leaf glycoprotein generates superior tumor specific central memory CD8 T cells than cyclophosphamide that averts post-surgery solid sarcoma recurrence. Vaccine. 2017; 35: 4421-4429.

116. Zhang Y, Yuan T, Li Z, et al. Hyaluronate-Based Self-Stabilized Nanoparticles for Immunosuppression Reversion and Immunochemotherapy in Osteosarcoma Treatment. ACS Biomater Sci Eng. 2021; 7: 1515-1525.

117. Takahashi Y, Yasui T, Minami K, et al. Radiation enhances the efficacy of antitumor immunotherapy with an immunocomplex of interleukin-2 and its monoclonal antibody. Anticancer Res. 2017; 37: 6799-6806.

118. Kansara M, Thomson K, Pang P, et al. Infiltrating myeloid cells drive osteosarcoma progression via GRM4 regulation of IL23. Cancer Discov. 2019; 9: 1511-1519.

119. Wang C, Zhou X, Li W, et al. Macrophage migration inhibitory factor promotes osteosarcoma growth and lung metastasis through activating the RAS/MAPK pathway. Cancer Lett. 2017; 403: 271-279.

120. Englisch A, Altvater B, Kailayangiri S, et al. VEGFR2 as a target for CAR T cell therapy of Ewing sarcoma. Pediatr Blood Cancer. 2020; 67: e28313.

121. Liebsch L, Kailayangiri S, Beck L, et al. Ewing sarcoma dissemination and response to T-cell therapy in mice assessed by whole-body magnetic resonance imaging. Br J Cancer. 2013; 109: 658-666.

122. Blaeschke F, Thiel U, Kirschner A, et al. Human HLA-A* 02: 01/CHM1 allo-restricted T cell receptor transgenic CD8 T Cells specifically inhibit Ewing sarcoma growth in vitro and in vivo. Oncotarget. 2016; 7: 443267-43280.

123. Imamura M, Shook D, Kamiya T, et al. Autonomous growth and increased cytotoxicity of natural killer cells expressing membrane-bound interleukin-15. Blood. 2014; 124: 1081-1088.

124. Ojo EO, Sharma AA, Liu R, et al. Membrane bound IL-21 based NK cell feeder cells drive robust expansion and metabolic activation of NK cells. Sci Rep. 2019; 9: 1-12.

125. Evans CH, Liu F, Porter RM, et al. EWS-FLI-1-targeted cytotoxic T-cell killing of multiple tumor types belonging to the Ewing sarcoma family of tumors. Clin Cancer Res. 2012; 18: 5341-5351.

126. Charan M, Dravid P, Cam M, et al. GD2-directed CAR-T cells in combination with HGF-targeted neutralizing antibody (AMG102) prevent primary tumor growth and metastasis in Ewing sarcoma. Int J Cancer. 2020; 146: 3184-3195.

127. Sun L, Li Y, Jiang Z, et al. Vγ9Vδ2 T cells and zoledronate mediate antitumor activity in an orthotopic mouse model of human chondrosarcoma. Tumor Biol. 2016; 37: 7333-7344.
